# Machine learning for RNA sequencing-based intrinsic subtyping of breast cancer

**DOI:** 10.1038/s41598-020-70832-2

**Published:** 2020-08-21

**Authors:** Silvia Cascianelli, Ivan Molineris, Claudio Isella, Marco Masseroli, Enzo Medico

**Affiliations:** 1 Dipartimento di Elettronica, Informazione e Bioingegneria, Politecnico di Milano, Piazza Leonardo da Vinci 32, 20133 Milan, Italy; 2 Candiolo Cancer Institute, FPO-IRCCS, S.P. 142, km 3.95, 10060 Candiolo, TO Italy; 3 Department of Oncology, University of Torino, S.P. 142, km 3.95, 10060 Candiolo, TO Italy

**Keywords:** Computational biology and bioinformatics, Machine learning, Cancer

## Abstract

Stratification of breast cancer (BC) into molecular subtypes by multigene expression assays is of demonstrated clinical utility. In principle, global RNA-sequencing (RNA-seq) should enable reconstructing existing transcriptional classifications of BC samples. Yet, it is not clear whether adaptation to RNA-seq of classifiers originally developed using PCR or microarrays, or reconstruction through machine learning (ML) is preferable. Hence, we focused on robustness and portability of PAM50, a nearest-centroid classifier developed on microarray data to identify five BC “intrinsic subtypes”. We found that standard PAM50 is profoundly affected by the composition of the sample cohort used for reference construction, and we propose a strategy, named AWCA, to mitigate this issue, improving classification robustness, with over 90% of concordance, and prognostic ability; we also show that AWCA-based PAM50 can even be applied as single-sample method. Furthermore, we explored five supervised learners to build robust, single-sample intrinsic subtype callers via RNA-seq. From our ML-based survey, regularized multiclass logistic regression (mLR) displayed the best performance, further increased by ad-hoc gene selection on the global transcriptome. On external test sets, mLR classifications reached 90% concordance with PAM50-based calls, without need of reference sample; mLR proven robustness and prognostic ability make it an equally valuable single-sample method to strengthen BC subtyping.

## Introduction

Breast cancer (BC) is the most common cancer in women worldwide, and in about 80% of cases is invasive, i.e. it breaks through the walls of the glands or ducts where it originated and grows into surrounding breast tissue. Although it is generally referred to as a single disease, BC is heterogeneous in terms of histological composition, molecular features, risk factors, response to treatment, aggressiveness and clinical outcomes^1–5^. The advent of technological platforms for global gene expression profiling has shown more clearly that BC classification and prognosis is not only determined by the commonly used clinical-pathological variables, but also by intrinsic molecular characteristics, which can be probed using molecular methods and genomic profile investigation. The acquired knowledge has improved BC patient management, providing more accurate prognostic stratification.

Based on gene expression quantification, various tests have been introduced in BC clinical practice over the last 10 years to provide molecular stratification and estimate the risk of relapse after surgery, as to avoid adjuvant treatment in low-risk cases^6–8^. However, currently adopted prognostic tests consider a limited number of classifying genes; consequently, the effort to measure gene expression in a tumour sample does not exploit the wider information potentially available through Next-Generation Sequencing (NGS)-based global profiling of RNA expression (RNA-seq). Indeed, some recent works performed BC classification on RNA-seq data^9–11^, but they mostly considered only known marker genes and applied methods developed for previous technologies, like microarrays or PCR, without substantial modifications or adaptations. Conversely, Paquet et al.^12^ developed the Absolute Intrinsic Molecular Subtyping (AIMS), a bioinformatics approach to allow a reproducible single-sample classification of BC profiles, while, Raj-Kumar et al.^13^ proposed a Principal Component Analysis-based approach to improve consistency of subtyping, facing the issue of the non-complete coherence between IHC (immunohistochemistry) and gene expression defined estrogen receptor status; finally, Chen et al.^14^ implemented a deep-learning approach, called DeepType, to learn and cluster a BC gene expression data representation integrating supervised knowledge about subtypes.

Hence, huge dimensionality of exploitable data at reasonable and progressively lower costs, higher accuracy of the expression values, and the chance of multiple surveys on the same data are all crucial reasons of interest for the development of RNA-seq based BC stratification methods for clinical outcome prediction. Combining on a single RNA-seq profile more classifiers, based on different relevant gene signatures and algorithms, could strengthen the results; whereas, existing prognostic tests, examining distinct genes on different platforms, show limited concordance in identifying subgroups and good prognosis patients^8^. To explore in detail the potential of RNA-seq in reconstructing a BC classification system originally developed with a different technology, we considered the so-called *“intrinsic molecular subtypes” (Luminal A, Luminal B, Normal-like, Her2-Enriched and Basal)*, which have become part of the common knowledge on the disease and are recognized as prognostically and therapeutically relevant^7^. Even if these groups firstly emerged by unsupervised hierarchical clustering on global microarray gene expression profiles^1^, BC classification into intrinsic subtypes is primarily achieved by measuring the expression of a set of only 50 genes, the so-called “PAM50 panel”^15^. PAM50 classification is obtained by comparing, for a given BC sample, relative expression of these 50 genes versus a reference sample, and by assigning the subtype based on the highest correlation with the five subtype centroids. Distances from all these centroids can be also used to compute a “Risk of Recurrence” (ROR) score, a prognostic indicator whose low value indicates unlikely relapse after surgery and the possibility to avoid post-operative chemotherapy^15^. The PAM50 assay has been extensively investigated by microarrays and quantitative PCR, and even converted into a Food and Drug Administration (FDA) approved predictive test called Prosigna, working on the Nanostring nCounter platform^16^. The prognostic value of the PAM50 method and its derivatives has been confirmed by independent studies^17–19^. Lately, also RNA-seq profiles have been used for PAM50 classification, mainly based on the algorithm^15^ developed by Parker et al.^9–11, 13^. However, applying a microarray-based classifier to RNA-seq data may provide suboptimal results.

**Figure 1:**
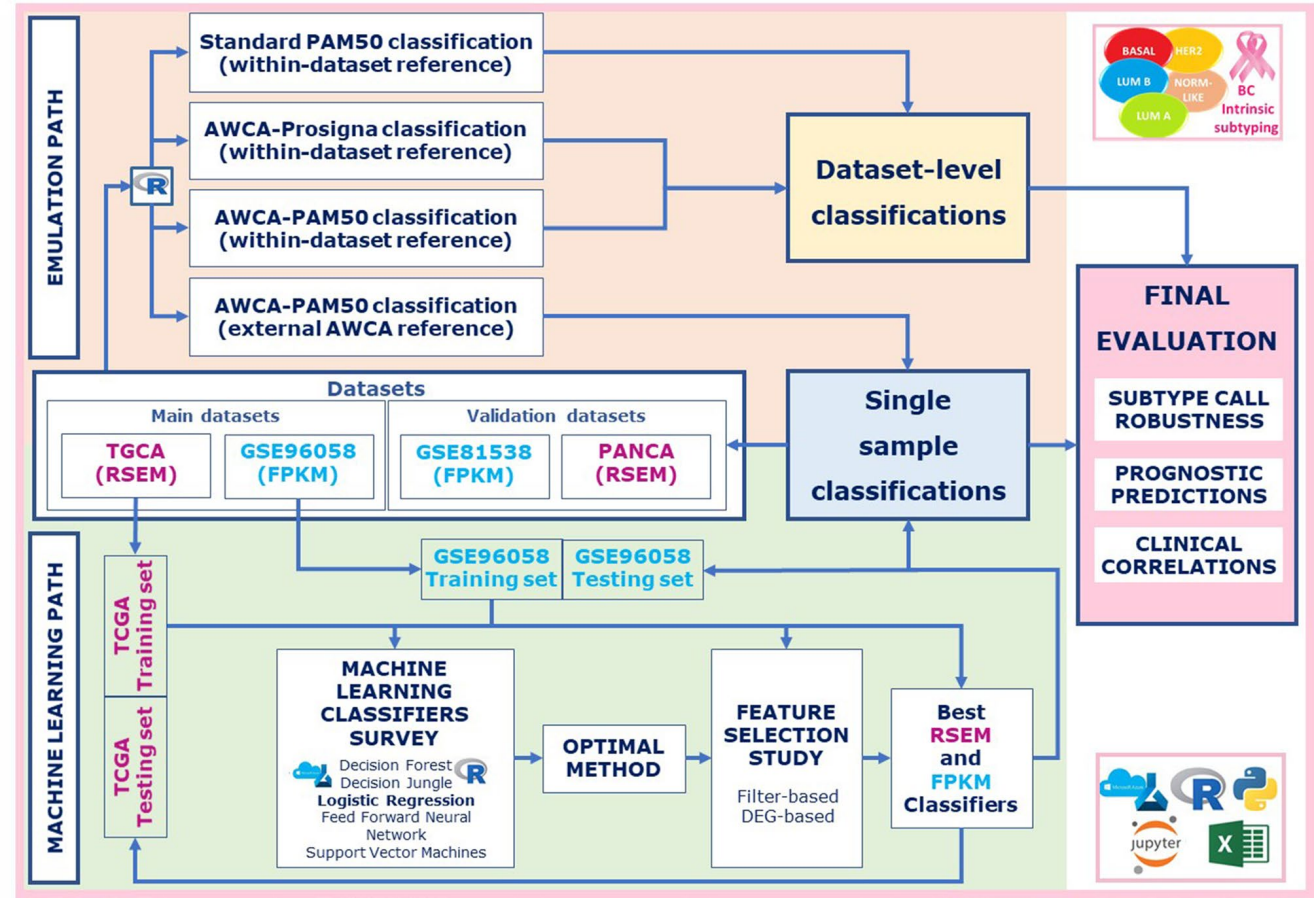
Overview. Main steps of our parallel workflows.

Therefore, here we analyzed the possible limits of the standard PAM50 algorithm when applied to RNA-seq profiles, and explored alternative robust strategies to assign intrinsic subtypes to BC samples, also based on supervised learners and feature selection methods starting from global RNA-seq expression data (Fig. 1). Indeed, intrinsic subtypes summarize BC biological and molecular features, which are known to involve many more genes than the PAM50 set^20^.

## Results

### Assembly of breast cancer RNA-seq datasets

For the purpose of this work we used RNA-seq profiles from four datasets, for a total of 4,731 breast cancer samples (see "Methods" section for details). Notably, data preprocessing was not homogeneous: *TCGA* and *PanCA* expression profiles were subject to RSEM^21^ summarization and upper quartile normalization, while for both GEO datasets only FPKM^22^ expression values were available. From Principal Component Analysis (PCA), performed independently for RSEM and FPKM data, we noticed that subtype distributions were not overlapping on the two cases (Supplementary Material Figure S9). Furthermore, even if some approaches have been proposed to remove specific bias and compare different gene expression data types^21–23^, we did not experience good results in transforming and merging the used RSEM and FPKM datasets (see Supplementary Material Appendix); in our opinion, limited compatibility of RSEM may lie in its peculiar probabilistic approach to handle read mapping uncertainty^21^. Thus, we conservatively performed all analyses separately on RSEM and FPKM profiles, as to evaluate the suitability and reliability of the studied approaches on differently summarized/normalized RNA-seq data.

### PAM50 classification

#### Evaluation of the standard PAM50 method and arisen issues

For all datasets, results of the PAM50 classification performed by the original authors according to the standard method^15^ are publicly available. However, it should be noted that a key step in PAM50 classification could give rise to inconsistency: before calculating distances from subtype centroids, gene expression values for each sample must be transformed into Log2ratios against a reference sample, to be defined for each dataset. Typically, to avoid representation bias, such reference is constructed within the dataset by calculating for each gene the median across a subset of samples with a fixed proportion (60/40) of Estrogen Receptor-positive (ER+) and -negative (ER−) cases, as done for the original PAM50 training^15^. Therefore, calculating the reference from different subsets having the same ER+/ER− composition could yield discordant classification for some samples. To verify this hypothesis, we constructed ten alternative references for the *TCGA* dataset, from ten randomly chosen subsets of 400 samples matching the 60/40 ratio of ER+/ER− cases. Each reference was then used to compute Log2ratio values for all *TCGA* dataset samples, resulting in ten Log2ratio data matrices. As a technical control, one additional Log2ratio matrix was generated against a reference constructed from a subset of 262 samples of the *TCGA* dataset that were originally employed for the same task by Ciriello et al.^9^, and that had a 50/50 ratio of ER+/ER− cases. Then, ten other references and Log2ratio matrices were computed using random subsets having same dimensionality (262 samples) and ER+/ER− proportion (50/50) of the technical control subset. Eventually, PAM50 classification was applied to each Log2ratio matrix using the centroids disclosed by Parker et al.^15^, to assess the concordances of our classifications with the subtype calls published by Ciriello et al.^9^.

Concordance of each of the ten random 60/40 ER+/ER− subset reconstructions with the published classification was suboptimal (mean: 85.52% st.dev.: $+/-$0.83%). Conversely, the ten classifications using references built with subsets having the same dimensionality and ER+/ER− proportion of the technical control subset were much more concordant with the published classification (mean: 95.45% st.dev.: $+/-$1.66%). Eventually, the reconstruction based on the technical control subset of samples employed by Ciriello et al.^9^ was almost completely concordant (99.27%). The minimal discordance can be attributed to the fact that 52 samples of the original set were not included within the available 817 sample *TCGA* dataset under study; therefore, our technical control was not completely identical to the reference of Ciriello et al.^9^. These results confirmed that the choice of the samples used to build the reference significantly affects subsequent subtyping, as much as the adopted reference is different from the one used for the disclosed PAM50 classification. Discordant classifications typically involved samples having comparable correlations with more than one subtype (Supplementary Material Figure S6); the non-separability among subtypes also emerged from PCA analysis (Supplementary Material Figure S9), regardless of data preprocessing. Hence, changes observed in PAM50 classification reflect, to some extent, an internal degree of ambiguity in subtypes; it is plausible that for some samples the boundary between subtypes may be labile due to the possible coexistence of mixed traits.

#### Double averaging for robust reference construction

Having proved that the reference building step affects PAM50 subtyping, we explored an alternative strategy for robust construction of the reference, to improve consistency and reproducibility of PAM50-based subtyping. This iterative strategy starts with a preliminary standard PAM50 classification, and then for each gene computes the average expression within each subtype, taking all the samples of the dataset classified as belonging to that subtype class. The so-obtained within-class mean values are then further averaged for each gene, to obtain a final reference expression value named “average of within-class averages” (*AWCA*), which is independent of the numerosity of samples in each subtype. An *AWCA* can be built without the need of matching exactly a given proportion of cases; double averaging, indeed, equates all class contributions avoiding reference estimation to be corrupted by imbalance distribution of subtypes.

**Figure 2:**
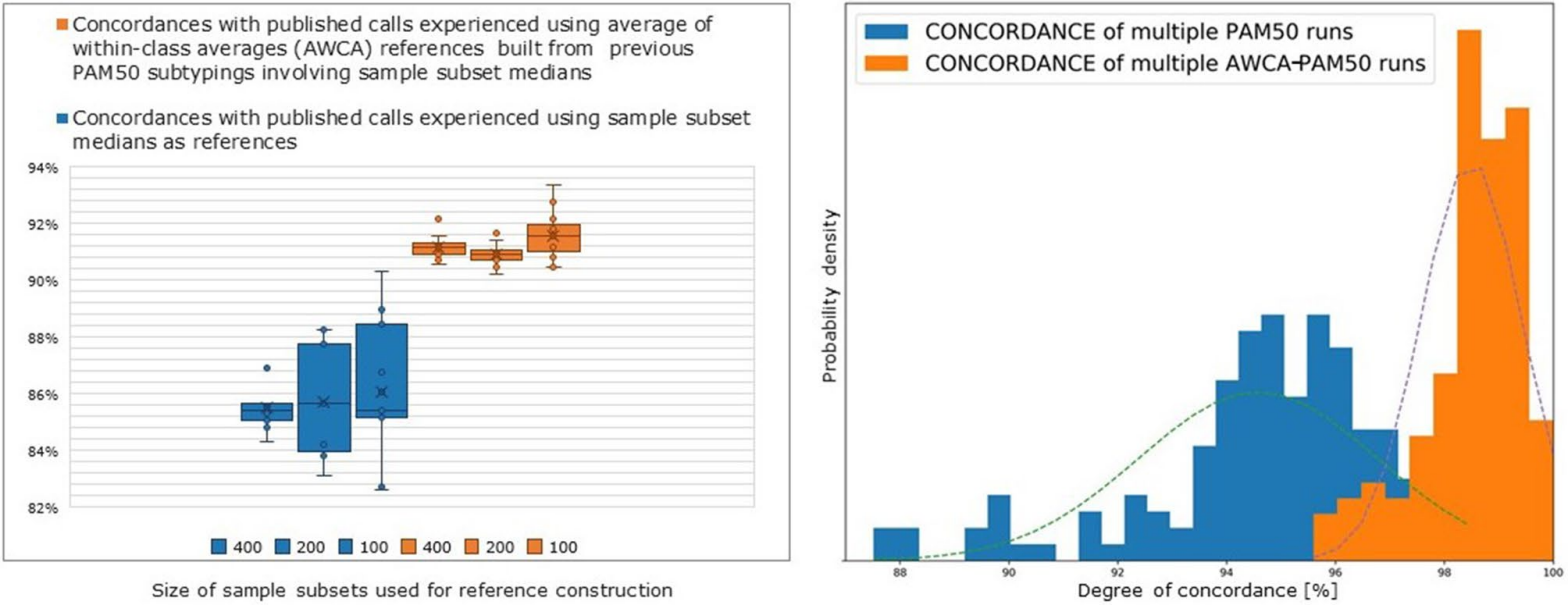
Subtyping of *TCGA* dataset varying the sample subset size of interest for multiple runs of standard PAM50 and AWCA-based PAM50: concordances with Ciriello et al.^9^ subtype calls (left); pairwise concordance distributions (right).

For the *TCGA* dataset, we employed the ten PAM50 classifications obtained from the random 60/40 subsets described above to construct ten new *AWCA* references. In this case, due to the limited number of *Normal-like* samples in the *TCGA* dataset (only 25 samples) and their resulting lack within several of the previously computed random subsets, we decided to exclude the within-*Normal-like* class averaging calculation from the reference computation. We obtained effectively 10 subtyping instances significantly more concordant with the already published calls (91.17% $+/-$0.87%). Notably, discordances with Ciriello et al.^9^ were shared across all, or most of, *AWCA*-based subtyping instances and globally involved only 80 samples, i.e., less than 10% of all samples (Supplementary Material Figure S7). Most importantly, the *AWCA*-based subtyping was highly concordant across the 10 instances (99.13% $+/-$0.43%; Supplementary Material Figure S8) and more stable than the corresponding standard PAM50 one (95.41% $+/-$1.04%), clearly demonstrating that the AWCA-based PAM50 classification is much less dependent on the subset of samples used to build the initial reference. Furthermore, this occurs even when the size of the sample subset selected for reference construction is progressively reduced. Indeed, to further investigate the robustness of *AWCA*-based PAM50 classifications, we built other random 60/40 subsets, varying their overall size from 400 to 25 samples with progressive halving; for each subset size we took ten random subsets, and from the corresponding standard median-based PAM50 classifications we built ten new *AWCA* references. For any of the assessed subset sizes, performing PAM50 classifications with the newly generated *AWCA* references yielded an improvement of approximately 5% of the concordance with the already published calls, with much lower dispersion compared with the results of the corresponding median-based classifications (Supplementary Material Figure S4). Even more importantly, *AWCA*-based PAM50 classifications are much more stable and concordant among them, proving the higher reliability and robustness of the *AWCA*-based approach itself (see Supplementary Material Section S2). Even considering only subset sizes less critical for standard PAM50 classifications (from 400 to 100 samples), as reported in Fig. 2, *AWCA*-based PAM50 classifications show both higher agreement with published calls and better pairwise concordance distribution, with standard deviations always below 1% (98.5% $+/-$0.93%); conversely, agreements of the corresponding standard PAM50 classifications are lower and dispersions almost double at each subset size halving (94.6% $+/-$2.2%).

Such results drove us towards a wider analysis to assess the suitability and possibly the gain of using an inner *AWCA* reference to reproduce PAM50 classification. We used entirely the wide *TCGA* dataset together with the published subtype calls of Ciriello et al.^9^ to calculate the inner *AWCA* reference. Furthermore, since *Normal-like* cases are undersized within the *TCGA* dataset, we built also an *AWCA* reference excluding the *Normal-like* class. Then, we computed corresponding Log2ratio matrices, used for two additional AWCA-PAM50 classifications. As expected, they brought non-complete concordance with Ciriello et al.^9^ calls (Supplementary Material Figure S5), but were significantly accurate (91%, 93%, respectively), although they considered neither the exactly used reference, nor the medians of a subset with a fixed ER+/ER− proportion. In view of the emerged results, we assessed if the use of an inner *AWCA* reference could generally guarantee more than 90% of concordance with respect to already available subtype calls. To this end, we used the *GSE96058* dataset to investigate if this peculiarity could be also found on an independent dataset of differently normalized mRNA-seq profiles. According to the published subtype calls, we computed the *AWCA* reference within *GSE96058*; the subsequent PAM50 classification was again highly concordant (95%) with the published one (see Supplementary Material Section S4). Obtained results confirmed that, in absence of the exactly used reference, inner *AWCA* is a good reference to reproduce subtyping, and to identify stable calls from ambiguous ones.

However, to improve the adoption of a PAM50-based intrinsic subtyping on RNA-seq samples and assure future reproducibility it would be crucial to standardize the approach by defining well-known robust references. In this perspective, we investigated the portability of the already computed *AWCA* references for the subtyping of independent datasets. Since different summarization/normalization strategies, namely RSEM and FPKM, may yield non-comparable results, we took advantage of the *AWCA* reference built using the RSEM values of the *TCGA* dataset for the subtyping of the RSEM *PanCA* dataset, and the *AWCA* reference obtained from the FPKM values of the *GSE96058* dataset for the subtyping of the FPKM *GSE81538* profiles. Taking the published subtype calls as targets, the concordance was beyond 96% both in the *PanCA* dataset using the best *TCGA* *AWCA*, and in the *GSE81538* dataset using the best *GSE96058* *AWCA* (Supplementary Material Figure S15). Notably, when instead an internal *AWCA* was used for subtyping, the concordance with published calls reached almost 95% for both the *PanCA* and *GSE81538* datasets. Furthermore, internal and external AWCA-based classifications appeared highly concordant, with approximately 95% of agreement. These results show that it is possible to use an external reference to center RNA-seq data for robust, single-sample PAM50 classification. However, when RSEM-based *AWCA* was applied to FPKM data, and vice versa, the concordances dropped to 80–87% (see Supplementary Material Appendix). This highlighted to what extent RNA-seq data processing affects data, and indicated that differently normalized profiles should not be merged in a single experimental dataset. Moreover, in some cases, a low number of *Normal-like* samples may justify excluding this class from the *AWCA* calculation, as experienced for the *TCGA* dataset (Supplementary Material Figure S15).

These results confirmed the limits of the standard PAM50 subtyping approach, due to its strict dependence on the reference values and building procedure. Yet, they also opened the perspective of constructing and validating general AWCA references to standardize PAM50-based subtyping, facing the reproducibility and ambiguity issues in subtype calling (Supplementary Material Table S3). At https://github.com/DEIB-GECO/BC_Intrinsic_subtyping we make publicly available the R codes to perform single-sample PAM50 classifications using precomputed AWCA references (for RSEM or FPKM RNA-seq data), and to build AWCA references on any expression data, even from other platforms, as we successfully experienced with microarray data from Affymetrix (see Supplementary Material Appendix). Notably, to obtain valuable AWCA references for single-sample classification of independent expression profiles, we suggest to select a wide dataset subjected to the same normalization of the data of interest, and to compute AWCA references from a subset size of at least 50–100 samples. Hormonal and HER2 status distributions should be also carefully evaluated to check whether they are representative of the typical BC heterogeneity, as we did for *TCGA* and *GSE96058* datasets using ER/PR/HER2 proportions found in the literature as benchmark^24^.

#### Risk of recurrence and prognostic assessment

**Figure 3:**
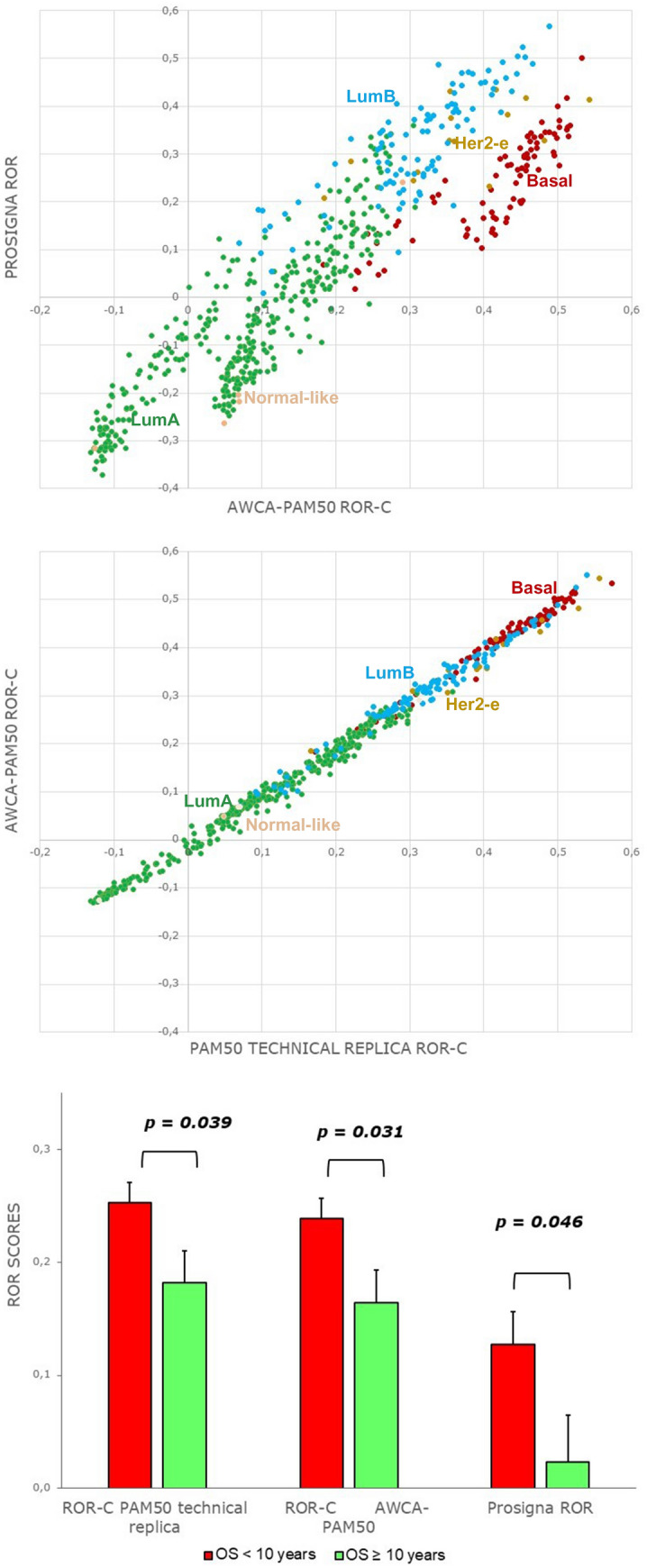
Risk of recurrence. AWCA-PAM50 calls and ROR-C scores compared with Prosigna ROR scores (up) and with PAM50 technical replica scores (center); statistical significance in discriminating 10-year overall survival (OS) status (down).

Here, we report a comparative analysis of the Risk of Recurrence (ROR) scores computed for the *TCGA* dataset downstream of the AWCA-based PAM50 classification and of the PAM50 technical replica, which strictly emulates the Ciriello et al.^9^ PAM50 classification by means of the technical control reference. ROR scores were obtained accordingly to the predictive ROR-C model presented by Parker et al.^15^, and they were tested against 10-year overall survival annotations, to compare the ability of the two PAM50-based approaches in correctly predicting cases with good or poor long-term prognosis. Additionally, we evaluated also another PAM50-based assay, i.e., the Food and Drug Administration approved Prosigna clinical test, which was developed on NanoString expression profiles to provide both the BC subtype and the estimated ROR of a patient. Yet, it requires the proprietary NanoString platform to process each expression profile under exam with an in-vitro reference, included in the Prosigna kit. Hence, in applying in-silico the Prosigna subtyping approach the reference choice issue becomes even harder to face than for the PAM50 subtyping. Furthermore, we did not find public datasets annotated with BC subtypes from the Prosigna test, which prevents a deeper comparative analysis beyond what we discuss. Nonetheless, we performed all the required Prosigna normalization steps, including reference normalization, making use of our precomputed AWCA reference. Then, we implemented the specific Prosigna subtyping procedure and also calculated the ROR scores according to the Prosigna specific ROR model. Eventually, not only the obtained subtype calls were compared with the standard and AWCA-based PAM50 ones (Supplementary Material Figure S11), but also the computed ROR scores. Such comparisons denote a slightly more pessimistic prediction trend for Prosigna, also when tested against effective survival data (Fig. 3). Conversely, ROR scores from standard and AWCA-based PAM50 appeared highly correlated to each other; furthermore, AWCA-based ones improved the prognostic ability (reaching the most statistically significant *p* value) in discriminating good and poor prognosis cases emerged from 10-year overall survival analysis. For further details, please refer to Supplementary Material Section S3.

### Machine learning-based intrinsic subtyping

Machine learning (ML) supervised approaches were separately applied to the wide *TCGA* and *GSE96058* datasets, well representative of BC heterogeneity, to provide valuable classifiers while handling independently RSEM and FPKM mRNA-seq data (further details on Supplementary Materials Appendix). A 220-sample training set was extracted randomly from the *TCGA* dataset, respecting the same 60/40 ER+/ER− proportion of the PAM50 training set. All the remaining 597 cases were instead included in the *TCGA* test set. Both sets were used to train or test the classifiers under study using the Ciriello et al.^9^ subtype calls as target labels. Considering the number of samples in *Her2-Enriched* (65) and *Normal-like* (25) classes, we built the training set including 50 samples for each *Luminal A*, *Luminal B*, *Basal* and *Her2-Enriched* class, plus 20 *Normal-like* samples, as to ensure a good balance of BC subtypes in the learning phase, while keeping out a reasonable number of samples for testing. *Normal-like* class should incorporate only samples from grossly uninvolved tissue and is not widely recognized as prognostically relevant^6^ nor used in ROR models^15, 16^; thus, adding 20 of 25 samples in the training set was not aimed at recognizing this class, but rather at trying to strengthen the ability of the trained classifiers to distinguish other BC intrinsic subtypes from it. Moreover, comparing our classifiers based on whether and which and how many samples were classified as *Normal-like*, allowed us to better assess their subtyping capabilities after proper training. The *GSE96058* dataset was split in a training set of 1,639 samples and a test set with the 1,634 remaining samples. The training set in this case respected the same subtype proportion of the entire dataset (Supplementary Material Figure S13), considering its large size and realistic balance.

#### Survey and selection of the most suitable machine learning algorithm

The following multiclass classifiers were assessed: (1) *Decision Forest*; (2) *Decision Jungle*; (3) *Logistic Regression* (LR); (4) *Feed-Forward Neural Network* (FFNN); (5) *Support Vector Machines* (SVMs). All the mentioned learners were trained in Azure Machine Learning Studio with known ${<}{\hbox {sample, subtype}}{>}$ pairs coming from the training set and had as feature space the entire set of 19,737 genes profiled for the *TCGA* dataset. Indeed, estimating their performances with this huge and noisy feature space offered useful insights about the suitability of each learner in achieving the subtyping task, provided that too strong incidences of overfitting and curse of dimensionality were mitigated by the embedded feature selection approaches, already owned by, or added to, each classifier under evaluation.

**Figure 4:**
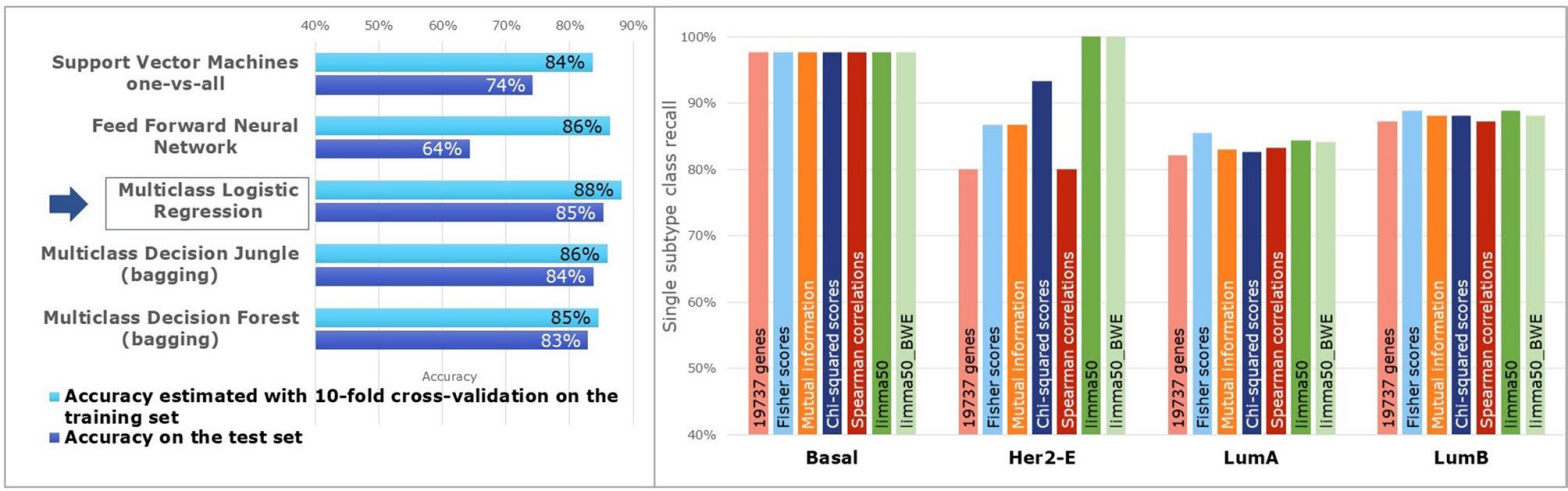
Machine learning survey: classifiers tuned and trained on the *TCGA* training set with tenfold cross-validation and tested on the unseen samples of the test set (left). Feature selection (right): comparison of each class recall on the *TCGA* test set for the mLRs trained on the complete gene set, on the four filter-based spaces and on the limma50 and limma50_BWE gene signatures. *Normal-like* class is excluded from the graph due to the trifling number of samples (only 5) in the *TCGA* test set.

Training was done with the adoption of tenfold stratified cross-validation and hyperparameter grid search to perform model selection, i.e., to set properly, with respect to the final task, all the tunable hyperparameters of each supervised model. Thus, for all ML methods under study, we found best-trained models, i.e., models whose hyperparameter setting and learned parameters led to the best generalization accuracy, estimated through cross-validation. As we can see in Fig. 4, on the left, the results of the ML survey on *TCGA* data indicated a simple regularized multiclass Logistic Regression (mLR) as the most promising method to distinguish intrinsic subtypes. It can set differentially the parameters that directly weigh each gene in each subtype class, while providing at the same time parameter shrinkage to deal with the high dimensionality of RNA-seq data and prevent overfitting. Hence, it has also some points of contact with the nearest shrunken centroid technique, already successfully adopted for the intrinsic subtyping task. Its generalization accuracy of 88%, estimated with cross-validation, was the highest one among all the best-trained learners, even if also the Decision Jungle behaved well, with cross-validation accuracies of 86%.

However, the strength of cross-validation and hyperparameter sweeping was not simply improving the chance of finding the most accurate model for our specific task, but also giving insights of how representative the overall dataset is and how sensitive each model could be to variations in training data or hyperparameter settings. In this context, cross-validation accuracies on different folds were all quite near for the mLR, despite training subset changes and even for several hyperparameter settings, showing the highest robustness among all the assessed classifiers (Supplementary Material Table S4). Moreover, best-trained mLR (with unitary Lasso L1, Ridge L2 hyperparameters) confirmed its primacy also on the unseen samples of the *TCGA* test set, where its accuracy (85%) overcame the ones of all the other best-trained models (Supplementary Material Figures S17–S21). Please refer to Section S5 of the Supplementary Material for further details.

The performances of the regularized mLR were assessed also on the FPKM profiles of the *GSE96058* dataset. We carried out training and test phases as done for the RSEM profiles. The best-trained mLR (L1 = 1; L2 = 0.1) reached even higher accuracies both on cross-validation and on the unseen samples of the *GSE96058* test set (around 89%), confirming the suitability of this regularized classifier also for the BC subtyping of FPKM expression data (see Supplementary Material Figure S32 and Table S6).

#### Feature selection study to improve breast cancer classifier performances

First, as obvious choice, we used the PAM50 gene panel to train mLR models respectively on *TCGA* and *GSE96058* data, as to be able to compare both in cross-validation and testing all the results of our feature selection study with the benchmark performances of the same learner when considering only the genes involved in the original PAM50 method. Such benchmarks are reported in Table 1, together with the results collected with our *AWCA*-based version of the real PAM50 classification, to be compared with the performances reached by the mLRs, when considering several feature spaces of interest.

Regularized mLRs trained on the whole gene sets assigned non-null weights to nearly 1,000 genes, both in *TCGA* and *GSE96058* datasets. Since their accuracies, respectively of 85–89% could be potentially increased tackling the curse of dimensionality, we evaluated if independent feature selection strategies combined with the embedded regularizers could improve mLR results. Particularly, all the adopted feature selection methods simply reduced the original whole feature space to wide relevant gene signatures, and were assessed primarily on *TCGA* data, as RSEM measures are easily comparable across cohorts^21^, while FPKM are not suitable for differentially expressed genes (DEG) analysis.

Initially, we considered external strategies, not involving the learner in the feature selection. We used four filter methods to remove genes supposed not to be meaningful for our subtyping task, based on the next scoring metrics: (1) Fisher scores; (2) Mutual Information; (3) Chi-squared scores; (4) Spearman Correlations. Implementation details are in Supplementary Material Section S5.4.2. We obtained four rankings, each one scoring all the 19,737 genes according to a given metric. For analogy with the previous embedded feature selection, we considered the top 1,000 genes of a scoring metric as a feature space, and we tuned and trained four regularized mLRs, as formerly described. The so-obtained four best-trained models had 89–90% of cross-validation accuracies; tested on *TCGA* test set they reached slightly increased accuracies (86–87%) with respect to those of the mLR (85%) having a 19,737-gene space (Supplementary Material Table S5). We also carefully evaluated confusion matrices, precisions, recalls and switch cases among classes, to consider not only the quantity of discordantly classified cases (all quite comparable), but rather the balanced accuracies (or macro-average recalls) and the type of switches. Chi-square based filter appeared the most valuable since the mLR trained on its top 1,000 genes improved mainly the accuracy of the *Her2-Enrichded* class (93%), compared to mLRs using the other filters (84.5% $+/-$3.16) (see Fig. 4 and Supplementary Material Figures S22-23 and Table S5).

As alternative external feature selection strategy, we used a DEG-based approach. We analyzed the original feature space of 19,737 genes using limma^25^, an R package for the analysis of gene expression data whose core capability is the use of linear models to assess differential expression in multifactor designed experiments. We made differential analyses on the 10 pairwise contrasts between the 5 subtypes; we obtained 10 gene lists, each one including all the genes differentially expressed in a pairwise contrast. Then, given an integer value N, through the union of the top N genes (or of all genes if less than N) from each of the 10 lists, we obtained a set called limmaN, whose genes are all relevant to distinguish at least a couple of subtypes. We tuned and trained again regularized mLRs on limmaN feature spaces obtained for 11 different N values, ranging from 10 to 1,000. The so-trained models reached cross-validation accuracies within 89–95% and accuracies within 84–88% on the *TCGA* test set; best ones are more accurate in cross-validation, and in line in testing, compared to the models trained on the filter-based feature spaces and on the PAM50 panel (Supplementary Material Table S5). Particularly, limma50 was worthy of further investigations, because the mLR considering its 277 genes as feature space reached the best overall accuracy (88%) on the *TCGA* test set and also the highest values for macro-averaged recall (m-aR, 0.94) and precision (m-aP, 0.70) (Supplementary Material Figures S25-S26).

Aiming at further improving prediction performances of the regularized multiclass logistic regression with a combined strategy, we used limma50, top 200 and top 500 Chi-squared-based genes as starting feature spaces for running a wrapper method with sequential backward elimination (BWE). For each starting feature space, we carried out in parallel ten independent runs of backward elimination, performing each run with randomized feature order as to mitigate the bias introduced by the sequential gene scrolling, solvable only through an unfeasible exhaustive search. The algorithm iteratively discards one gene at a time until no more feature elimination improves the accuracy of the regularized mLR model beyond a fixed threshold of gain. Since kept genes in each run were not robust, also due to the needed feature shuffling, ultimately we combined all the genes kept in at least one run, with a downstream preservation strategy. The three gene signatures (210, 165, and 276 genes, respectively) preserved from the three corresponding starting feature spaces were then used as feature spaces for training and testing other regularized mLRs on the *TCGA* dataset (Supplementary Material Table S5). High cross-validation accuracies of 93% and 94%, even higher than using PAM50 genes as features, were respectively obtained using as feature space limma50_BWE and top 500 Chi-squared_BWE. Yet, on testing, each of these models obtained with backward eliminations behaved similarly to the model trained on the corresponding starting feature space. The case of limma50 compared with limma50_BWE clearly appears in Fig. 4, where the accuracy (or recall) of each class due to these two DEG-based signatures are compared with the ones obtained with filter-based approaches.

Hence, limma50 and its further-reduced version limma50_BWE brougth on the *TCGA* cross-validation and testing results in line with our benchmark, i.e., the mLR trained on PAM50 genes (see Table 1 and Supplementary Material Table S5); consequently, they were used to evaluate the suitability and possible improvements in the subtyping task also for FPKM gene expression values. Two regularized mLRs were trained on the *GSE96058* training set and then tested on the test samples of the same *GSE96058* dataset considering each of these two signatures as feature spaces of interest (excluding only the DRAIC gene, since it was unavailable within the *GSE96058* and *GSE81538* profiled genes). Both best-trained models overcame the performances of the model having all the 30865 sequenced genes as feature space, reaching 90% of cross-validation accuracies and 91% of accuracy over the *GSE96058* test set (Supplementary Material Table S6) although their performances were slightly less than the corresponding benchmark, as shown in Table 1.

**Table 1:** Accuracies reached with several intrinsic subtyping methods.

Subtyping method	Feature space of interest	TCGA *cross-validation* (%)	TCGA *test set* (%)	GSE96058 *cross-validation* (%)	GSE96058 *test set* (%)
PAM50*	PAM50 panel	–	92	–	95
mLR	PAM50 panel	92	89	93	93
mLR	All profiled genes	88	85	88	89
mLR	limma50	92	88	90	91
mLR	limma50_BWE	93	87	90	91

* PAM50 applied on test sets only, using precomputed AWCA references.

#### External testing of the logistic regression models

To assess performances on wholly unseen different RNA-seq data subject to the same summarization/normalization, the most accurate mLRs developed for RSEM and FPKM profiles were used to classify respectively *PanCA* and *GSE81538* datasets; all subtyping results are available in Supplementary Material Figures S29–S31, S34–S36, and here summarized in Table 2, where we reported also the concordances with the corresponding AWCA-PAM50 subtypings, based on precomputed references.

Specifically, the models trained on the *TCGA* training set using PAM50, limma50 and limma50_BWE as feature spaces were applied on *PanCA* samples. The found accuracies were nearly the same as the ones obtained on the *TCGA* test set (90%, 88%, 87%, respectively), while the macro-averaged recalls (m-aR) were 0.90 for both limma-based models and 0.88 for the PAM50-based mLR, which showed a lower balanced accuracy on *PanCA* (Supplementary Material Figures S29–S31). Precisions of the *Her2-Enriched* class (the weaker class in testing for all trained models—see Supplementary Material Section S6, Figure S28) eventually increased on *PanCA*, and macro-averaged precisions significantly improved in their turn (m-aP: 0.84, 0.84, 0.83). The same analysis was performed for the models trained on the *GSE96058* training set with PAM50, limma50 and limma50_BWE as feature spaces, and then applied to the *GSE81538* dataset (excluding unavailable genes). For both latter cases, accuracies were just below 90% and all performance metrics (m-aR: both 0.85, m-aP: 0.88 and 0.87) reached slightly lower values than the ones found with corresponding intra-dataset testing on the *GSE96058* test set.

Although the mLR using PAM50 genes reached again higher accuracy, the two limma50-based results, collected for FPKM values, are noteworthy considering that both intra-dataset and external testing dealt with gene expression data different from the RSEM ones employed in the feature selection phase. Furthermore, all the four mLRs computed using limma50 and limma50_BWE genes, and distinctly thought for RSEM and FPKM expression data, reached on testing high concordances with the corresponding AWCA-PAM50 subtypings, even higher than the accuracy with respect to the published labels in the case of the *PanCA* dataset (Table 2). Therefore, these mLRs are provided to classify external BC samples through the R code we make available at https://github.com/DEIB-GECO/BC_Intrinsic_subtyping.

**Table 2:** Concordances with published calls (accuracies) or AWCA-based PAM50 calls for the main mLR classifiers.

Training set	Feature space of interest	Intended for	Accuracy on test set (%)	AWCA-PAM50 concordance on test set (%)	External test set	Accuracy on external test set (%)	AWCA-PAM50 concordance on external set (%)
${TCGA}_{\textit{training}}$	PAM50	RSEM	89	89	PanCA	90	90
${TCGA}_{\textit{training}}$	limma50	RSEM	88	87	PanCA	88	90
${TCGA}_{\textit{training}}$	limma50_BWE	RSEM	87	87	PanCA	87	91
${GSE81538}_{\textit{training}}$	PAM50	FPKM	93	92	GSE81538	92	93
${GSE81538}_{\textit{training}}$	limma50	FPKM	91	91	GSE81538	89	89
${GSE81538}_{\textit{training}}$	limma50_BWE	FPKM	91	91	GSE81538	89	89

### Robustness and prognostic ability of single-sample classifiers

The mLRs trained on different relevant feature spaces reached progressively increasing values for recalls and precisions of all classes (Supplementary Material Figures S27 and S28), though accuracies experienced on internal/external testing did not improve beyond 90% on average, due to the saturation of concordances between mLRs and published calls, occurring also when using PAM50 genes as feature space. However, concordances with target labels can only partially evaluate mLR classifications, since published subtypes are not a gold standard but rather a touchstone to compare performances of the mLR approaches both among them and with the other robust single-sample classifier here proposed, i.e., the AWCA-based PAM50 method with predefined references. Indeed, while in comparing mLR results to the target PAM50 calls Cohen’s kappa agreements were slightly lower than 0.8, the main mLR-based classifications reached valuable pairwise statistic agreements between them, showing Cohen’s kappa values beyond 0.9 for most of the cases (up to 0.925$+/-$0.03 for top1000 Chi-squared and DEG-based feature spaces). Additionally, the mean concordance observed among our three best mLR models (mLR-PAM50, mLR-limma50 and mLR-limma50_BWE) was high (94%$+/-$4%), with limma-based approaches reaching almost perfect agreement and average concordance over 92% with mLR-PAM50. This demonstrates stability of the mLR classifications regardless of the used feature space, provided that it is meaningful with respect to the purpose of BC subtyping. Intriguingly, when we directly compared our best mLR-based classifications with the single-sample AWCA-based PAM50 subtyping, we found the same mean concordance (90% $+/-$2%) experienced with the published PAM50 calls. Furthermore and most importantly, focusing on the disagreements with published subtypes, we found several cases of full concordance among the mLR and AWCA-based PAM50 methods (mainly involving published Normal-like versus Luminal A, or published Luminal A versus Luminal B subtypes); these suggest robustness of the mLR classifiers in calling ambiguous cases, despite their training with published labels.

Additionally, we carefully examined subtype calls assigned with all the single-sample approaches under investigation, including also further classifications obtained with the AIMS method by Paquet et al.^12^. Both on internal and external test sets, we noticed ambiguous samples for all the approaches, confirming the already mentioned degree of ambiguity of intrinsic subtyping. This affects also supervised subtype labels and, in fact, it is partially inherited by mLR classifiers. However, all here proposed methods overcome the AIMS classifications (Supplementary Material Table S11), since its mean concordances with the others single-sample approaches (77%$+/-$4% with AWCA-based one, 79%$+/-$4% with mLR ones) and with the published classifications (77%$+/-$1%) appeared sub-optimal, also beyond the common criticism of ambiguity.

Eventually, we focused on capturing the prognostic potential of our single-sample approaches by comparing 10-year overall survival annotations with the expected clinical outcomes based on the subtype calls. We performed this analysis on *TCGA* and *PanCA* test sets, where sufficient amounts of cases with different 10-year overall survival status were available. Specifically, we considered that each discordance between Luminal A and another subtype implies a different expected prognosis, since Luminal A is well recognized to have the lowest rate of recurrence and the best long-term prognosis^26^. Consequently, in such cases we evaluated whether the discordance improves or worsens the prognostic prediction. First, we focused on the subtype calls discordant with the published ones, and we noticed that both AWCA-based and mLR-based subtypings appeared more reliable in recognizing subtypes with good/poor overall survival prognosis within 10 years, showing an increased prognostic value over standard PAM50 classifications (Supplementary Material Figure S37). Furthermore, we evaluated pairwise discordances between the AWCA-based PAM50 method and each mLR approach; despite only very few cases were available, mLR subtypings seem to slightly improve the prognostic prediction (Supplementary Material Figure S38).

## Discussion

Identification of BC intrinsic subtypes by the PAM50 classifier^15^ has set a cornerstone in cancer genomics and transcriptomics, allowing to link unsupervised, clustering-based class discovery to biological insight and clinically relevant stratification. However, the PAM50 classifier is typically based on centering gene expression values against a reference sample arbitrarily built from the dataset under study; thus, dataset composition and choices for reference construction affect subsequent subtype calling, as we here clearly proved. This limits robustness and reproducibility of the classification, as shown in the present work for RNA-seq data. Hence, as first major achievement of our work, we propose an innovative procedure for reference construction, named *AWCA*, robust to the initial sample selection and improving PAM50 reproducibility, reaching high concordance and stability in classification. The concordance achieved by *AWCA*-based classifications among them is beyond 98%, remarkably improving the stability of some ambiguous samples, i.e., poorly or comparably correlated to more than one class centroid. Moreover, the *AWCA* strategy allows constructing predefined external references (Supplementary Material Table S3) that can be used to classify independent RNA-seq profiles. PAM50 classification of independent sets with external references reached again over 90% of concordance with the published subtypes and over 94% with inner AWCA-based classifications, suggesting the chance of building and setting universal references to increase portability and reliability of the PAM50 subtyping, thus solving its reproducibility issue. However, for single-sample AWCA-based PAM50 subtyping we strongly encourage to employ an AWCA reference subjected to the same normalization procedure of the used expression data, as not to undermine the gain of robustness provided by the approach. Conversely, internal AWCA references can be built on any expression data, even from other technical platforms, as we successfully experienced with microarray data from Affymetrix. Indeed, AWCA-based classifications on Affymetrix log2-transformed data from the GEO dataset GSE4922 improved subtyping stability, with over 96% of mean concordance compared with 88% for corresponding standard PAM50 evaluations. Additionally, we used a so-obtained AWCA reference also as external reference for singe-sample AWCA-PAM50 classification of another Affymetrix dataset (GSE1456), for which PAM50 labels were provided, but no information about the ER status was available to repeat classification or allow internal AWCA-based PAM50 classification; we found subtype calls remarkably more reliable in recognizing samples with good or poor prognoses at medium-long term (see Supplementary Material Appendix). This confirms the subtyping robustness and the key prognostic ability of the here proposed AWCA-PAM50 approach, regardless of the technology used to provide gene expression data.

However, intrinsic subtypes are intrinsically linked both to the molecular traits and to the expression levels of other genes than just the PAM50 panel and are recognized for their prognostic value, also regardless of the PAM50 approach, though it is the most widely method used to recognize them. Thus, exploiting iteratively and also in parallel several classifiers should strengthen the reliability of the subtyping, as in the case of boosting strategies combining weak learners. In this view, we performed also a ML survey considering other classifiers and gene signatures than PAM50, trained supervisely to recognize BC subtypes. A multiclass Logistic Regression appeared the most effective and robust in performing this task, particularly when a feature selection able to provide a feature space of relevant genes is combined with embedded Lasso and Ridge regularizers. Moving beyond PAM50 genes, from our feature selection study we traced two additional promising DEG-based gene signatures, limma50 and limma50_BWE (this latter one from a further backward elimination strategy), including genes meaningfully involved in discriminative patterns between classes and only to a limited extent overlapping with the PAM50 panel (Supplementary Material Table S7).

Our best mLR classifiers reached high accuracy in cross-validation, and valuable performances on internal and external testing, both considering concordances with target labels and with AWCA-based calls (Table 2). Furthermore, on the limma50_BWE feature space both the RSEM and FPKM-based mLRs got almost the same results as on the limma50 feature space, showing classification robustness also in case of a more compact gene set (210 vs. 277 genes). Although limma-derived signatures brought less convincing performances on testing than using mLR on PAM50 genes, or the *AWCA*-based PAM50 method (see PAM50$^{\mathrm{a}}$ in Table 1), it would be overly simplistic to underestimate their interesting results (Table 1) and Supplementary Material Table S8). Indeed: (1) the higher accuracies reached with mLRs using PAM50 genes or with the *AWCA*-PAM50 method are biased by the nature of the published subtype calls, obtained in their turn from the PAM50 assay, using the same gene panel; (2) overall performances of any approach are also influenced by the mentioned ambiguity in subtype calling that possibly affects samples with mixed traits; (3) degradation is caused also by the *Normal-like* class, whose clinical significance remains undetermined^6^, and that could be excluded from a refined version of the intrinsic subtyping approach, as in PAM50-based Prosigna test^16^; (4) the few amounts of training samples and the vast number of features influence subtyping capabilities, mainly for the cases most difficult to recognize. Nonetheless, mLR classifications resulted robust also when varying the feature space under consideration, showing high agreement of the classifications compared both with each others (94%$+/-$4%) and with the AWCA-based PAM50 calls (90%+/2%), especially in cases of discordances with the published calls used for training. Particularly, such agreement found with AWCA-based PAM50 subtyping suggests that mLR approaches, being provided with regularizations to better generalize on independent samples, can in part overcome the flaws of a still limited training set that includes also some incorrect labels for ambiguous samples; thus, the more samples with robust subtype calls will be available, as the ones from AWCA-based PAM50 classifications, the more mLR approaches will certainly improve. Furthermore, mLR approaches can also provide a sample with membership values to each subtype, which could be used in predictive models for clinical outcome or risk of recurrence, as in the case of correlations to each subtype centroid for the already existing ROR models of PAM50 and Prosigna assay. Yet, wide cohorts, well-annotated in terms of relapse events and robust subtype labels, are needed to enhance a preliminary study that gave us encouraging outcomes.

Eventually, all the collected results confirm the suitability and the room for improvement of the mLR as transcriptional classifier to recognize BC subtypes. As experienced for limma50 and limma50_BWE feature spaces, mLR can indeed improve its performances in intrinsic subtyping by exploiting relevantly discriminative parts of the genome-wide information brought by RNA-sequencing, other than the PAM50 panel and despite the inherent bias introduced by the PAM50-based labels, here used to training supervisely and testing. Furthermore, all mLR approaches showed an improved prognostic ability with respect to standard PAM50 calls and further studies could effectively convert their predictive value in reliable clinical outcome estimators.

Thus, in conclusion, the main contribution of this paper is twofold:

1. Propose the *AWCA* reference construction approach to face the proved issues of the standard PAM50 algorithm;
2. Define RNA-seq-based classification approaches to perform single-sample BC intrinsic subtyping with external-AWCA-based PAM50 or regularized mLR methods.

These strategies appeared valuable to favor the use of RNA-seq in BC clinical practice and are worthy of other studies on heterogeneous RNA-seq data, to evaluate and strengthen the reliability of their intrinsic subtyping methods.

## Methods

### Samples and clinical data

Despite BC is one of the cancers with more genomic data available, only a small fraction of public BC RNA-seq data are annotated with PAM50 labels; we used the four of such RNA-seq datasets we could find. The first dataset is part of the Breast Invasive Carcinoma project of The Cancer Genome Atlas (*TCGA*), used within the work of Ciriello et al.^9^ and includes 817 mRNA-seq Version2 RSEM^21^ profiles (http://cbio.mskcc.org/cancergenomics/TCGA/brca_TCGA/). The second dataset, collected under GEO dataset accession number *GSE96058*, includes 3,273 BC RNA-seq FPKM^22^profiles from the Multicenter Sweden Cancerome Analysis Network-Breast Initiative^10^ (https://www.ncbi.nlm.nih.gov/geo/query/acc.cgi?acc=GSE96058). Eventually, we used two additional public datasets to yield a final evaluation of the studied approaches on external data. The first one, called *PanCA* dataset, includes 236 BC samples selected from Pan Cancer Atlas and treated with RSEM pipeline (https://www.cbioportal.org/study/summary?id=brca_TCGA_pan_can_atlas_2018). The second one is a GEO dataset, indicated as *GSE81538* dataset (https://www.ncbi.nlm.nih.gov/geo/query/acc.cgi?acc=GSE81538), and contains 405 BC samples, subject to FPKM normalization. All used expression data were log2-transformed. Further details are in Supplementary Section S1.

### Original PAM50 method and Prosigna test

The original PAM50 method^15^ was developed as a shrunken centroid-based algorithm^27^ for Prediction Analysis of Microarrays (http://statweb.stanford.edu/~tibs/PAM/), focused on 50 genes, known as PAM50 panel. During its training, class centroids were differentially shrunken, identifying subsets of genes that best characterize and contribute to recognizing each intrinsic subtype. We used these centroids, built by Parker et al.^15^ (Supplementary Material Table S2), to replicate the PAM50 algorithm. As required by the PAM50 assay, for each BC sample under exam, the 50-gene expression values were normalized against a calculated reference sample; we compared multiple choices of cohorts and strategies for reference construction. Each time, the nearest centroid rule, with Spearman correlation as similarity metric, was used to assign one of the five mentioned subtypes to each sample. Furthermore, the Cox regression model developed by Parker et al.^15^ was used to estimate the patient’s risk of recurrence score, as a weighted sum of Spearman correlations with subtype centroids and tumor size parameter (ROR-C).

The Prosigna test is a PAM50-based genetic assay used to define a category of metastatic risk at 10 years in hormone receptor-positive women undergoing surgery for invasive BRCA^16^. It focuses on a gene subset of the PAM50 panel called NANO46, and provides both the BC intrinsic subtype and the category of risk of a patient, derived from the estimated risk of recurrence (ROR) score and differentiated also based on lymph node involvement. Both classification and ROR models were independently trained and tested over NanoString profiles, obtained from the proprietary NanoString nCounter platform. Hence, the prognostic assay uses: (1) a normalization pipeline specifically designed for its proprietary technology and a reference included in the Prosigna kit, and consisting of in-vitro transcribed RNA-targets, to be processed together with the sample under study; (2) a Pearson correlation-based Nearest Shrunken Centroid classifier, which excludes the Normal-like class; (3) a proprietary model for ROR score estimation. Here, we used Prosigna centroids and algorithms to assign each sample under study with a corresponding subtype call and ROR score, after normalization against a calculated reference sample.

### Machine learning techniques for breast cancer classification

We performed a machine learning survey, tracing some previous studies^28–32^ for cancer prediction and BC stratification. Accordingly, we assessed several classifiers and embedded regularizations, up to find the most promising approach for the BC intrinsic subtyping task. Specifically, we considered the following techniques, briefly described in Supplementary Material Section S5.2: (1) Multiclass *Decision Forest* as ensemble method with bagging; (2) Multiclass *Decision Jungle* as alternative ensemble method, using directed acyclic graphs (DAGs) instead of trees; (3) Multiclass *Logistic Regression* with both Lasso and Ridge regularizations; (4) Fully connected *Feed-Forward Neural Network* with Ridge regularization, min-max input scaling and sigmoidal outputs; and (5) *Support Vector Machines* with Lasso regularization and One-versus-All approach.

In addition to the mentioned regularizers, we explored further feature selection techniques to handle the high-dimensionality of RNA-seq profiles, facing the curse of dimensionality and the overfitting risk without losing the gene expression interpretation of the maintained features. We evaluated alternatively some filter methods, a differentially expressed genes (DEG)-based approach, and a combined strategy involving a wrapper method. The aim of all these feature selection techniques was distinguishing the informative genes from the non-relevant ones, which behave as noise affecting data and whose removal usually increases or strengthens the predictive power of a classifier. In supervised tasks, filter methods are effective in computation time and robust to overfitting. They score and rank features with respect to the target to be predicted using a statistical measure; accordingly, each feature is either removed or kept in the feature space. The implemented DEG-based approach considers the statistical significance of gene expression variances within or between classes to trace relevant features according to discriminative patterns. Eventually, the applied combined strategy is focused on a wrapper method with sequential backward elimination as heuristic approach. Wrapper methods consider feature selection as a search problem: during the learning phase, different combinations of features are compared based on the cross-validation performances of the chosen model, up to finding a reduced set of relevant features. However, due to the prohibitive computational cost for high-dimensional spaces, we applied this strategy on some promising already reduced gene sets, rather than on all the profiled genes.

To asses alternative classifiers and feature selection techniques, we worked jointly on RStudio (http://www.rstudio.com/) and Azure Machine Learning Studio (https://studio.azureml.net/), an integrated development environment working on the Azure cloud service platform. For further implementation details, please refer to Section S5 of the Supplementary Material.

## Supplementary information

Supplementary Information.

## Data Availability

The R code to generate AWCA references, to use AWCA-based PAM50 with precomputed external references and to use the mLR-based BC classifiers is available at https://github.com/DEIB-GECO/BC_Intrinsic_subtyping.
