# Supplementary material for: Machine learning for RNA sequencing-based intrinsic subtyping of breast cancer
Source: Sci Rep. 2020 Aug 21;10:14071. doi: 10.1038/s41598-020-70832-2 (PMC7442834; doi:10.1038/s41598-020-70832-2)
Supplement: Supplementary file 1 — Supplementary Information. [file 41598_2020_70832_MOESM1_ESM.pdf]

Supplementary Material

Machine learning for RNA sequencing-based intrinsic subtyping of breast cancer

Silvia Cascianelli[1,*], Ivan Molineris[2], Claudio Isella[2],

Marco Masseroli[1,†] and Enzo Medico[2,3,†*]

[1] Dipartimento di Elettronica, Informazione e Bioingegneria, Politecnico di Milano, Piazza Leonardo da Vinci 32, 20133, Milan, Italy.

[2] Candiolo Cancer Institute, FPO-IRCCS, S.P. 142, km 3,95, 10060 Candiolo (TO), Italy.

[3] Department of Oncology, University of Torino, S.P. 142, km 3,95, 10060 Candiolo (TO), Italy.

* To whom correspondence should be addressed

† Both authors share senior authorship.

Index

S1 Datasets ... 3

S1.1 TCGA - Breast Invasive Carcinoma ... 3

S1.2 GEO dataset GSE96058 ... 3

S1.3 PanCA dataset and GEO dataset GSE81538 ... 3

S2 PAM50 emulation ... 5

S2.1 Missing gene expressions ... 5

S2.2 Subtyping on TCGA dataset ... 5

S3. In-silico Prosigna emulation and Risk of Recurrence estimation ... 11

S3.1 Prosigna test and Risk of Recurrence models ... 11

S3.1.1 Data pre-processing and normalization ... 11

S3.1.2 Prosigna classification with the Nearest Shrunken Centroid method ... 12

S3.1.3 Prosigna classification of the TCGA dataset ... 12

S3.2 Conclusions from the Prosigna emulation and Risk of recurrence comparative analysis ... 13

S4 PAM50 classifications of the GSE96058 dataset ... 16

S4.1 PAM50 classifications with internal or external *AWCA* references ... 17

S5 Machine learning path ... 19

S5.1 Machine learning survey and embedded regularization ... 19

S5.2 Classifiers under evaluation ... 20

S5.3 Training phase and classifier survey ... 22

S5.4 Feature selection strategies ... 26

S6 Performances of the main Logistic Regression models under evaluation ... 28

S7 Final recap and comparisons of the main intrinsic subtyping approaches ... 39

S7.1 Robustness and concordance evaluation between main intrinsic subtyping approaches ... 40

S7.2 Prognostic assessment ... 41

Appendix ... 43

RSEM and FPKM data cross-comparative evaluations ... 43

AWCA-based PAM50: an additional use-case on microarray data ... 44

References ... 45

S1 Datasets

S1.1 TCGA - Breast Invasive Carcinoma

The expression dataset of interest includes 817 RNA-seq Version2 RSEM4 profiles of breast invasive cancer samples, subject to upper quartile normalization and log2-transformation. Based on Ciriello *et al*. (2015) supplementary materials, RNA sequencing was performed at the University of North Carolina at Chapel Hill on the Illumina HiSeq 2000; the RNA-seq profiles under study were obtained from levels of transcripts sequenced genome-wide with the Illumina mRNA-seq method and processed as follows. Resulting sequencing reads were aligned to the human hg19 genome assembly using MapSlice. Gene expression was quantified for the transcript models corresponding to the TCGA GAF 2.13 using RSEM4 and normalized within samples to a fixed upper quartile (UQ normalization). For our analyses, Upper quartile normalized RSEM data were log2 transformed. Genes with a value of zero following log2 transformation were set to the missing value, and genes with missing values in more than 20% of samples were excluded from downstream analyses, in compliance with what Ciriello *et al*. did in their work in 2015. Thus, ultimately, we used a dataset comprising 19,737 genes in each sample. Furthermore, Ciriello *et al*. performed a standard PAM50-based classification for the samples of the dataset, whose subtype calls are reported in Table S1.

S1.2 GEO dataset GSE96058

Illumina paired-end mRNA-sequencing and expression estimation were performed for a cohort of 3,273 breast cancer samples from the Multicenter Sweden Cancerome Analysis Network-Breast Initiative (Brueffer *et al*., 2018) and collected under GEO dataset accession number GSE96058. Gene expression data is made of FPKM profiles generated using Cufflinks 2.2.1 software. The resulting data was post-processed by collapsing on 30,865 unique gene symbols (sum of FPKM values of each matching transcript), adding to each expression measurement 0.1 FPKM, and performing a log2 transformation. PAM50 subtyping was performed by Brueffer *et al*. according to the standard PAM50-based classification, using a fixed reference selected to match the original cohort used in Parker *et al*. (2009). The distribution of subtype calls is reported in Table S1.

S1.3 PanCA dataset and GEO dataset GSE81538

The PanCA dataset includes 236 breast cancer mRNA-seq profiles selected from Pan Cancer Atlas, without overlaps with the samples already included in the used TCGA dataset. They were treated with RSEM pipeline, UQ-normalized, and log2-transformed. The GSE81538 dataset, instead, contains 405 breast cancer mRNA-seq profiles subject to FPKM normalization and log2-transformation and collected under GEO dataset accession number GSE81538. For both datasets, published subtype calls, assigned according to standard PAM50-based classifications by the original collectors, are available and reported in Table S1.

Table S1. Dataset characteristics and compositions according to the published PAM50 classifications: Luminal A (LumA), Luminal B (LumB), Her2-Enriched (Her2) Basal and Normal-like.

Dataset	Tot RNA-seq profiles	Data normalization	*LumA*	*LumB*	*Her2*	*Basal*	*Normal-like*
**TCGA**	817	RSEM	415	176	65	136	25
**GSE96058**	3,273	FPKM	1,657	729	327	339	221
**PanCA**	236	RSEM	131	32	16	43	14
**GSE81538**	405	FPKM	156	105	65	57	22

Figures S1 and S2 show the use of the described datasets in the computational path following illustrated.

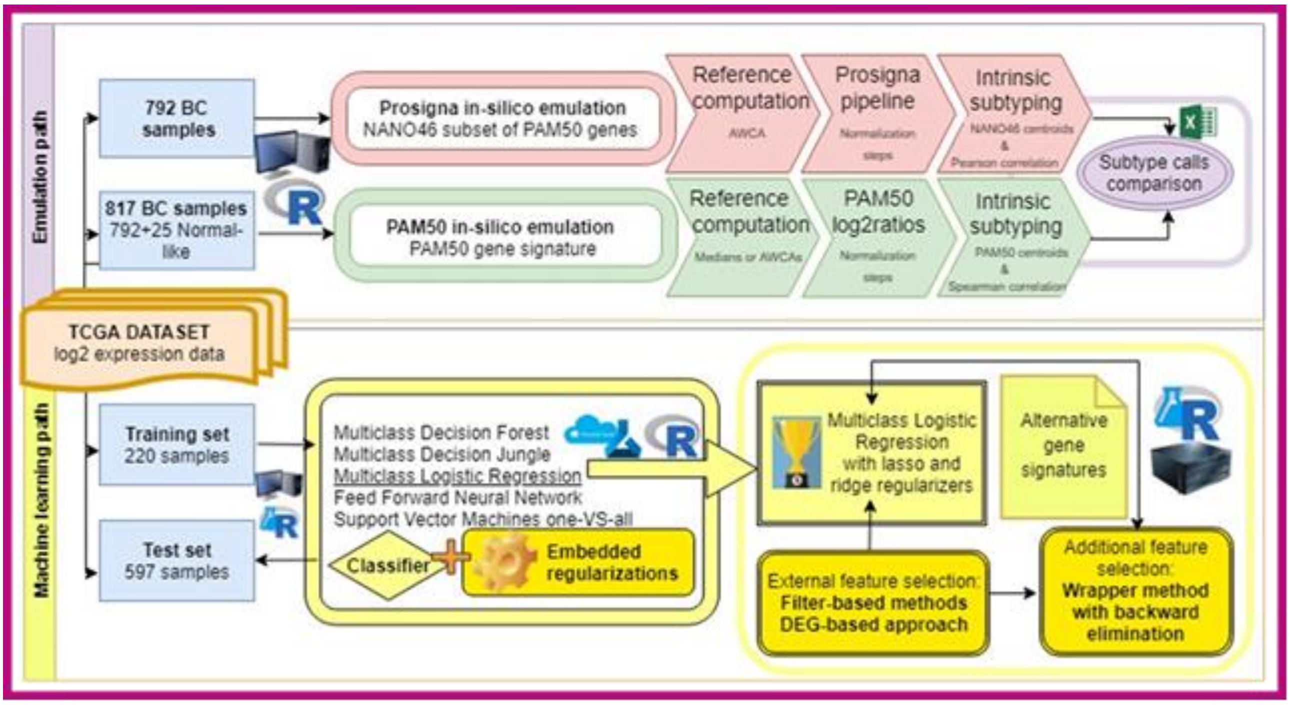

Figure S1. Emulation and machine learning paths. Parallel workflows over the TCGA dataset.

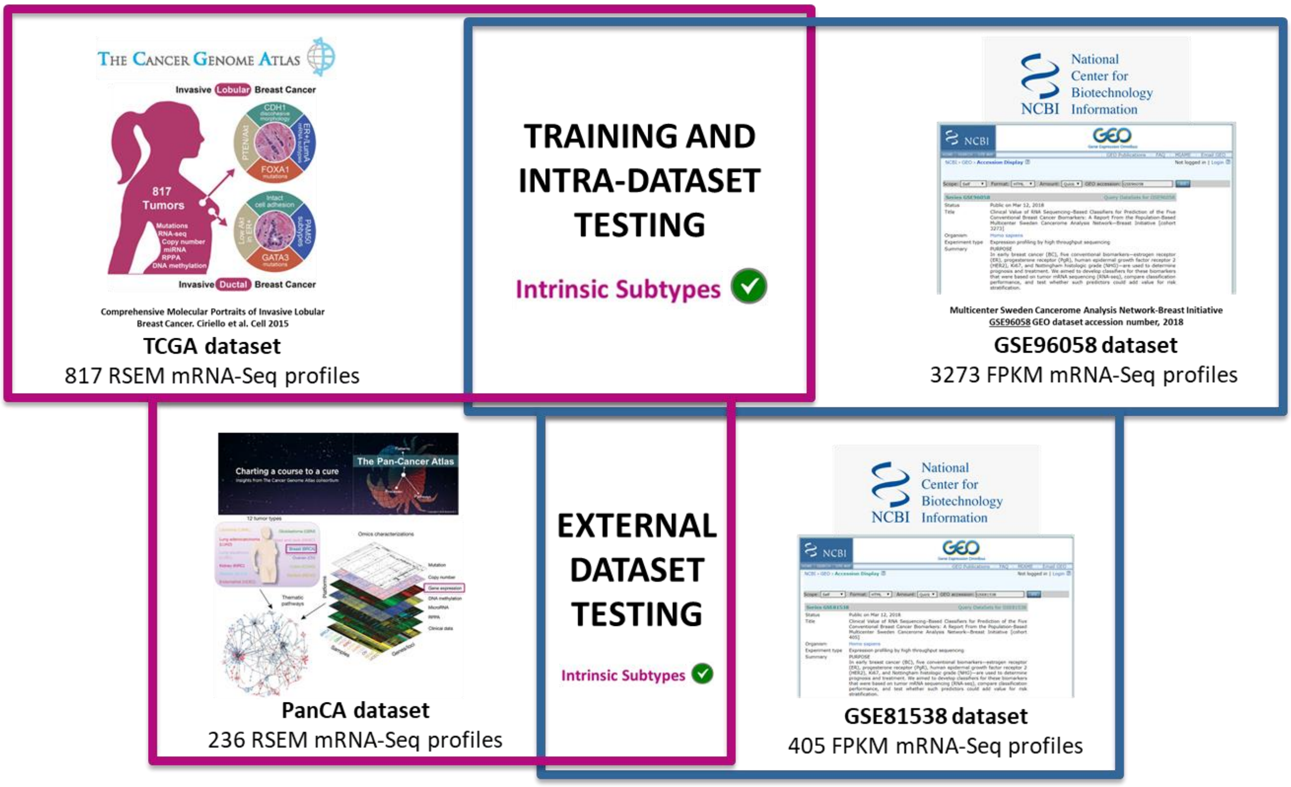

Figure S2. PAM50-based and machine learning paths. Roles of all the available datasets using their disclosed subtype calls and target labels.

S2 PAM50 emulation

S2.1 Missing gene expressions

For our PAM50 emulation, we needed to restrict the gene set of interest to the PAM50 panel. Consequently, in whichever case we had to face a missing gene expression data issue, we solved it on log2-space as follows. Missing log2-values were excluded from reference sample estimation by defining modified functions that compute mean, median and standard deviation over a restricted subset of all the values. In this way, whether a specific gene had an NA (not available) value among one of the samples under examination, the contribution of that sample for that specific gene would be discarded from the computation of the overall mean, median, or standard deviation. On the contrary, to compute the correlations between a sample profile and each subtype centroid, we substituted missing values with zero-values, since each NA value was in its turn caused by the absence of the absolute gene expression value for that specific gene in the involved sample. This replacement is a standard use also in other pipelines for the treatment of RNA-seq counts and in NanoString data handling, where it is suggested to substitute null or below 1 absolute gene expression values with a unitary constant bias (i.e., a null log2-value).

S2.2 Subtyping on TCGA dataset

PAM50 classifications were performed using the class centroids developed by Parker *et al*. in 2009 and reported in Table S2.

Table S2. PAM50 centroids by Parker et al. (2009).

PAM50 GENES	Basal	Her2-Enriched	Luminal A	Luminal B	Normal-like
ACTR3B	0.71833189105221	-0.481665674726704	0.0099810704381048	-0.190551327982217	0.465722870515964
ANLN	0.537372300595531	0.26693160932886	-0.57924571615828	0.0988041789187431	-0.83693959305506
BAG1	-0.574506867003171	-0.476072868053812	0.758221161127353	-0.405458622327578	0.316552972849696
BCL2	-0.118760430362242	-0.157913959232179	0.287487439627067	-0.44133949784535	0.533978871455655
BIRC5	0.300488641307438	0.405733099101299	-0.881434366334594	0.603850776734403	-0.876636423925443
BLVRA	-0.642677513396256	0.335336040994047	0.0420420167875037	0.691204961687021	-0.163412811613957
CCNB1	0.191208143233350	0.135476651890144	-0.491662113750233	0.503176357566503	-0.545269311937199
CCNE1	0.5602710279181	0.0668722320900592	-0.430291227412725	-0.016661429525915	-0.255476058116241
CDC20	0.399695241707236	0.00835552010412316	-0.469044010265104	-0.0704124657384466	-0.0455048098566988
CDC6	0.159418279239843	0.589006820321944	-0.61282430546711	0.510895969130001	-0.595752175354644
CDCA1	0.472400167554248	-0.0238192070320764	-0.712520818851019	0.589626882663298	-0.370533364608738
CDH3	0.508362012467715	0.210889691612697	-0.513649344383634	-1.41913443744317	0.757920623508959
CENPF	0.482976287851816	-0.0292661598656275	-0.54374023405061	0.278228556393300	-0.0705830752611994
CEP55	0.567748893765426	0.276381021673186	-0.746721735125358	0.460015762468336	-1.16237418628659
CXXC5	-0.92038581344894	-0.241550612126531	0.467411570831134	0.321335019875456	0.0509014360369965
EGFR	-0.0304168492933523	-0.0963826205261192	0.00916296285683097	-0.412401259011741	0.341637082320563
ERBB2	-0.808353979685898	1.75984423053252	0.608191264034737	0.159651873930408	-0.870238456252032
ESR1	-2.74651308572764	-1.51311125337343	2.16141188167927	1.60589991409782	-0.418282349385733
EXO1	0.42809035571695	0.0492971938541269	-0.567474505364458	0.141241281899209	-0.4507805368292
FGFR4	-0.271238025272568	0.821778152444019	0.170811924512710	-0.247036037777853	0.857472776645833
FOXA1	-2.62694672123675	0.0228271511969584	1.0174574205457	0.360757794789733	-0.782812106272943
FOXC1	1.49045147226633	-0.947174191636912	-0.174957960004945	-1.56485496402643	1.11154786423416

GPR160	-1.05497467201534	0.583194826166968	0.685489972807875	0.714407601174597	-0.423568467196315
GRB7	-0.276128585792610	1.03065778049362	0.0415689860888212	0.0877508871181497	0.241710990681836
KIF2C	0.203572580112815	-0.165102048401172	-0.505394668119272	-0.18289071282855	-0.390014483980550
KNTC2	0.600356166736404	0.0425467918407693	-0.58822098932071	0.386706843524287	-1.06962886016236
KRT14	0.0968267225951578	-0.443646142118489	0.368375943286720	-0.639446965945955	1.73568631016155
KRT17	0.482565528200505	-0.337837101113602	0.0142098620606827	-1.46374293365444	1.75959843723177
KRT5	0.506640416426025	-0.42826177758168	0.215320067973960	-0.911607270494262	1.78511689525264
MAPT	-0.42582927334418	-0.357506541134998	0.700622717588887	-0.19034057442467	0.117828498493485
MDM2	-0.251366205388229	-0.106728680574222	0.141957430453390	-0.133779036556646	0.274214010559593
MELK	0.523033872432266	0.198013114679137	-0.58208810796246	0.447934629706117	-0.743764675552645
MIA	1.57827636824347	-0.90489862111629	-0.165258584299697	-1.42292626721177	2.03885955577086
MKI67	0.476537447819512	0.065662359566943	-0.501871622444359	-0.145217867594777	-0.166004063281183
MLPH	-0.339972458833524	-0.195228657981430	0.339304417889105	-0.456149914938842	0.750758364744867
MMP11	-0.556037671980059	0.5067587600346	-0.00625509011475386	0.33419930855919	-2.32698511905842
MYBL2	0.389893451959192	0.205263580428381	-0.84356993132705	0.467281990134531	-0.601704754297017
MYC	0.178763812453212	-1.04683283160474	-0.090830821420967	0.0152643973204342	1.02917620291809
NAT1	-0.936848945968056	-0.0899884918164858	2.92278679191125	0.470788041948480	-0.363273764184013
ORC6L	0.216304796525868	0.204402449297876	-0.352220666898442	0.110627650250106	-0.255879493250552
PGR	-0.429133388609156	-0.279409915717406	0.445785002691586	-0.448839843849832	0.126011481678608
PHGDH	0.634518869785175	-0.186625862203220	-0.398682233972277	-1.03013931821750	0.66043775260378
PTTG1	0.264131894483995	0.055809894817994	-0.634468270258443	0.249725280730980	-0.549781259548456
RRM2	0.156204675747248	0.68272488919714	-0.950760200359295	0.350663839755693	-1.12105492950754
SFRP1	0.98798845910263	-1.04820266695367	0.131566363888596	-1.72045826151304	2.43628866770475
SLC39A6	-1.05112505157325	-0.695736456552694	2.06145907498356	1.65330302214459	0.116889693935195
TMEM45B	-1.10945818050443	1.33063617180581	0.446242044594737	0.375688226418575	0.0362089142863473
TYMS	0.449800897311564	0.05294489694821	-0.644602075052837	0.492606521200551	-0.726989454321298
UBE2C	0.218534146535747	0.0610805976997803	-0.519818399226962	0.292799305558851	-0.40889468512475
UBE2T	0.389908898288675	0.28453681332228	-0.539259390713988	0.738952133209629	-0.952381005370445

Performing PAM50 classification using ten references computed as medians of ten random subsets with 60% ER+/40% ER- proportion did not bring complete nor even over 90% of concordance with the subtype calls of Ciriello *et al*. (2015) (84%-87%). On the contrary, using the medians of ten random subsets with the same balanced ER+/ER- proportion of the subset of samples employed by Ciriello *et al*. (2015), we found an average concordance of 95%. Finally, using a subset built by including 262 samples within the specific sample set selected by Ciriello *et al*. (2015) brought nearly perfect concordance (99.3%), although we had to exclude from the median computation 52 samples used in Ciriello *et al*. (2015) but not available within the 817 under study.

Then, ten additional PAM50 classifications were performed with references built as an average of within-class averages (*AWCA*s), using each time as starting class assignments the subtype calls obtained from the previous PAM50 classifications involving the random subset with 60% ER+/40% ER-. The process is schematized in Figure S3. This reference building procedure aims to increase robustness through averaging, and effectively led to significantly more stable and accurate results (91%-93%) in the corresponding PAM50 classifications, even without any care to the originally employed subset chosen by Ciriello *et al*. (2015).

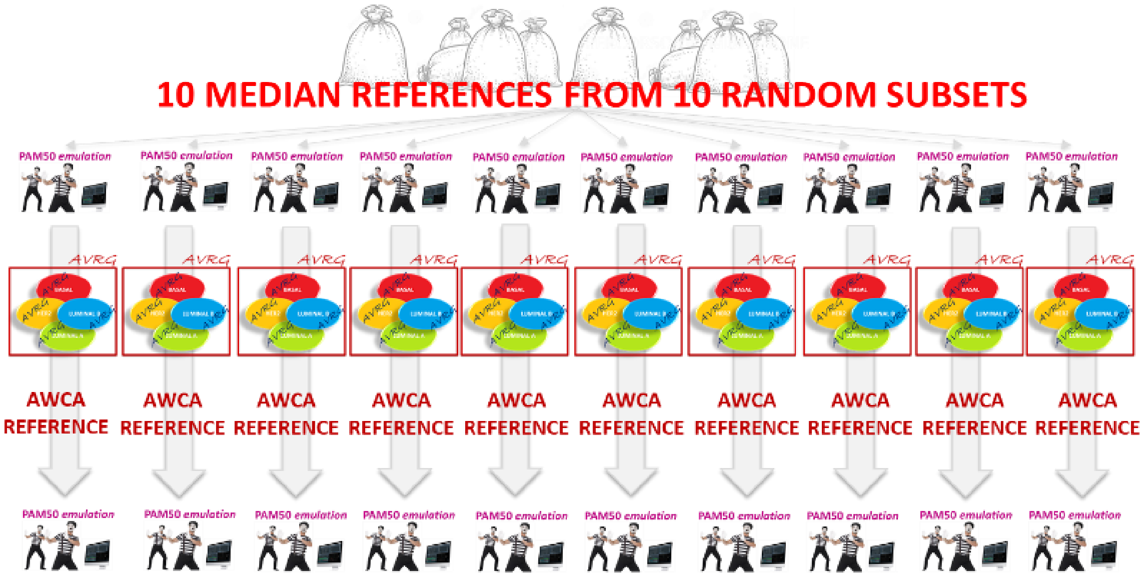

Figure S3. A robust method (AWCA) to calculate the PAM50 reference and improve subtyping consistency.

Eventually, we built other random 60% ER+/40% ER- subsets with progressive size halving (from 400 to 25 samples), as to investigate the robustness of AWCA reference construction. For each size, from ten random subsets we computed the corresponding median-based PAM50 classifications and we used them to build ten new AWCA references. For any size, PAM50 classifications based on the newly generated AWCA references showed much lower dispersion and were approximately 5% more concordant with the already published calls than the corresponding median-based classifications (Figure S4). Furthermore, AWCA references brought robust results with subsets of 200 samples and even using 50 samples only; conversely, median-based PAM50 classifications needed a subset of 400 samples to reach stable results, while subsets of only 50-25 samples become really critical.

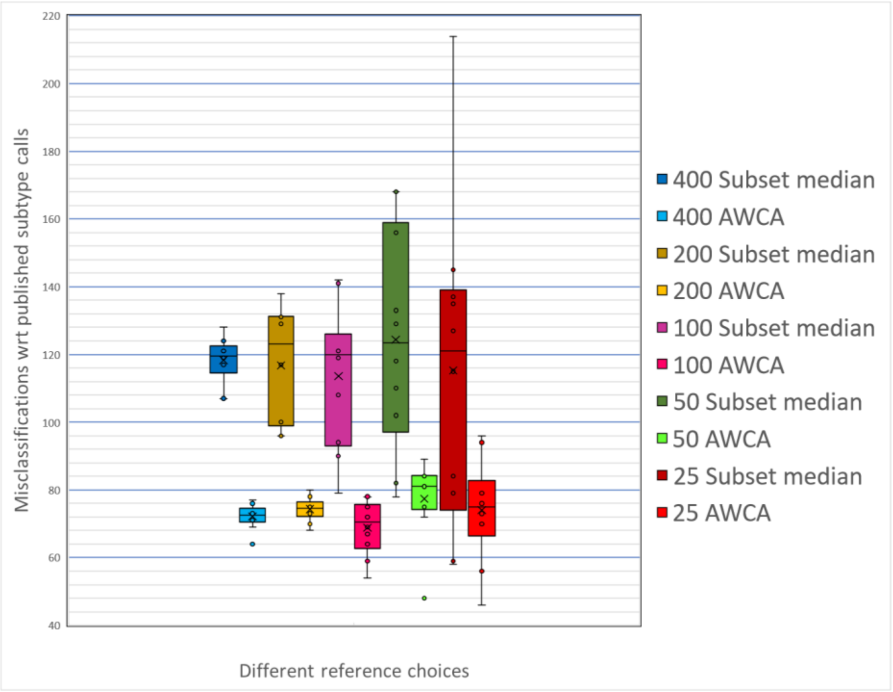

Figure S4. Discordances with published calls: distributions after AWCA-*based or median-based classifications, for different sizes of subsets involved in reference construction.*

Considering that the starting subtype assignments do not affect relevantly the final PAM50 classification in case of the *AWCA* strategy, we eventually built two average references within the TCGA dataset, according to the published subtype calls of Ciriello *et al*. (2015): one excluding Normal-like class and one including it. These *AWCA*s were used for two other PAM50 classifications of the TCGA dataset. Although the reference built as the average of only the 4 intrinsic subtype classes led to higher classification accuracy, in both cases the overall error rate fluctuated between the 7% and 9%, keeping over 90% of concordance. Non-concordant calls involved primarily Luminal A (LumA) and Luminal B (LumB) classes, sharing some similar molecular traits from being Luminal tumors; furthermore, most parts of the disagreements were coincident with the ones already found with the previous PAM50 classifications, based on the subsets under study. Confusion matrices are reported in Figure S5.

REFERENCE: Average of within-class averages built A-POSTERIORI including Normal-like class

	Classified as Basal	Classified as Her2	Classified as LumA	Classified as LumB	Classified as Normal-like	Recall	Recall %
Ciriello et al. Basal	**136** 100.0%					136/136	100.0%
Ciriello et al. Her2		**65** 100.0%				65/65	100.0%
Ciriello et al. LumA		**4** 1.0%	**348** 83.9%	**61** 14.7%	**2** <1%	348/415	83.9%
Ciriello et al. LumB		**3** 1.7%		**173** 98.3%		173/176	98.3%
Ciriello et al. Normal-like	**4** 16.0%	**3** 12.0%			**18** 72.0%	18/25	72.0%
Precision	136/140	65/75	348/348	173/234	18/20	**817**	
Precision %	97.1%	86.7%	100.0%	71.2%	90.0%		

PAM50 accuracy wrt published subtype calls: **90.6%**

REFERENCE: Average of within-class averages built A-POSTERIORI excluding Normal-like class

	Classified as Basal	Classified as Her2	Classified as LumA	Classified as LumB	Classified as Normal-like	Recall	Recall %
Ciriello et al. Basal	**136** 100.0%					136/136	100.0%
Ciriello et al. Her2	**2** 3.1%	**60** 92.3%	**1** 1.5%		**2** 3.1%	60/65	92.3%
Ciriello et al. LumA		**1** <1%	**395** 95.2%	**8** 2.0%	**11** 2.7%	395/415	95.2%
Ciriello et al. LumB		**5** 2.8%	**25** 14.2%	**146** 83.0%		146/176	83.0%
Ciriello et al. Normal-like	**1** 4.0%				**24** 96.0%	24/25	96.0%
Precision	136/139	60/66	395/421	146/154	24/37	**817**	
Precision %	97.8%	91.0%	93.8%	94.8%	64.9%		

PAM50 accuracy wrt published subtype calls: **93.2%**

Figure S5. PAM50 classifications of the TCGA dataset using average sample references (AWCA).

All previous results confirmed that the samples used for building the reference may affect meaningfully the subsequent PAM50 classification and may lead to some inconsistencies in multiple instances of subtype calling. However, most of the inconsistencies were widely recurrent across reference changes, and discordant classifications typically involved samples showing comparable correlations with more than one subtype, as clearly emerged from Figure S6. Notice that the average correlations are here computed considering the ten PAM50 classifications using as references the medians of different 60% ER+/40% ER- subsets (subset size 400), whereas the cases of at least a discordant subtype call are evaluated considering also the target label, i.e., the Ciriello *et al*. subtype.

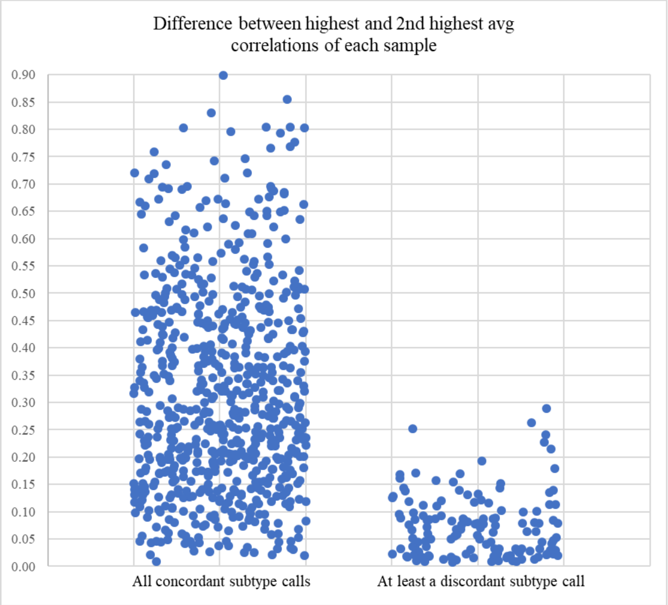

Figure S6. Discordant subtype calls in multiple PAM50 classifications mainly emerge in case of comparable average correlations with more than one subtype.

Figure S7 shows the distribution of subtypes when concordant calls are experienced within 10 PAM50 classifications and the Ciriello *et al*. subtypes, once the adopted strategy of reference building is fixed. Specifically, the concordant calls with the Ciriello *et al*. subtypes for the ten PAM50 classifications using as references the medians of different 60% ER+/40% ER-" subsets are reported in blue, whereas, the concordances of the PAM50 classifications using *AWCA* references are reported in orange.

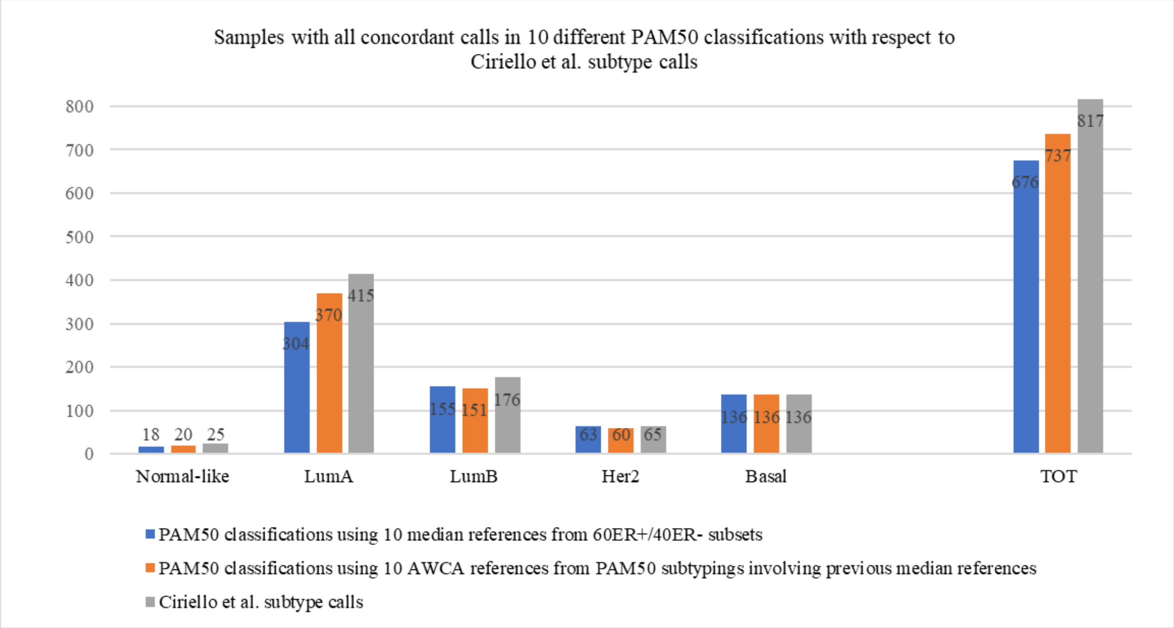

Figure S7. Distribution of subtypes within samples with all concordant calls, comparing different reference building strategies.

Finally, without considering the concordance with Ciriello *et al*. subtype calls, Figure S8 reports the maximum number of concordant calls within 10 PAM50 classifications, when the strategy of reference building is fixed.

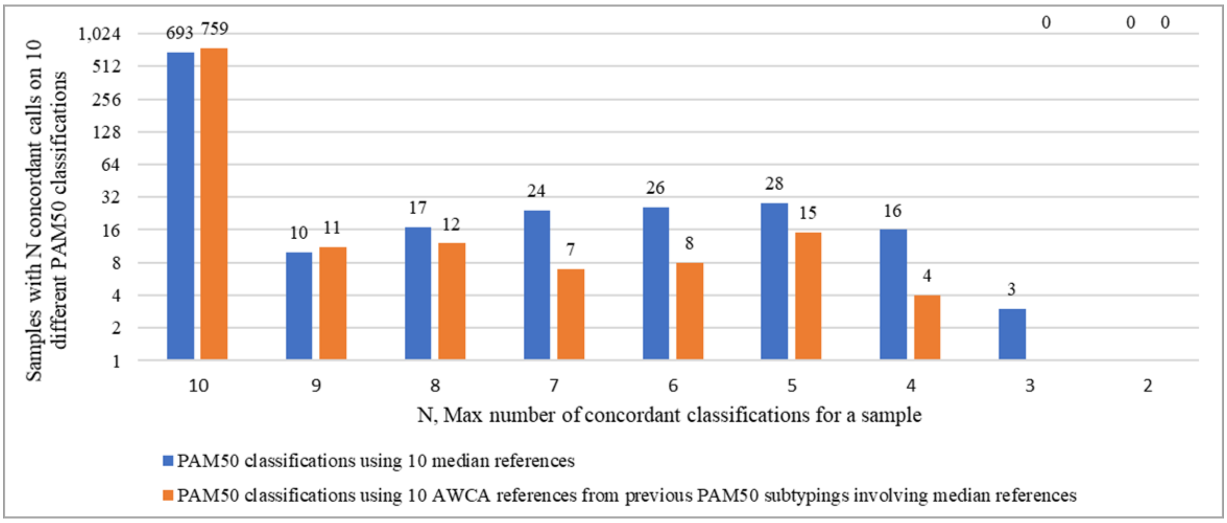

Figure S8. Amount of concordant calls in 10 PAM50 classifications, using two different strategies for reference computation.

In conclusion, discordant classifications involved mainly samples having comparable correlations with more subtypes and for some samples, the boundary between two or more classes appeared labile, maybe due to the possible coexistence of mixed traits.

This non-separability among subtypes also emerged clearly from the Principal Component Analysis that we performed independently for the RSEM values of the TCGA dataset and for the FPKM profiles of the GSE96058 dataset. As we can see from the graphs in Figure S9 showing the first two principal components, RSEM and FPKM data do not mix well. This compelled us to learn independently the parameters of the models dealing with RSEM or with FPKM data, as discussed later in the supervised learning context.

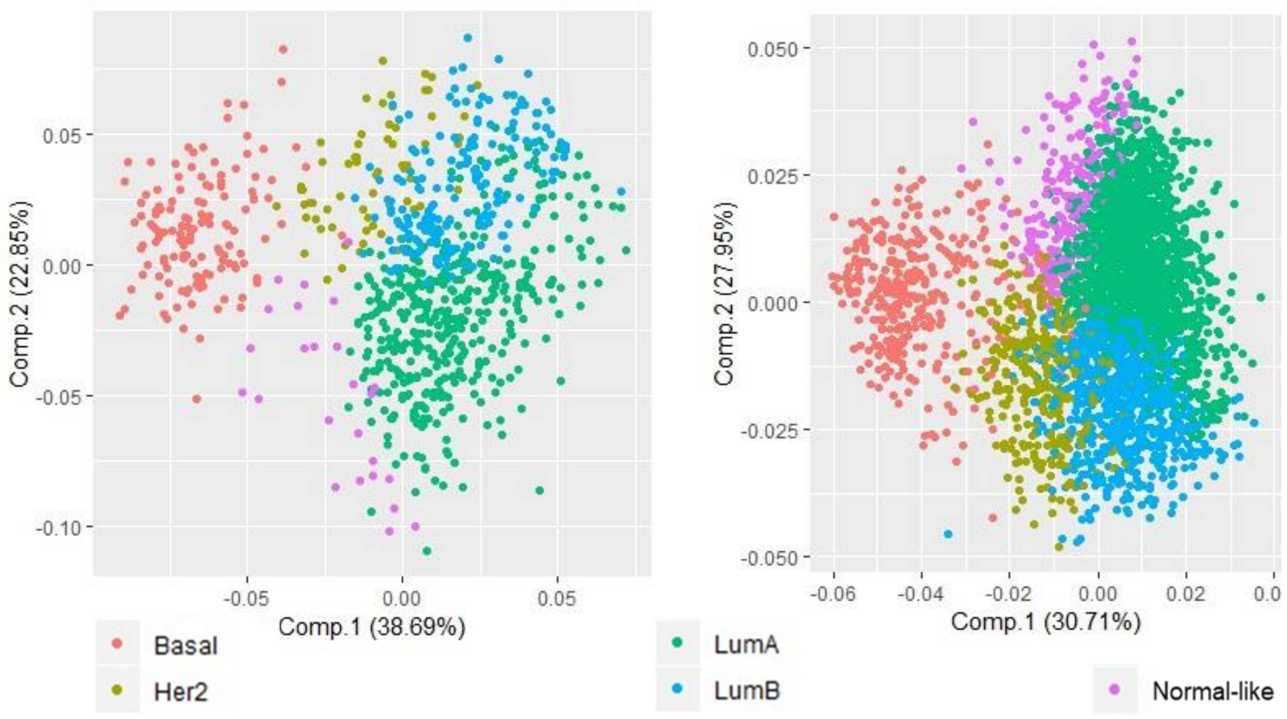

Figure S9. Principal component analyses of TCGA and GSE96058 datasets. First two components for each dataset under study.

S3. In-silico Prosigna emulation and Risk of Recurrence estimation

S3.1 Prosigna test and Risk of Recurrence models

The PAM50 assay was also converted into an alternative FDA approved predictive test called Prosigna (Wallden *et al.,* 2015), working on expression data profiled by NanoString nCounter® technology and implementing an alternative Nearest Shrunken Centroid classification (Tibshirani *et al*., 2002). The Prosigna test is used to define a category of metastatic risk at ten years in women undergoing surgery for invasive BC and focuses on a subset of the PAM50 panel called NANO46. Eventually, the test provides two distinct, but correlated, information: the BC intrinsic subtype and the category of risk of recurrence (low, medium or high). This latter one is precisely differentiated also based on lymph node involvement. The assay exploits a trademarked technology and a proprietary algorithm for breast cancer intrinsic subtyping, having the following peculiarities:

- The normalization procedures are specifically designed for expression data collected and processed on NanoString nCounter platforms
- The reference included in the Prosigna kit consists of in-vitro transcribed RNA-targets, to be processed together with the sample under study
- The classification predicted by Prosigna excludes the Normal-like class, which is instead present in other PAM50-based approaches
- The Nearest Shrunken Centroid approach uses Pearson linear correlation as distance metric to assign one of the four intrinsic subtypes.

Then, ROR score of a patient is computed from a weighted sum of NANO46 Pearson correlations to each intrinsic subtype centroid, proliferation score and tumor size parameters, according to the following model:

$$ROR_{Prosigna} = -0.0067\ Basal_{cor} + 0.4317\ Her2e_{cor} - 0.3172\ LumA_{cor} + 0.4894\ LumB_{cor} + 0.1981\ Proliferation_{score} + 0.1133\ Tumor_{size}$$

Prosigna predictive model was trained on NanoString profiles over nCounter® platform and its accuracy was confirmed also by subsequent analytical and clinical validation studies (Wallden et al., 2015). However, it is based on the former PAM50-based ROR-C predictor of Parker et al., a Cox regression model that estimates the risk of recurrence score (ROR-C) as a weighted sum of Spearman correlations with subtype centroids and tumor size parameter:

$$ROR - C = 0.05\ Basal_{cor} + 0.11\ Her2e_{cor} - 0.23\ LumA_{cor} + 0.09\ LumB_{cor} + 0.17\ Tumor_{size}$$

Following, we discuss our emulation on TCGA RNA-seq data of a Prosigna-inspired intrinsic subtyping and, eventually, we compare the so-obtained results with the ones found using the original PAM50 approach with the same *AWCA* reference.

S3.1.1 Data pre-processing and normalization

After discarding Normal-like samples and solving missing value issue as previously described, we focused not only on the PAM50 panel but also over eight housekeeping genes, needed to replicate Prosigna normalization steps. The Prosigna test requires first to divide each PAM50 sample profile by the geometric mean of the housekeeper absolute expression values. Since we worked on log2-space, we computed equivalently the arithmetic mean of the log2-values and we subtracted it from each log2-transformed sample profile. However, in our in-silico emulation on RNA-seq profiles, this normalization is a mere translation into the logarithmic space, which did not bring any advantage. A fixed value subtraction does not affect the Pearson correlation to be computed and not even the subsequent subtyping procedure. Furthermore, all the profiles were already UQ normalized within-sample. Housekeeper normalization is instead required for Prosigna test on the NanoString platform to correct input variability.

Following, we had to calculate the Log2ratios with respect to a reference sample. Although each real Prosigna assay run includes as reference the in vitro transcribed RNAs of all the 58 target genes, to be processed in the NanoString platform together with the sample, in our emulation we were forced to consider a fixed reference. Thus, we used an *AWCA*-based reference, built averaging in log2-space the gene expressions inside each subtype class and, then, taking the average of these within-class averages, to equate the contributions of the intrinsic subtypes despite their unbalanced proportions. But, in this case, the Normal-like class was a-priori excluded from the *AWCA* computation. Our reference sample was subtracted from the normalized profiles to

find the corresponding Log2ratios. This step is the most relevant for subsequent subtype calls since it provides differential alteration of each gene in each profile under study. Furthermore, in this case, we noticed that any sweep of the reference value would affect greatly the following subtype calls, even more than for the PAM50-method, showing the lack of robustness for a Prosigna inspired approach in absence of its in-vitro reference.

Lastly, we computed the z-scores from the Log2ratio values of each sample under study, as required by the Prosigna test to uncouple each gene from the sample mean and variance of a single realization. However, for our subtyping of RNA-seq profiles, this step appeared not meaningful, probably because each profile came from the same already normalized experimental cohort.

S3.1.2 Prosigna classification with the Nearest Shrunken Centroid method

Once all the normalization steps were concluded, we performed the subtyping procedure according to the Prosigna implementation of the Nearest Shrunken Centroid method. Test guidelines specify to calculate Pearson correlations between a sample z-score and each centroid of the four intrinsic subtypes. Prosigna centroids are anyhow disclosed only limited to the NANO46 gene list (a subset of the PAM50 panel). Thus, we faced an additional missing-value problem. At first, we replaced the four missing genes in the Prosigna centroids with the mean normalized expression of each of the 4 genes among all the 792 samples under study. Yet, this choice implied to cancel from the summation the contribution of the standard scores for each of the four genes of interest, as it can be easily seen from the following expression of the Pearson Correlation:

$$\varphi_{x,y} = \frac{1}{n-1}\sum_{i=1}^{n}\left(\frac{x_i-\bar{x}}{s_x}\right)\left(\frac{y_i-\bar{y}}{s_y}\right).$$

In this way, each Pearson coefficient was obtained from the division by n-1 with n equal to 50 because of the 50 genes of the PAM50 panel. Thus, the only difference with respect to completely discarding the gene expression of the four genes without centroid-values was to reduce the magnitude of the obtained correlations. Since Prosigna guidelines indicate NANO46 genes as the only ones significantly implied in clinical outcomes, we narrowed down our analysis over these genes only. For each sample we calculated the Pearson correlations between each z-score vector restricted to the NANO46 subset and all the disclosed Prosigna centroids, as required also for the subsequent computation of the ROR score. Then, each sample was assigned to the subtype for which it had the highest correlation coefficient.

S3.1.3 Prosigna classification of the TCGA dataset

To summarize, dealing with the TCGA dataset we discarded the 25 Normal-like specimens, whereas for the remaining 792 samples we performed the Prosigna subtyping using the average of within-class averages as reference. Thus, comparing our Prosigna-emulation subtyping results with the Ciriello *et al.* (2015) subtype calls (Figure S10), we found 111 not concordant assignments and overall accuracy of 0.86.

For Ciriello *et al*. (2015) Basal samples we had again a perfect match, whereas the most part of discordances was for Ciriello *et al*. (2015) Luminal A samples predicted as Luminal B. However, a misprediction from Luminal A to Luminal B, even whether should be effectively incorrect, would cause only a more pessimistic prediction, since Luminal B tumors lead to worst clinical outcomes than Luminal A ones. This pessimistic trend emerged again in 11 Ciriello *et al*. (2015) Her2-Enriched (Her2) samples predicted as Basal, known that Basal tumors usually follow the most aggressive clinical course.

	Classified as Basal	Classified as Her2	Classified as LumA	Classified as LumB	Recall	Recall %
Ciriello et al. Basal	**136**<br>100.0%				136/136	100.0%
Ciriello et al. Her2	**11**<br>16.9%	**49**<br>75.4%	**2**<br>3.1%	**3**<br>4.6%	49/65	75.4%
Ciriello et al. LumA		**2**<br><1%	**344**<br>82.9%	**69**<br>16.6%	344/415	82.9%
Ciriello et al. LumB	**2**<br>1.1%	**6**<br>3.4%	**16**<br>9.1%	**152**<br>86.4%	152/176	86.4%
Precision	136/149	49/57	344/362	152/224	**792**	
Precision %	91.3%	86.7%	95.0%	67.9%		

Figure S10. Confusion matrix of the Prosigna in silico emulation on the TCGA dataset.

S3.2 Conclusions from the Prosigna emulation and Risk of recurrence comparative analysis

Prosigna seemed an appealing candidate to work on RNA-seq data since it is designed for highly sensitive and reproducible NanoString profiles, able to quantify very low RNA concentrations similarly to what RNA-seq does. Nonetheless, we had to exclude Normal-like samples from our in-silico emulation, since the Prosigna test does not include the Normal-like class. After replicating all the normalization steps required by the Prosigna test, we performed the Prosigna implementation of the Nearest Shrunken Centroid subtyping method. However, it is relevant to stress that we were forced to use again the *AWCA* reference sample, computed within the dataset as the average of within-class averages (*AWCA*), whereas the real Prosigna test provides an in-vitro reference to be processed with the sample under study directly inside the proprietary platform. Eventually, comparing our Prosigna-emulation subtyping results with the published PAM50 subtype calls by Ciriello *et al*. (2015), we found an overall concordance of 86% and we noticed a slightly pessimistic prediction trend both with respect to the published subtype calls and to the predictions obtained with the PAM50 method using the same *AWCA* reference sample, as shown in Figure S11.

This kind of pessimistic trend could be caused by the reference choice or, partially, by how the Prosigna test and its centroids could have been designed, to handle false positive low-risk cases in subsequent clinical outcome predictions. Ultimately it is plausible that Prosigna normalization steps and in-vitro references are all probably intended to favour linearity between Prosigna centroids and each transformed profile under study, allowing the use of Pearson correlation as distance metric, but penalizing the portability of this approach on expression data different from the ones processed on its proprietary platform.

Eventually, we performed also a comparative analysis of the Risk of Recurrence (ROR) scores computed downstream of either the AWCA-based PAM50 classification, or the standard PAM50 technical replica (strictly emulating the published PAM50 classification), or the Prosigna subtyping. ROR scores of PAM50 and Prosigna assays were respectively obtained according to the predictive models presented in S3.1. Then, they were

tested against the 10-year overall survival annotations of the TCGA dataset to compare their prognostic ability. Prosigna ROR scores resulted the weakest in distinguishing good and poor long-term clinical outcomes.

Conversely, ROR scores from standard and AWCA-based PAM50 classifications were highly correlated to each other, with AWCA-based ROR scores that had the most statistically significant p-value in discriminating good and poor prognosis cases based on 10-year overall survival annotations.

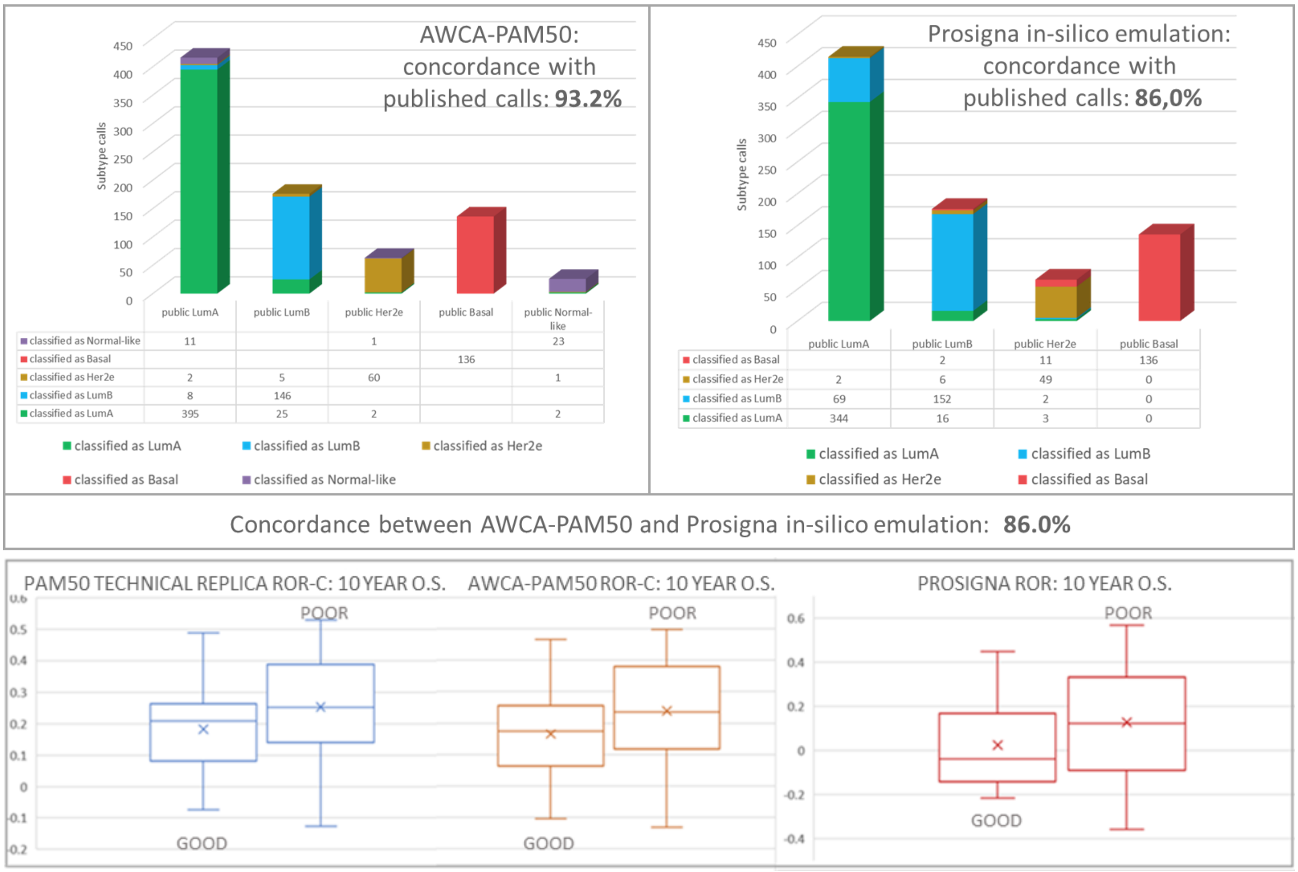

Figure S11. AWCA-PAM50 and Prosigna comparison. Both cases used an AWCA reference sample. Resulting subtype calls and concordances reached with published subtypes and with each other obtained from these intrinsic subtyping approaches (above). ROR scores distributions in 10-year overall survival (O.S.) good and poor prognosis cases for standard PAM50 technical replica, AWCA-based PAM50 and Prosigna.

In conclusion, the graphs in Figure S12 show clearly some remarkable differences between the centroid values of the original PAM50 subtypes (in blue) with respect to the Prosigna centroids (in orange). This comparison involves all the NANO46 genes, the subset of PAM50 genes for which Prosigna centroids were made available.

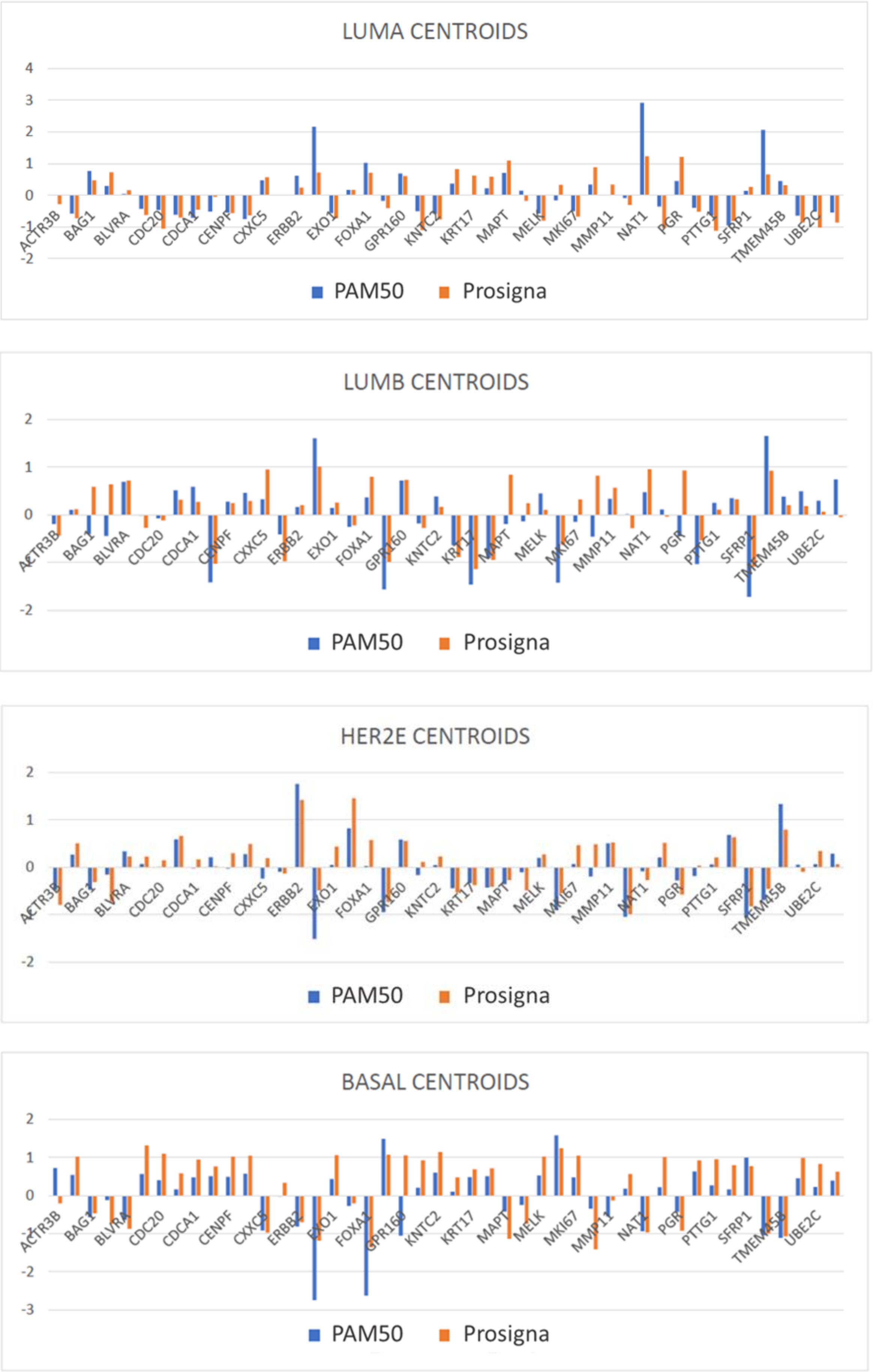

Figure S12. Original PAM50 centroids by Parker et al. (2009) vs. Prosigna test centroids for NanoString profiles.

Consequently, we went on with the original PAM50 method and its centroids, moving towards the analysis of the other datasets at our disposal, as described in the main paper. All the relevant results of this wide investigation are reported in Section S4, here below.

S4 PAM50 classifications of the GSE96058 dataset

Two PAM50 classifications of the GSE96058 dataset were performed using the average of within-class average (*AWCA*) sample references, alternatively including or excluding the Normal-like class, as previously done for the TCGA dataset. This pair of *AWCA* references were built within the GSE96058 dataset itself according to its published subtype calls, whose distribution is summarized in Figure S13. Both PAM50 classifications of the GSE96058 dataset confirmed the effectiveness of the *AWCA* references to replicate a PAM50 classification with high accuracy even in the absence of the exactly used reference, as shown in Figure S14.

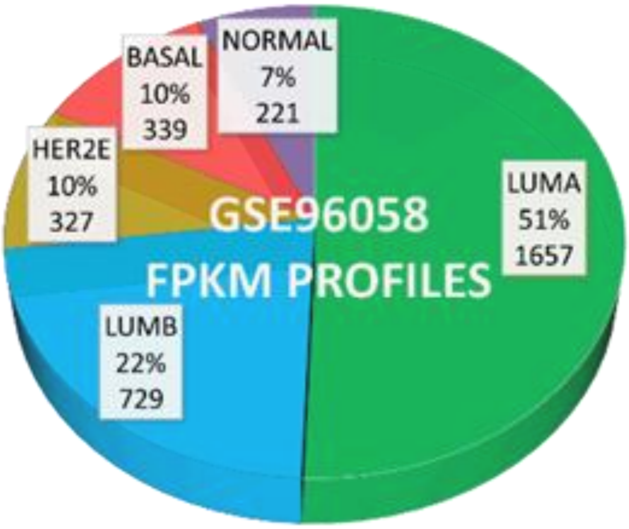

Figure S13. Distribution of the published subtypes for the GSE96058 dataset

REFERENCE: Average of within-class averages built A-POSTERIORI including Normal-like class

	Classified as Basal	Classified as Her2	Classified as LumA	Classified as LumB	Classified as Normal-like	Recall	Recall %
Disclosed Basal	**333** 98.2%	**2** 0.6%		**4** 1.2%		333/339	98.2%
Disclosed Her2		**287** 87.8%	**1** 0.3%	**39** 11.9%		287/327	87.8%
Disclosed LumA		**2** 0.1%	**1585** 95.7%	**70** 4.2%		1585/1657	95.7%
Disclosed LumB				**729** 100.0%		729/729	100.0%
Disclosed Normal-like	**4** 1.8%	**3** 1.4%	**34** 15.4%	**2** 0.9%	**178** 80.5%	178/221	80.5%
Precision	333/337	287/294	1585/1620	729/844	178/178	**3273**	
Precision %	98.8%	97.6%	97.8%	86.4%	100.0%		

PAM50 accuracy wrt published subtype calls: **95.1%**

REFERENCE: Average of within-class averages built A-POSTERIORI excluding Normal-like class

	Classified as Basal	Classified as Her2	Classified as LumA	Classified as LumB	Classified as Normal-like	Recall	Recall %
Disclosed Basal	**321** 94.7%		**3** 0.9%		**15** 4.4%	321/339	94.7%
Disclosed Her2	**7** 2.1%	**242** 74.0%	**34** 10.4%	**29** 8.9%	**15** 4.6%	242/327	74.0%
Disclosed LumA			**1652** 99.7%		**5** 0.3%	1652/1657	99.7%
Disclosed LumB	**1** >0.1%		**148** 20.3%	**580** 79.7%		580/729	79.7%
Disclosed Normal-like			**13** 5.9%		**208** 94.1%	208/221	94.1%
Precision	321/329	242/242	1652/1850	580/609	208/243	**3273**	
Precision %	97.6%	100.0%	89.3%	95.2%	85.6%		

PAM50 accuracy wrt published subtype calls: **91.8%**

Figure S14. PAM50 classifications of the GSE96058 dataset using AWCS references

S4.1 PAM50 classifications with internal or external *AWCA* references

The AWCA references built using the RSEM values of the TCGA dataset were then used for the subtyping of the RSEM PanCA dataset, and the AWCA references obtained from the FPKM values of the GSE96058 dataset for the subtyping of the FPKM GSE81538 profiles (Figure S15). The experienced accuracies were high, quite comparable both with the accuracies reached in classifying the TCGA and GSE96058 datasets (i.e., the datasets involved in the reference computations) and with the accuracies reached in the classifications of the PanCA and GSE81538 datasets themselves using their inner AWCA references. Notably, the high concordances found with the published subtype calls showed that it is possible to use even an external reference to center RNA-seq data for robust, single-sample PAM50 classification, provided that this external reference was built with RNA-seq data subject to the same normalization (RSEM or FPKM) of the data under PAM50-analysis. Furthermore, this hints at the chance of building and validate pre-defined reference samples able to assure future repeatability of classification.

Reference built as the average of within-class averages	A-POSTERIORI TESTING ON THE SAME DATASET USED FOR REFERENCE COMPUTATION	A-PRIORI TESTING ON ANOTHER DATASET WITH SAME NORMALIZATIONS
**TCGA dataset**	**PAM50 emulation on 817 TCGA RSEM profiles**	**PAM50 emulation on 236 PAN CANCER ATLAS RSEM profiles**
including Normal-like class	90.6%<br>77 DISCORDANCES	89.8% of accuracy<br>24 DISCORDANCES
excluding Normal-like class	93.2%<br>56 DISCORDANCES	96.6% of accuracy<br>8 DISCORDANCES
**GSE96058 dataset**	**PAM50 emulation on 3273 GSE96058 FPKM profiles**	**PAM50 emulation on 405 GSE81538 FPKM profiles**
including Normal-like class	95.1%<br>161 DISCORDANCES	96.3% of accuracy<br>15 DISCORDANCES
excluding Normal-like class	91.8%<br>270 DISCORDANCES	84.2% of accuracy<br>64 DISCORDANCES

Figure S15. PAM50 classifications of TCGA and PanCA datasets as well as of GSE96058 and GSE81538 datasets using average sample references (AWCA) built on TCGA and GSE96058 datasets, respectively.

The best performing *AWCA* references for RSEM and FPKM data (built on TCGA excluding Normal-like class or in GSE96058, including Normal-like samples), are provided in Table S3 here below.

Table S3. Best performing AWCA references for RSEM or FPKM data

PAM50 GENES	RSEM	FPKM	PAM50 GENES	RSEM	FPKM
ACTR3B	7.8100	1.4074	***KNTC2***	8.3203	1.4998
ANLN	9.9353	2.1223	***KRT14***	7.8568	4.3271
BAG1	10.4049	4.2097	***KRT17***	8.8621	4.8571
BCL2	9.6031	3.1651	***KRT5***	8.7922	4.4478
BIRC5	8.7554	2.8230	***MAPT***	9.0991	2.6669
BLVRA	10.1555	5.5727	***MDM2***	10.9720	3.2181
CCNB1	10.1547	3.7837	***MELK***	8.7175	1.8623
CCNE1	6.9164	1.0039	***MIA***	4.3840	2.2149
CDC20	9.5420	3.5678	***MKI67***	11.1800	1.9167
CDC6	9.0988	1.9639	***MLPH***	11.1385	4.8013
CDCA1	8.3907	1.4540	***MMP11***	12.3251	6.8134
CDH3	9.7252	3.0283	***MYBL2***	10.0170	2.8490
CENPF	11.2564	2.2384	***MYC***	10.4113	5.1635
CEP55	8.9771	1.9884	***NAT1***	8.8031	3.6197
CXXC5	9.1479	4.9090	***ORC6L***	7.2664	1.4998
EGFR	7.6419	2.1281	***PGR***	7.6944	1.4540
ERBB2	13.3007	6.1550	***PHGDH***	10.1339	0.9210
ESR1	10.4286	4.5612	***PTTG1***	8.8261	0.7854
EXO1	8.1784	1.0373	***RRM2***	10.2831	4.2061
FGFR4	7.4098	1.2902	***SFRP1***	9.2263	3.8384
FOXA1	11.1919	5.3494	***SLC39A6***	13.0777	3.2620
FOXC1	7.6145	2.0805	***TMEM45B***	6.6985	3.9320
GPR160	9.5055	3.4496	***TYMS***	9.4346	6.7095
GRB7	9.6301	3.7535	***UBE2C***	9.5297	1.5739
KIF2C	9.1794	2.0186	***UBE2T***	8.9184	2.4290

Notably, at https://github.com/DEIB-GECO/BC_Intrinsic_subtyping, we provide the R code both to build AWCA references and to perform single-sample PAM50 classifications of RNA-seq profiles using our pre-computed AWCA references (for RSEM or FPKM expression data). For single-sample AWCA-based PAM50 subtyping we strongly encourage to use the AWCA reference obtained for the same data normalization of the RNA-seq data under exam. Conversely, the R code to generate AWCA references can be used on any expression data, even from other technical platforms (see the Appendix for further details on the evaluation of gene expression data from Affymetrix GeneChips). However, to provide valuable AWCA references to be further used for single-sample classification of independent expression profiles, we suggest to check the original dataset composition and evaluate if its ER/PR/HER status distributions are representative of BC disease, according to the references in the literature.

S5 Machine learning path

S5.1 Machine learning survey and embedded regularization

In order to perform breast cancer (BC) intrinsic subtyping, different supervised methods were taken into account and compared. As always in machine learning problems, the "No free lunch theorem" suggested to investigate multiple classification models up to find a learner whose generalization accuracy, tested over unseen samples, appeared to suit properly our specific task. Additionally, we tried alternative feature selection approaches to find a set of genes relevant with respect to our subtyping task, and that could be used as feature space to improve the performances of the machine learning model under evaluation. In this perspective, most of the assessed learners were trained with embedded regularizations.

Embedded regularization methods are often used to learn which features best contribute to the accuracy of the model while the model is being fitted. These canonical feature selection methods introduce additional penalization constraints into the optimization function of a predictive algorithm as to shrink some parameters towards zero, or even push the model toward lower complexity by driving some parameters to zero. Consequently, they reduce variance and risk of overfitting. Practically, if we consider a specific task to be accomplished through a model having w as parameter vector and we take as optimization function to be minimized a suitable loss function $L_D(w)$, we can add a regularization term $L_W(w)$ to control overfitting, such that the total loss function to be minimized takes the form:

$$L(w) = L_D\,(w)\; + \gamma\, L_W\,(w)$$

where γ is the regularization hyperparameter in charge of weighing the whole penalization term. Since γ controls the strength of shrinkage and feature selection, it must be properly tuned.

In case of weight decay (or Ridge, L2-regularization), the penalization term is:

$$L_W(w) = \gamma w^T w = \gamma \|w\|_2^2$$

It provides a parameter shrinkage by reducing the overall squared Euclidean norm of parameters and, as suggested by the name, in a sequential learning algorithm it encourages parameter values (weights) to decay toward zero. Nonetheless, none parameter and consequently none feature is effectively annulled.

In case of Lasso (or L1-regularization), the penalization term is:

$$L_W(w) = \gamma \|w\|_1$$

where $\|w\|_1$is simply the sum of the absolute values of all the model parameters. This regularization, for γ sufficiently large, drives to zero some parameters leading to a sparse model in which the corresponding variables play no role. Nonetheless, Lasso fails to do grouped selection, tending to select only one variable from a group and ignoring the others if correlated. This can compromise robustness and stability in case of high variability of features. Furthermore, when the number of parameters is bigger than the number of samples, Lasso regularization selects at most n features, where n is equal to the number of samples. Thus, in a genomic dataset, the number of selected genes is bounded by the number of samples, introducing additional bias.

Finally, in case of elastic net (or combined L1-L2-regularization), the penalization term is:

$$L_W(w) = \gamma_{L1} \|w\|_1 + \gamma_{L2} \|w\|_2^2$$

where γ_{L1} and γ_{L2} are the Lasso and Ridge regularization hyperparameters, respectively. This combined regularization overcomes the flaws of the previous ones since the L1-part of the penalty generates a sparse model with effective feature selection, whereas the quadratic L2-part removes the limitation on the number of selected features, encourages grouping effect and stabilizes the L1-regularization path.

S5.2 Classifiers under evaluation

The following subsections summarize the characteristics of the methods we implemented and tested.

S5.2.1 Ensemble methods: Multiclass Decision Forest and Decision Jungle

Ensemble models often provide better coverage and accuracy than a single classifier over complex tasks, combining several learners to manage and improve bias-variance trade-off. In our Decision Forest algorithm, bagging is implemented to train each decision tree in the ensemble using a randomly drawn subset of the training set. Furthermore, each decision tree restricts to a fixed number the features randomly selected for splitting each node. This kind of approach, where the random selection of features is combined with bagging, is known also as "Random forest" from the trademark algorithm of Leo Breiman and Adele Cutler. However, we referred to this approach as multiclass Decision Forest with bagging. The algorithm works by building a classification decision forest with multiple decision trees, such that each tree outputs a non-normalized frequency histogram of labels, and then the most popular output class is obtained by voting. Voting aggregation process sums these histograms and normalizes the result to get the "probability" for each label. The trees that have high prediction confidence have a greater weight in the final decision of the ensemble to achieve higher classification accuracy. Decision trees can represent non-linear decision boundaries and are non-parametric models, thus the sample population is not required to fit any parametrized distribution. Furthermore, they are efficient in computation and memory usage and perform integrated feature selection that makes them resilient in the presence of noisy features. All these peculiarities are shared with Decision Jungle, used as an alternative bagging-based ensemble method. Unlike conventional decision trees that only allow one path to every node, Decision Jungles are compact and powerful discriminative models for classification, where directed acyclic graphs (DAGs) allow multiple paths from the root to each leaf. Specifically, decision jungles use node merging as well as node splitting algorithms to jointly optimize both the features and the structure of the DAGs efficiently, while the weighted sum of entropies at the leaves is minimized during training. In the end, compared to decision forests, decision jungles require dramatically less memory while, usually, considerably improving generalization.

S5.2.2 Multiclass Logistic Regression

Logistic Regression is a classification method that uses the logistic sigmoid function on a linear combination of the features ϕ, weighted by a parameter vector w, to estimate the posterior probability of a sample to belonging to a class C: $p(C|\phi) = \frac{1}{1+\exp(-w^T\phi)} = \sigma(w^T\phi)$

This simple approach is thought for binary classification, where the alternative class probability is just the complement of the found probability. In the multiclass version, instead, multiclass Logistic Regression is also known as softmax Logistic Regression, since the posterior probabilities are given by a softmax transformation of the activation functions: $p(C_k|\phi) = y_k(\phi) = \frac{\exp(w_k^T\phi)}{\Sigma_j \exp\left(w_j^T\phi\right)}$

where each activation $a_k = w_k^T\phi$ is a linear combination of the features ϕ, weighted by a parameter vector w_k, specific for each class C_k. Softmax transformation squeezes toward one the biggest exponential, while pushes toward zero smallest ones. Finally, the predicted class is the one with the highest probability. For an N-dimensional feature space, this model has N adjustable parameters for each class C_k, to be computed through maximum likelihood estimation up to minimizing the following cross-entropy error function for the multivariate case: $L_D(w_1 \dots w_k) = -\log p(T|\phi; w_1 \dots w_k) = \sum_{n=1}^{N}\left(\sum_{k=1}^{K} -t_{nk}\log(y_{nk})\right)$

where y_{nk} is the probability that the n-th training sample belongs to the C_k class, according to the logistic function estimate; T is, instead, the matrix of the true labels, such that t_{nk} is usually null for each class except the C_k class the n-th training sample belongs to, and whose value is one. We used this method with a combined L1-L2 regularization (elastic net) and therefore, to train the model, the following additional penalization term was added to the reported loss function.

$$L_W(w) = \gamma_{L1}\|w\|_1 + \gamma_{L2}\|w\|_2^2$$

S5.2.3 Multiclass Neural Network

A Feed-Forward artificial Neural Network (FFNN) is a non-linear model characterized by the number of neurons (nodes), their topology, their activation functions and the values of synaptic weights and biases. It includes a set of interconnected layers. The inputs are the first layer and are connected to an output layer by an acyclic graph comprised of weighted edges and nodes, i.e., neurons. Multiple hidden layers can be inserted between the input and output layers, but theoretically, having input from a compact set, just a single hidden layer and enough non-linear activation functions could model almost every non-linear function. In fact, a FFNN provides a linear combination of non-linear activation functions for each neuron along the feed-forward path, since all nodes in a layer are connected by the weighted edges to nodes in the next layer (Figure S16). Let consider i inputs, j hidden neurons (on a single hidden layer) and k outputs, with H as activation function for the hidden neurons and G as activation function for the output neurons. Let $w_{11} \ldots w_{ji}$ be the input-hidden layer weights; whereas, let $W_{k1} \ldots W_{kj}$ be the hidden-output layer weights.

The generic output O_k will be: $O_k = G_k\left(\sum_{j'=1}^{j} W_{kj'} H_j\left(\sum_{i'=1}^{i} w_{j'i'} I_{i'}\right)\right)$

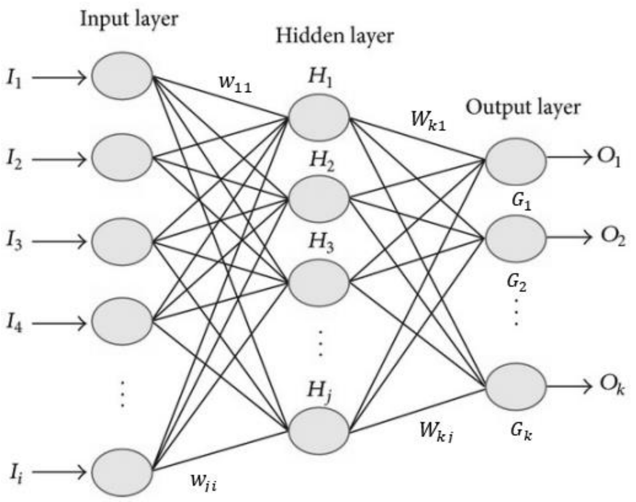

Figure S16. Multi-output Feed-Forward Neural Network.

Most predictive tasks can be accomplished easily with at most two hidden layers, whereas deep neural networks with many layers can be very effective in complex tasks, including automated feature extraction steps. However, in our case, we decided to evaluate a fully connected Feed-Forward Neural Network with a single hidden layer and sigmoid activation functions, both for the hidden and output layers. Each output neuron is associated with a single class to evaluate the probability of belonging to that class; then, the class with the highest probability is the predicted one for the classification task. The relationship between inputs and outputs is learned from training the FFNN on normalized input data. The cross-entropy error function is adopted as loss function $L(W)$ with weight decay (L2-regularization), and backpropagation with learning rate η and momentum α is used as rule to learn iteratively the parameters of the model, i.e., the weights associated with each edge of the network. In particular, the momentum term is added to avoid oscillation and risk of local minima. This means for a generic parameter w that its next value at time k+1 is:

$$w^{k+1} = w^k - \eta \left.\frac{\partial L(w)}{\partial w}\right|_k + \alpha \left.\frac{\partial L_D(w)}{\partial w}\right|_{k-1}$$

Cross-validation is employed as tuning technique for model selection, up to find a proper number of hidden neurons, as well as suitable L2-regularization hyperparameter, learning rate η , and momentum α.

S5.2.4 One-vs-All multiclass Support Vector Machine

Support Vector Machines (SVMs) have been extensively used in the classification of gene expression data with thousands of features and limited set of samples, even if they are originally designed for binary classification tasks. In fact, the basic concept behind SVM binary classification for linearly separable data is finding among the infinite linear boundaries (called hyperplanes) that can separate the data, the optimal hyperplane that maximizes the margin from the nearest points of each class. Nevertheless, not every dataset is linearly separable, and SVMs are able to classify also complex and nonlinear data by taking advantage of the "Kernel Trick" to move toward higher dimensional spaces. Specifically, an SVM classifier can use a kernel function K to nonlinearly transform the data into a higher dimension in which the problem is reduced to the linear case. Commonly used kernel functions include Polynomial, Gaussian radial basis function (RBF), or sigmoid functions. Hence, when target values t are in {-1,1} and S is the set of indexes of the support vectors x_m with their associated parameters a_m, the class prediction for an unseen sample x_q is computed as:

$$f(x_q) = sgn\left(\sum_{m\in S} \alpha_m t_m K(x_q, x_m) + b\right)$$

where $\sum_{m\in S} \alpha_m t_m K(x_q, x_m) + b = 0$ is the equation of the hyperplane able to separate the data accurately. However, selecting the kernel function alongside the parameterization could be challenging during the model selection phase. Furthermore, handling noisy data requires to define a non-perfectly separating hyperplane that anyway minimizes the classification error, without increasing model complexity and introducing the risk of overfitting. Eventually, SVM can be extended to deal with multi-class classification problems as we did, i.e., using one SVM for each class in combination with an approach called One-vs-All. For each SVM, a given class is fitted against the rest of the classes, all combined together. Then, a prediction is performed by running all these binary classifiers and choosing the prediction with the highest confidence score. In our implementation, each SVM includes also Lasso regularization, to reduce the risk of overfitting, performing embedded feature selection.

S5.3 Training phase and classifier survey

For the training phase, the TCGA training set was split into 10 folds, randomly drawn and balanced with respect to subtypes. Given the generic classifier under study, each specific model, tuned with a specific combination of hyperparameters, was trained with 10-fold cross-validation, i.e., 10 times and each time over a different set of 9 folds, leaving the 10th fold out as validation set. Thus, each already tuned specific model learned its optimal parameter values over 10 slightly different training sets and could estimate its generalization accuracy, each time over a bunch of samples extraneous with respect to that turn of training. We repeated this training phase for each family of classifiers under study and we found the best-trained models, i.e., the models whose hyperparameter setting and learned parameter values led to the best generalization accuracy, estimated through cross-validation. Further details about the machine learning approaches under study are summarized in Table S4, whereas Figures S17-S21 report the performances on the TCGA test set of each assessed best-trained model from each family of classifiers.

Table S4. Machine learning survey. Accuracies estimated with 10-fold cross-validation on TCGA data, and details about the best trained models for each family of classifiers.

	Logistic Regression - multiclass	Decision Jungle (bagging) - multiclass	Decision Forest (bagging) - multiclass	Feed Forward Neural Network - multiclass	SVM (one-vs-all)
GLOBAL ACCURACY estimated with 10-folds cross-validation					
Mean	0.8398	0.6421	0.4737	0.7462	0.8042
Median	0.8364	0.6545	0.4955	0.8409	0.8136
Min	0.8136	0.3727	0.2273	0.2227	0.7727
Max	0.8818	0.8591	0.8455	0.8636	0.8364
Standard Deviation	0.0164	0.1364	0.2188	0.2294	0.0232
C.I. 90%	0.00366	0.02494	0.04000	0.07496	0.00986
C.I. 95%	0.00436	0.02971	0.04766	0.08932	0.01174
BEST TRAINED MODEL	L1 Weight 1<br>L2 Weight 1<br>Optimization Tolerance 0.0001<br>Memory Size 20	Optimization Step Count 16384<br>Max Width 512<br>Max Depth 32<br>Ensemble Element Count 32	Decision trees 32<br>Min # samples per leaf node 4<br>Random splits per node 1024<br>Max depth of decision trees 64	Initial Weights 0.1<br>Learning Rate 0.01<br>Loss Cross Entropy<br>Momentum 0<br>Data Normalizer Type MinMax<br>Shuffle Yes	Number of Iterations 100<br>Lambda 0.001<br>Normalize Features True<br>Perform Projection False

Multiclass Logistic Regression

Metrics on TCGA test set

Overall accuracy	0.85256
Micro-averaged precision	0.85256
Macro-averaged precision	0.66175
Micro-averaged recall	0.85256
Macro-averaged recall	0.81436

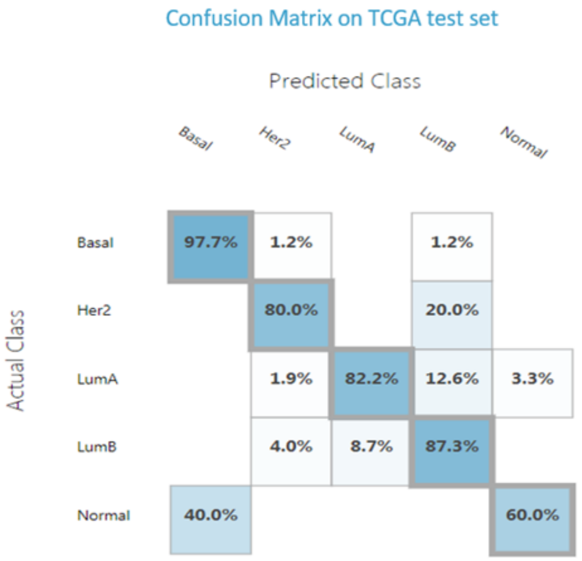

Figure S17. Multiclass Logistic Regression: Performances and confusion matrix on TCGA test set for the model trained with cross-validation on TCGA training set

Multiclass Decision Jungle (bagging)

Metrics on TCGA test set

Overall accuracy	0.83751
Micro-averaged precision	0.83751
Macro-averaged precision	0.63652
Micro-averaged recall	0.83751
Macro-averaged recall	0.74574

Confusion Matrix on TCGA test set

Actual Class \ Predicted Class	Basal	Her2	LumA	LumB	Normal
Basal	97.7%	1.2%		1.2%	
Her2		73.3%		26.7%	
LumA		1.1%	83.3%	10.4%	5.2%
LumB		4.8%	16.7%	78.6%	
Normal	40.0%	20.0%			40.0%

Figure S18. Multiclass Decision Jungle: Performances and confusion matrix on TCGA test set for the model trained with cross-validation on TCGA training set

Multiclass Decision Forest (bagging)

Metrics on TCGA test set

Overall accuracy	0.82916
Micro-averaged precision	0.82916
Macro-averaged precision	0.63841
Micro-averaged recall	0.82916
Macro-averaged recall	0.76964

Confusion Matrix on TCGA test set

Actual Class \ Predicted Class	Basal	Her2	LumA	LumB	Normal
Basal	97.7%	1.2%		1.2%	
Her2		86.7%		13.3%	
LumA		1.4%	81.1%	13.7%	3.8%
LumB		4.8%	15.9%	79.4%	
Normal	40.0%	20.0%			40.0%

Figure S19. Multiclass Decision Forest: Performances and confusion matrix on TCGA test set for the model trained with cross-validation on TCGA training set

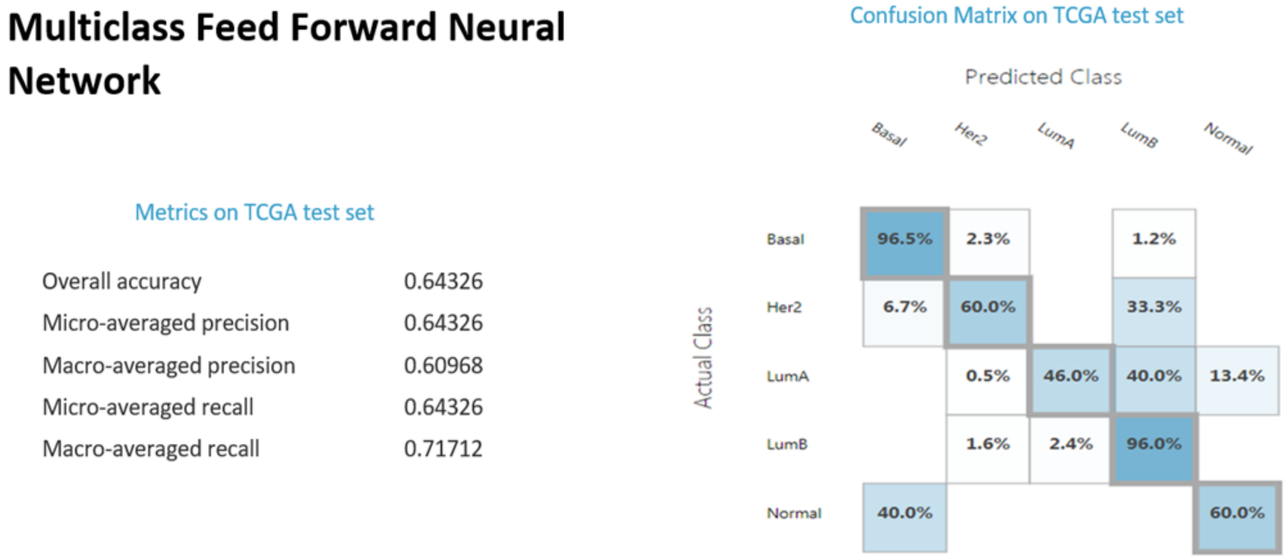

Figure S20. Multiclass Feed Forward Neural Network: Performances and confusion matrix on TCGA test set for the model trained with cross-validation on TCGA training set

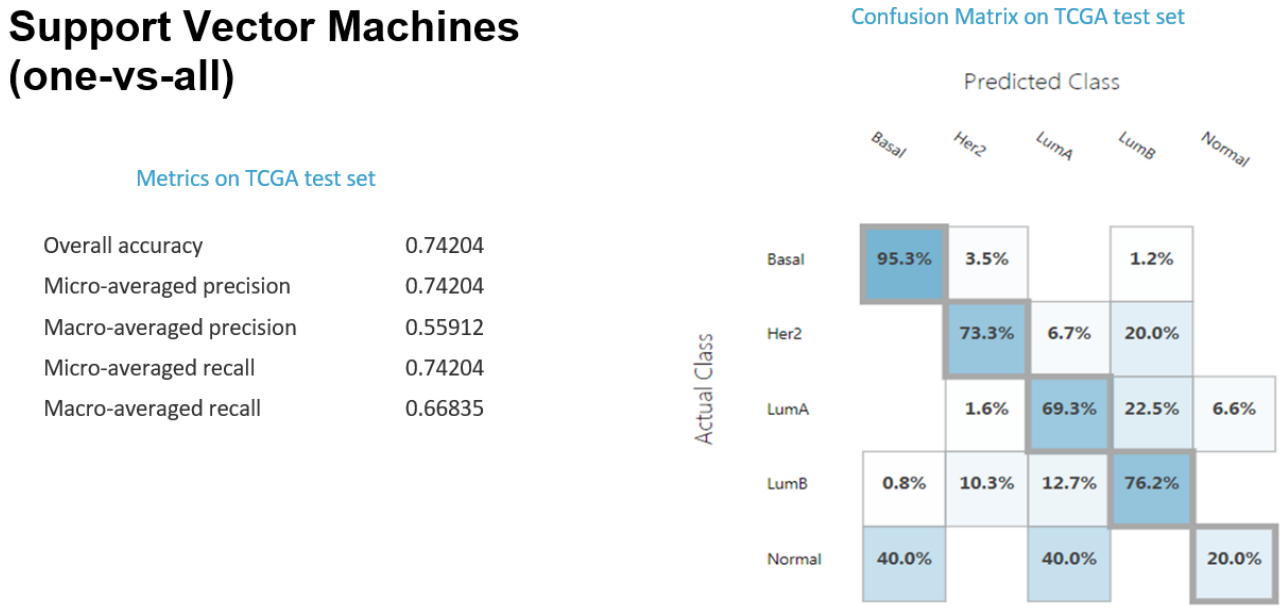

Figure S21. Support Vector Machines (one-VS-all): Performances and confusion matrix on TCGA test set for the model trained with cross-validation on TCGA training set

S5.4 Feature selection strategies

Starting from the most promising supervised method, i.e., regularized multiclass Logistic Regression, several additional feature selection strategies were assessed, other than simply considering the PAM50 genes already involved in PAM50-based subtyping. The aim was to reduce the feature space of interest compared to the whole set of profiled genes and possibly improve the performance of the learner in performing the intrinsic subtyping task. First, these approaches were applied to the RSEM profiles of the TCGA dataset, for both training and testing. Then, some relevant feature spaces were employed also to train and test the multiclass Logistic Regression on the FPKM data of the GSE96058 dataset. Eventually, both PanCA and GSE81538 datasets were used as external datasets for testing the corresponding models trained respectively on the RSEM profiles of the TCGA dataset or on the FPKM profiles of the GSE96058 dataset. Further details about all the assessed feature selection methods are briefly discussed in the following subsections.

S5.4.1 Blind approach

At first, we tried a typical approach of feature selection for genomic data that works completely blindly with respect to the predictive task and depends only on the magnitudes and dynamicity of the collected expression values. This feature selection approach excluded simply the lowest expressed genes and the genes with the lowest variability, since they often represented less reproducible or potentially less useful features when working across multiple sequencing experiments. Therefore, specifically, we reduced the initial dimensionality of the TCGA dataset by discarding:

- Genes whose average expression in 817 samples was in the 1st quartile of the distribution of the mean expressions of all the sequenced genes (exclusion of low-expressing genes)
- Genes whose standard deviation, compared to the mean value of the expressions of the gene in the dataset, fell within the 1st quartile of the standard deviations of all the sequenced genes (exclusion of genes with low variability).

After this feature selection step, the genes for each sample were almost halved, and hence the training set had 10,606 features (instead of 19,737). But after tuning and training (done exactly as for previous analysis) a regularized multiclass Logistic Regression over this reduced training set, the cross-validated accuracy was lower than the previous estimate. Then, we assessed that also over the test set the new best-trained model behaved worst, with an accuracy around 0.81 with respect to the accuracy of 0.85 experienced for the best model trained on the entire original feature space. Hence, this choice led to disappointing results. Indeed, the high sensitivity and accuracy of the RNA-sequencing quantitative measurements could make relevant for our subtyping task also low-expressed genes, excluded from classification methods based on the expertise gained on not up-to-date sequencing technologies.

S5.4.2 Filter based approaches

Among the assessed external strategies, not involving Logistic Regression during the feature selection phase, we considered several filter-based methods, since they are effective in computation time and robust to overfitting. Filter-based methods use a statistical measure to assign scores to features, which are then ranked accordingly and either selected to be kept or removed from the feature space. In supervised tasks, each feature is evaluated with respect to the target to be predicted, but regardless of the chosen model and without considering relationships among features, with some redundancy risk. However, we filtered out a certain number of features supposed to be less meaningful, or even irrelevant, for our subtyping task according to the next scoring metrics: 1) Fisher scores; 2) Mutual Information; 3) Chi-squared scores; 4) Spearman Correlation. In detail, we proceeded with the following workflow for each scoring metric. We started from the complete *TCGA* training set and we collected 5 wide random samplings with replacement, stratified with respect to subtypes. For each sampling, we used the given metric to score all the 19,737 original genes independently from the others, known that the score of each gene estimates its relationship with the Ciriello *et al.* (2015) target subtype to be predicted. Hence, for each scoring metric we obtained 5 rankings (one for each sampling) and, consequently, 5 scores for each gene with the given metric. The next step was simply to sum, in a single overall gene score, the 5 scores of each gene within a given metric and then to make the overall ranking for

that given metric. Notice that the overall ranking of each scoring metric is independent of the others. Using an overall ranking improved the robustness of the genes that emerged as the most relevant ones for a given metric since first positions include only genes meaningful across all the 5 cases scored with that given metric. Following, we used as alternative reduced feature spaces the top 1000 genes of each overall ranking (i.e., scoring metric), since the non-null weights (then non-null features) learned by the regularized multiclass Logistic Regression trained on the complete *TCGA* training set of 19,737 features/genes were approximately one thousand.

S5.4.3 DEG-based approach: limma analysis

We analyzed the whole original feature space of 19,737 genes, using limma, an R package for the analysis of gene expression data (from microarray or RNA-seq), whose core capability is the use of linear models to assess differential expression in the context of multifactor designed experiments. Specifically, by setting only the Ciriello *et al*. (2015) target subtype as variable in the experimental design, we noticed that the Basal class dominates because its expression profiles are more easily recognizable and different from those of the other classes, even more than the profiles of the other subtypes are different from each others. Thus, using the F statistic to rank the genes would favor mainly the distinction of Basal class from the other subtypes. To overcome this issue, we defined all the 10 possible contrasts between the 5 Breast Cancer subtypes, making pairwise differential analyses. Then, fixed an integer N, we selected genes according to the following criterion: for each contrast (i.e., for each pair of subtypes) given M genes differentially expressed according to the Sequencing Quality Control Consortium (SEQC) criterion (SEQC Consortium, 2014) (i.e., | log(Fold Change) | > 1 and p-value < 0.01), the top N genes according to the p-value were chosen if M>N, otherwise all M differentially expressed genes were selected. In this way, we obtained 10 lists, each one including the top N genes (or at least all the M genes) differentially expressed in a given pairwise contrast. For each considered choice of N, through the union of the corresponding 10 lists, we gained a complete set of genes to be selected as feature space to train our regularized logistic regression. This criterion guaranteed that each possible so-called limma*N* feature space certainly includes the first N (or all the M) genes emerged as differentially expressed genes for each contrast, and therefore relevant to distinguish at least a couple of subtypes. Notice that as previously we tuned and trained our regularized Logistic Regression on the alternative feature spaces obtained for 11 different N values, ranging from 10 to 1000 (i.e., 10, 30, 50, 60, 70, 80, 90, 100, 200, 500, 1000).

S5.4.4 Feature selection strategy with a wrapper method

As the last step of our study, we assessed if the prediction performances of the regularized multiclass Logistic Regression could be further improved using additionally a wrapper method. Wrapper methods consider feature selection as a search problem where different combinations of features are evaluated and compared based on the performances of the chosen model, detecting also possible feature interactions to avoid redundancy. However, the main limitation of these methods is the high computational cost, both in terms of time and memory, that made their use computationally unaffordable in case of wide feature space dimensionalities, like the ones of the RNA-seq datasets. Therefore, we implemented a strategy involving a wrapper method, but alternatively using as starting feature space some promising reduced feature spaces, like the ones previously obtained from Chi-squared based or DEG-based filtering. Sequential backward elimination was chosen as heuristic method to find reduced subsets of relevant features, since it appeared to us less prone to underfitting with respect to a forward selection of features, considering what we experienced with multiple assessments of the same task performed over smaller feature spaces of proof. The backward elimination algorithm started estimating the performances of the learner trained over the N-dimensionality whole-features training set. Then, it considers and estimates the accuracy of all the subsets with N-1 features. After choosing the subset whose estimated accuracy is the highest, it excludes accordingly the feature not belonging to that subset. In the case of equality, the elimination choice is sequential. Thus, the method iteratively discards one gene at a time until no more feature elimination improves the accuracy of the regularized multiclass Logistic Regression beyond a fixed threshold of gain. In our study, first, we applied our wrapper method over the top 1000 Chi-squared based genes; although multiple runs were unfeasible for

computational reasons, this still raw approach, combining regularized logistic regression, filter-based feature selection and the additional backward elimination, appeared worthy of further investigations, with a generalization accuracy estimated with 5-folds cross-validation of about 0.95. Therefore, also considering that the number of genes preserved by this first trial was about one hundred, we chose as starting feature spaces for other attempts of the combined strategy the top 200 and top 500 Chi-squared-based genes and our limma50 signature. For each alternative starting feature space, we carried out in parallel ten independent runs of backward elimination, performing each run with randomized feature order as to mitigate the bias introduced by the sequential gene scrolling (solvable only through an unfeasible exhaustive search). Since kept genes in each run were not robust (also because of the needed feature shuffling), eventually we combined all the genes kept in at least one run, with a sort of downstream preservation strategy, to face the lack of robustness experienced from multiple runs, while trying to increase the subtyping capability of the collected subsets. Each gene signature preserved from the three corresponding starting feature spaces was used in its turn as a reduced feature space.

Implementation of the wrapper method

To implement the strategy involving a wrapper method, we could not customize the Azure Machine Learning Studio workflow with R-scripts, due to the complexity of the investigation. Therefore, we run on a server the R code needed to implement the wrapper method, extracting data of interest through the *AzureML* library for R. Furthermore, we took advantage of several functions provided by *LiblineaR*, a package for the estimation of predictive regularized linear models for classification and regression, and by *mlr*, a complete framework for machine learning experiments in R. We used the function *makeFeatSelWrapper* from the *mlr* library, providing as parameters: 1) a regularized multiclass Logistic Regression as learner for the subtyping task (defined using *LiblineaR* library functions); 2) 5-fold cross-validation as approach for the evaluation of the performances over investigated subsets of features; 3) the overall accuracy as the measure for performance assessment; and 4) the feature selection strategy. This latter one was specified through the *makeFeatSelControlSequential* function together with its parameter indicating the choice of a backward elimination strategy. According to backward elimination, the feature selection algorithm starts estimating the performances of our learner trained over the whole-feature training set, i.e., the top N genes, and iteratively discards one gene at a time, provided that our learner, trained over a subset excluding that gene, shows higher estimated accuracy.

S6 Performances of the main Logistic Regression models under evaluation

To ease the access and comprehension of the relevant collected results, all the confusion matrices concerning different regularized multiclass Logistic Regression (mLR) models trained on the TCGA training set, or on the GSE96058 training set, and evaluated on the available inner and external test sets (PanCa and GSE81538) are reported here below in Figures S22-S36, together with some other relevant plots. Eventually, Tables S5 and S6 summarize the performances reached in cross-validation and internal testing, with mLRs using the most relevant emerged signatures as feature spaces and meant respectively for RSEM or FPKM data.

Notice that for each confusion matrix it is clearly specified: the training set on which the model was trained, the feature space of interest, for which kind of data it is intended (RSEM or FPKM), and the test set on which the reported subtyping results were evaluated. The feature spaces of interest moved from the whole sets of profiled genes, to relevant signatures found with our feature selection study. Additionally, as mentioned in the main paper, we used also the already known PAM50 genes as feature space for mLRs; so-obtained results were useful both to be compared with the *AWCA*-based PAM50 classifications and as a benchmark to better interpret and evaluate the performances reached with our alternative gene signatures of interest.

Regularized mLR model trained on TCGA training set – 19737 genes as feature space

Confusion Matrix on TCGA test set

	Classified as Basal	Classified as Her2	Classified as LumA	Classified as LumB	Classified as Normal-like	Recall	Recall %
Ciriello et al. Basal	**84**<br>97.7%	**1**<br>1.2%		**1**<br>1.2%		84/86	97.7%
Ciriello et al. Her2		**12**<br>80.0%		**3**<br>20.0%		12/15	80.0%
Ciriello et al. LumA		**7**<br>1.9%	**300**<br>82.2%	**46**<br>12.6%	**12**<br>3.3%	300/365	82.2%
Ciriello et al. LumB		**5**<br>4.0%	**11**<br>8.7%	**110**<br>87.3%		110/126	87.3%
Ciriello et al. Normal-like	**2**<br>40.0%				**3**<br>60.0%	3/5	60.0%
Precision	84/86	12/25	300/311	110/160	3/15	**597**	
Precision %	97.7%	48.0%	96.5%	68.8%	20.0%		

Overall accuracy	**0.852596**
Micro-averaged precision	0.852596
Macro-averaged precision	0.661775
Micro-averaged recall	0.852596
Macro-averaged recall	0.814336

365 LumA
126 LumB
15 Her2e
86 Basal
5 Normal-like

TEST

Figure S22. Regularized multiclass Logistic Regression: Performances and confusion matrix on TCGA test set for the model trained on TCGA training set using the whole TCGA gene set as feature space.

Regularized mLR model trained on TCGA training set – Top1000 Chi-square based genes as feature space

Confusion Matrix on TCGA test set

	Classified as Basal	Classified as Her2	Classified as LumA	Classified as LumB	Classified as Normal-like	Recall	Recall %
Ciriello et al. Basal	**84**<br>97.7%	**1**<br>1.2%		**1**<br>1.2%		84/86	97.7%
Ciriello et al. Her2		**14**<br>93.3%		**1**<br>6.7%		14/15	93.3%
Ciriello et al. LumA		**6**<br>1.6%	**302**<br>82.7%	**46**<br>12.6%	**11**<br>3.0%	302/365	82.7%
Ciriello et al. LumB		**5**<br>4.0%	**10**<br>7.9%	**111**<br>88.1%		111/126	88.1%
Ciriello et al. Normal-like	**1**<br>20.0%				**4**<br>60.0%	4/5	80.0%
Precision	84/85	14/26	302/312	111/159	4/15	**597**	
Precision %	98.8%	53.8%	96.8%	69.8%	26.7%		

Overall accuracy	**0.862647**
Micro-averaged precision	0.862647
Macro-averaged precision	0.691885
Micro-averaged recall	0.862647
Macro-averaged recall	0.883685

365 LumA
126 LumB
15 Her2e
86 Basal
5 Normal-like

TEST

Figure S23. Regularized multiclass Logistic Regression: Performances and confusion matrix on TCGA test set for the model trained on TCGA training set using the top1000 Chi-square based genes as feature space.

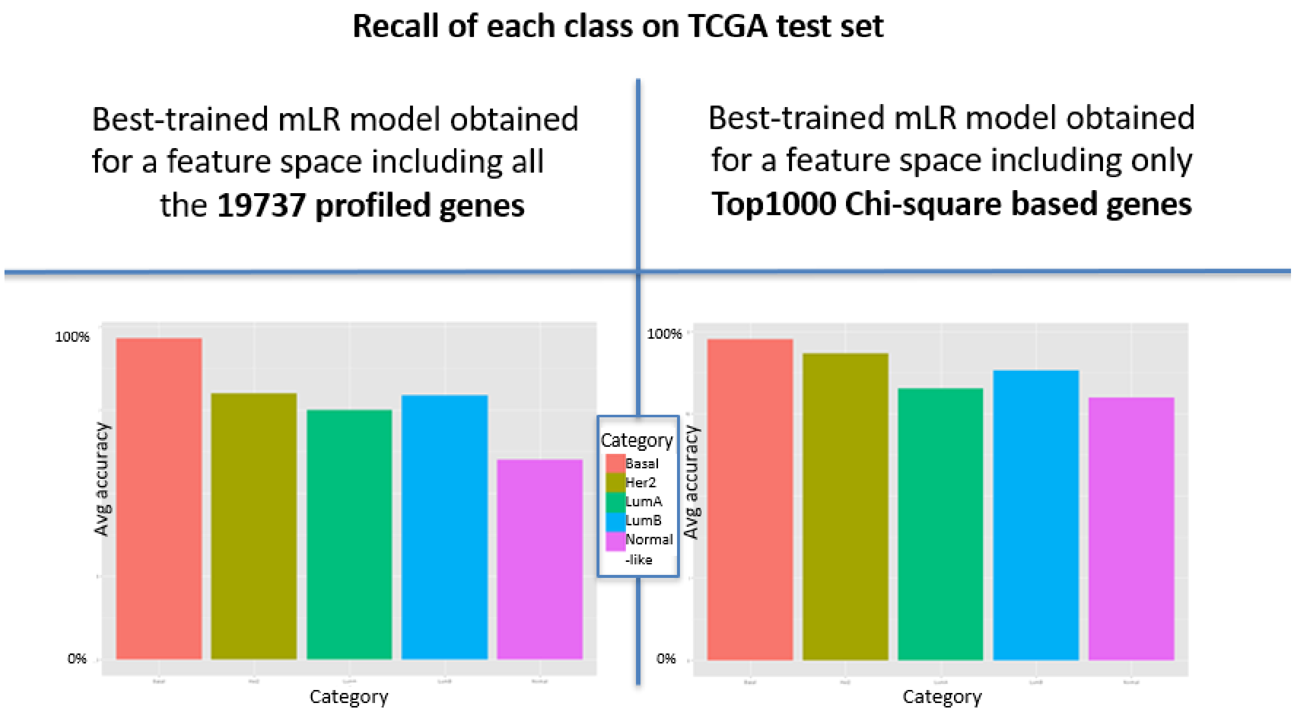

Figure S24. Regularized multiclass Logistic Regression: Recalls of each class on TCGA test set for the models trained on TCGA training set using alternatively the whole TCGA gene set or the top1000 Chi-square based genes as feature space.

Regularized mLR model trained on TCGA training set – limma50 genes as feature space

Confusion Matrix on TCGA test set

	Classified as Basal	Classified as Her2	Classified as LumA	Classified as LumB	Classified as Normal-like	Recall	Recall %
Ciriello et al. Basal	**84** 97.7%	**1** 1.2%		**1** 1.2%		84/86	97.7%
Ciriello et al. Her2		**15** 100.0%				15/15	100.0%
Ciriello et al. LumA		**7** 1.9%	**308** 84.4%	**35** 9.6%	**15** 4.1%	308/365	84.4%
Ciriello et al. LumB		**6** 4.8%	**8** 6.3%	**112** 88.9%		112/126	88.9%
Ciriello et al. Normal-like					**5** 100.0%	5/5	100.0%
Precision	84/84	15/29	308/316	112/148	5/20	**817**	
Precision %	100.0%	51.7%	97.5%	75.7%	25.0%		

Overall accuracy **0.877722**

Micro-averaged precision 0.877722
Macro-averaged precision 0.699736
Micro-averaged recall 0.877722
Macro-averaged recall 0.941894

Signature including 277 genes

365 LumA
126 LumB
15 Her2e
86 Basal
5 Normal-like

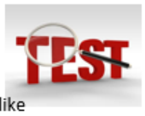

Figure S25. Regularized multiclass Logistic Regression: Performances and confusion matrix on TCGA test set for the model trained on TCGA training set using limma50 gene signature as feature space.

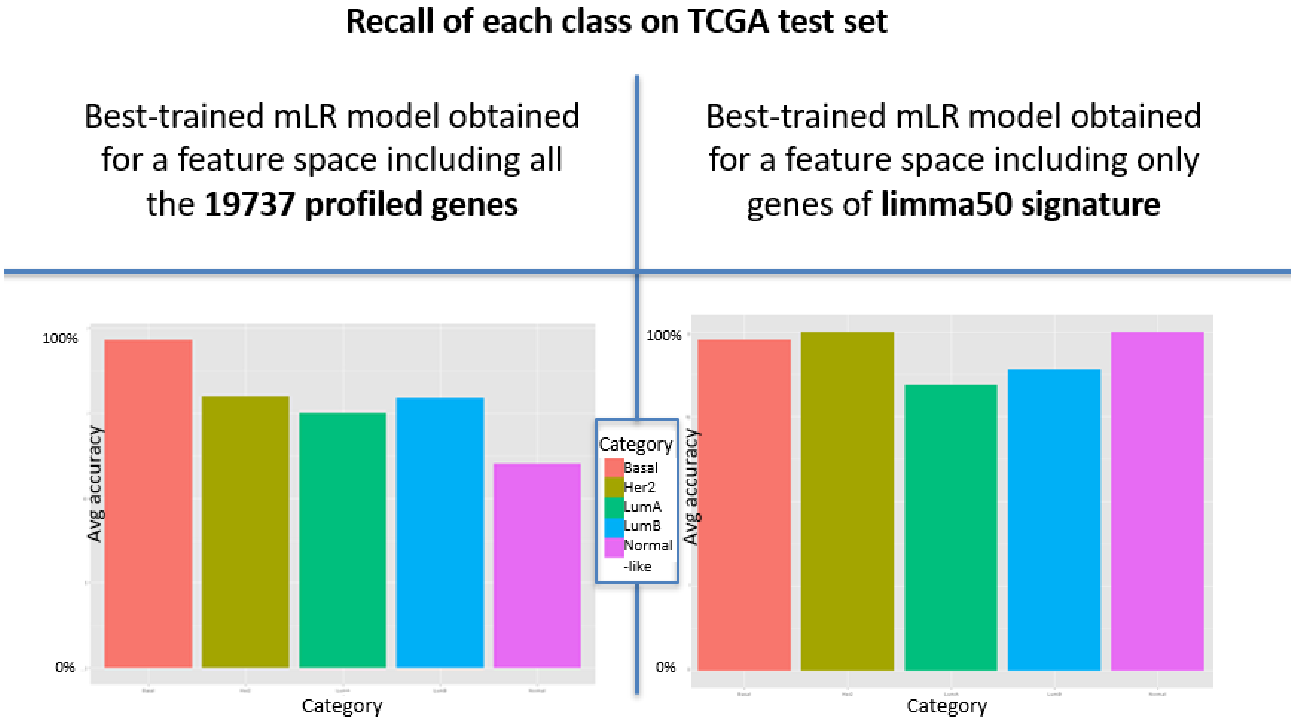

Figure S26. Regularized multiclass Logistic Regression: Recalls of each class on TCGA test set for the models trained on TCGA training set using alternatively the whole TCGA gene set or the limma50 gene signature as feature space.

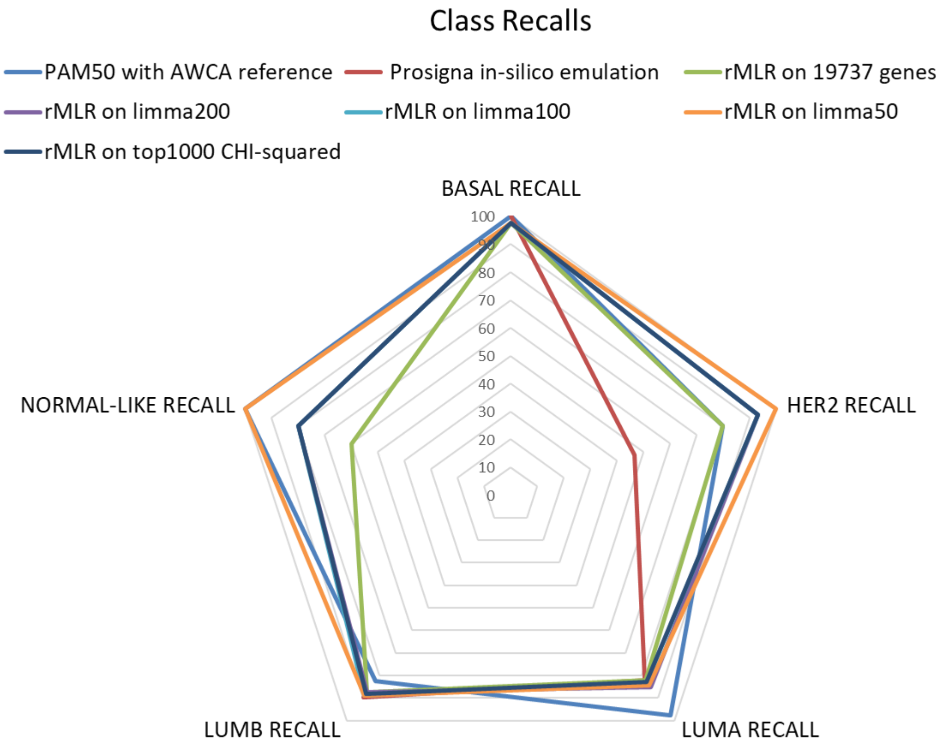

Figure S27. Comparative analysis on TCGA test set: Recalls of each class for different subtyping approaches

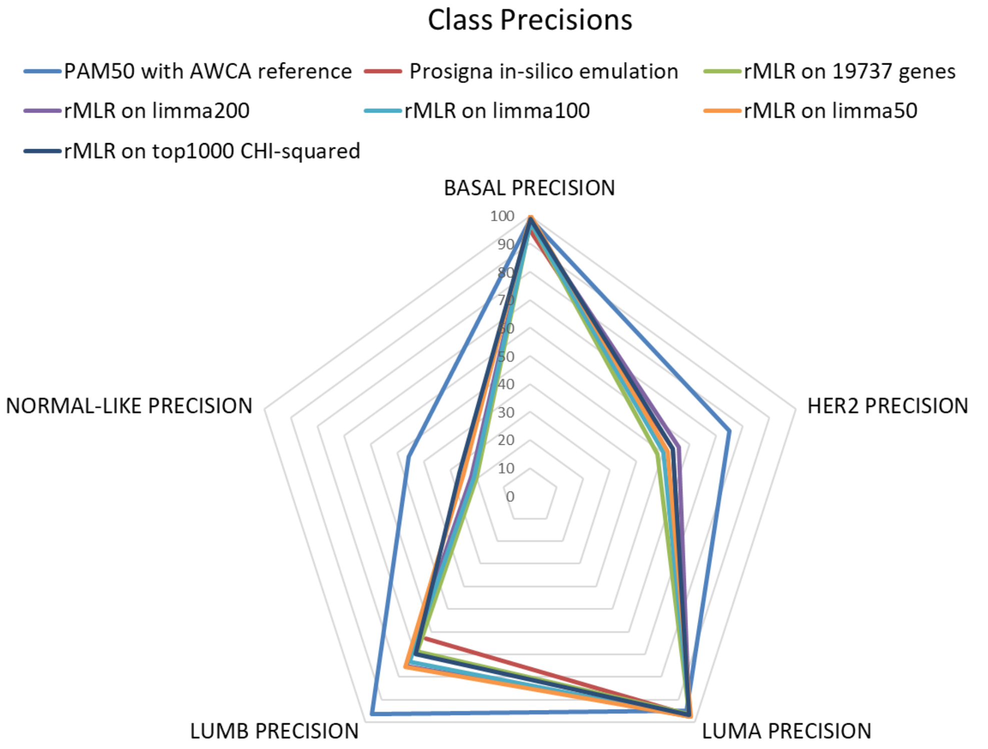

Figure S28. Comparative analysis on TCGA test set: Precisions of each class for different subtyping approaches

Regularized mLR model trained on TCGA training set - limma50 genes as feature space (except DRAIC)

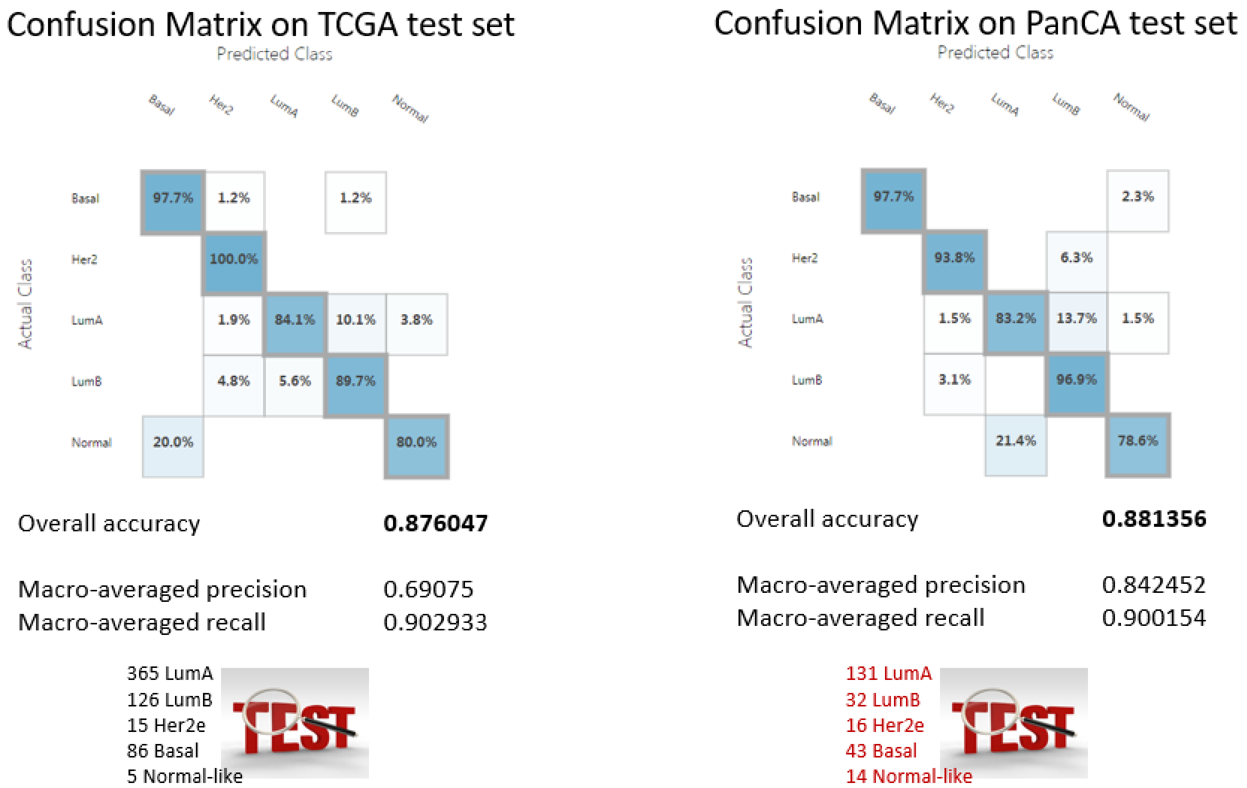

Figure S29. Regularized multiclass Logistic Regression: Testing on the intra-dataset test set and on the external PanCA dataset of the model trained on TCGA training set using limma50 gene signature as feature space

Regularized mLR model trained on TCGA training set - limma50_BWE genes as feature space (except DRAIC)

Confusion Matrix on TCGA test set

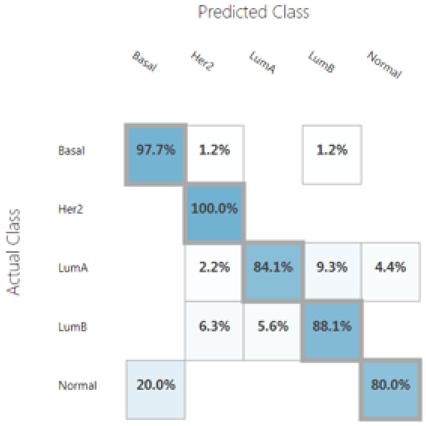

limma50_BWE feature selection strategy
10 runs of in-parallel backward elimination starting from limma50 and then preserving more relevant genes

Confusion Matrix on PanCA test set

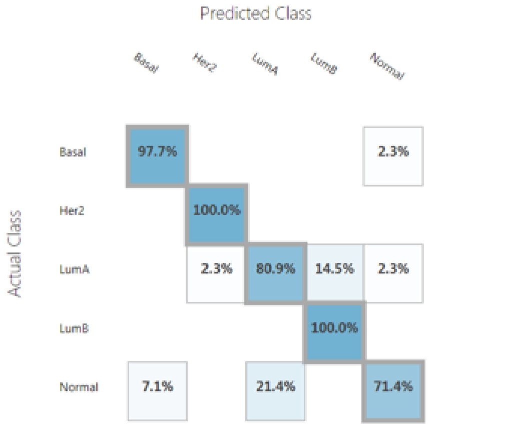

Overall accuracy	**0.872697**
Macro-averaged precision	0.678993
Macro-averaged recall	0.899758

365 LumA
126 LumB
15 Her2e
86 Basal
5 Normal-like

Overall accuracy	**0.872881**
Macro-averaged precision	0.826613
Macro-averaged recall	0.900038

131 LumA
32 LumB
16 Her2e
43 Basal
14 Normal-like

Figure S30. Regularized multiclass Logistic Regression: Testing on the intra-dataset test set and on the external PanCA dataset of the model trained on TCGA training set using limma50_BWE gene signature as feature space

Regularized mLR model trained on TCGA training set – PAM50 genes as feature space
92% of generalization accuracy estimated with 10 fold CV

Confusion Matrix on TCGA test set

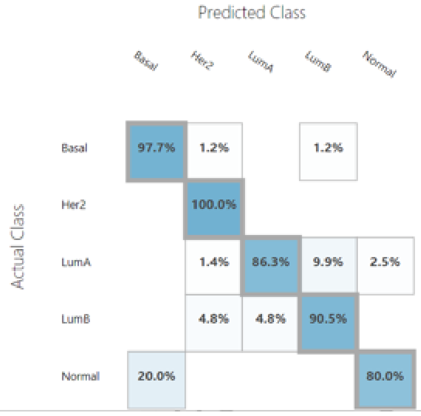

Confusion Matrix on PanCA test set

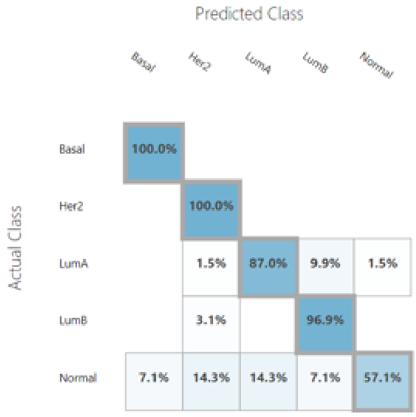

Overall accuracy	**0.891122**
Macro-averaged precision	0.717552
Macro-averaged recall	0.908904

365 LumA
126 LumB
15 Her2e
86 Basal
5 Normal-like

Overall accuracy	**0.898305**
Macro-averaged precision	0.842165
Macro-averaged recall	0.882082

131 LumA
32 LumB
16 Her2e
43 Basal
14 Normal-like

Figure S31. Regularized multiclass Logistic Regression: Testing on the intra-dataset test set and on the external PanCA dataset of the model trained on TCGA training set using PAM50 gene signature as feature space

Table S5. Feature selection study on the TCGA dataset: Main results.

Feature Selection Strategy	**Regularized multiclass Logistic Regression trained on TCGA training set with different feature spaces**	**Generalization accuracy estimated with cross-validation**	**Overall accuracy on TCGA test set**
**-**	ORIGNINAL FEATURE SPACE (**19,737** GENES)	88%	85%
**FILTER METHODS**	TOP **1000** SPEARMAN CORRELATION	89%	87%
	TOP **1000** MUTUAL INFORMATION	89%	86%
	TOP **1000** FISHER SCORES	90%	87%
	TOP **1000** CHI-SQUARED BASED	90%	86%
	TOP **500** CHI-SQUARED BASED	90%	86%
	TOP **200** CHI-SQUARED BASED	89%	86%
**FILTER METHODS + BACKWARD ELIMINATION**	SIGNATURE OF **165 GENES** from TOP 200 Chi-squared + BACKWARD ELIMINATION (10 runs)	91%	86%
	SIGNATURE OF **276 GENES** from TOP 500 Chi-squared + BACKWARD ELIMINATION (10 runs)	94%	86%
**LIMMA ANALYSIS**<br><br>**limmaN:** Union of TOP N Differentially Expressed Genes of each pairwise contrast	SIGNATURE OF **4,168 GENES** - limma1000	90%	84%
	SIGNATURE OF **2,326 GENES** - limma500	89%	86%
	SIGNATURE OF **1,020 GENES** - limma200	93%	88%
	SIGNATURE OF **538 GENES** - limma100	95%	87%
	SIGNATURE OF **277 GENES** - **limma50**	92%	88%
**limma50 + BACKWARD ELIMINATION**	SIGNATURE OF **210 GENES** - **limma50_BWE** (from limma50 + BACKWARD ELIMINATION 10 runs)	93%	87%
**A PRIORI**	**PAM50 genes**	92%	89%

Regularized mLR model trained on GSE96058 training set - 30865 genes as feature space

Confusion Matrix on GSE9658 test set

	Classified as Basal	Classified as Her2	Classified as LumA	Classified as LumB	Classified as Normal-like	Recall	Recall %
Ciriello et al. Basal	**159** 94.1%	**4** 2.4%	**2** 1.2%		**4** 2.4%	159/169	94.1%
Ciriello et al. Her2	**1** 0.6%	**141** 86.5%	**4** 2.5%	**17** 10.4%		141/163	86.5%
Ciriello et al. LumA	**2** 0.2%	**6** 0.7%	**789** 95.3%	**28** 3.4%	**3** 0.4%	789/828	95.3%
Ciriello et al. LumB	**1** 0.3%	**11** 3.0%	**43** 11.8%	**309** 84.9%		309/364	84.9%
Ciriello et al. Normal-like	**4** 3.6%	**6** 5.5%	**39** 35.5%		**61** 55.5%	61/110	55.5%
Precision	159/167	141/168	789/877	309/354	61/68	**1634**	
Precision %	95.2%	83.9%	90.0%	87.3%	89.7%		

Overall accuracy **0.892901**

Micro-averaged precision 0.892901
Macro-averaged precision 0.892196
Micro-averaged recall 0.892901
Macro-averaged recall 0.832441

828 LumA
364 LumB
169 Her2e
163 Basal
110 Normal-like

Figure S32. Regularized multiclass Logistic Regression: Performances and confusion matrix on GSE96058 test set for the model trained on GSE96058 training set using the whole GSE96058 gene set as feature space.

Regularized mLR model trained on GSE96058 training set - limma50 genes as feature space (except DRAIC)

Confusion Matrix on GSE9658 test set

	Classified as Basal	Classified as Her2	Classified as LumA	Classified as LumB	Classified as Normal-like	Recall	Recall %
Ciriello et al. Basal	**157** 92.9%	**8** 4.7%	**2** 1.2%		**2** 1.2%	157/169	92.9%
Ciriello et al. Her2		**150** 92.0%	**3** 1.8%	**10** 6.1%		153/163	92.0%
Ciriello et al. LumA	**2** 0.2%	**4** 0.5%	**793** 95.8%	**24** 2.9%	**5** 0.6%	793/828	95.8%
Ciriello et al. LumB	**1** 0.3%	**10** 2.7%	**37** 10.2%	**316** 86.8%		316/364	86.8%
Ciriello et al. Normal-like	**4** 3.6%	**4** 3.6%	**32** 29.1%		**70** 63.6%	70/110	63.6%
Precision	157/164	150/176	793/867	316/340	70/77	**1634**	
Precision %	95.7%	85.2%	91.5%	92.9%	90.9%		

Overall accuracy **0.909425**

Micro-averaged precision 0.909425
Macro-averaged precision 0.907237
Micro-averaged recall 0.909425
Macro-averaged recall 0.862293

828 LumA
364 LumB
169 Her2e
163 Basal
110 Normal-like

Figure S33. Regularized multiclass Logistic Regression: Performances and confusion matrix on GSE96058 test set for the model trained on GSE96058 training set using limma50 gene signature as feature space.

Regularized mLR model trained on GSE96058 training set - limma50 genes as feature space (except unavailable genes)

Confusion Matrix on GSE9658 test set

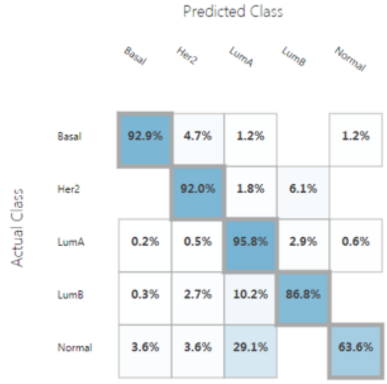

Overall accuracy	**0.909425**
Macro-averaged precision	0.907237
Macro-averaged recall	0.862293

828 LumA
364 LumB
169 Her2e
163 Basal
110 Normal-like

TEST

Confusion Matrix on GSE81538 test set

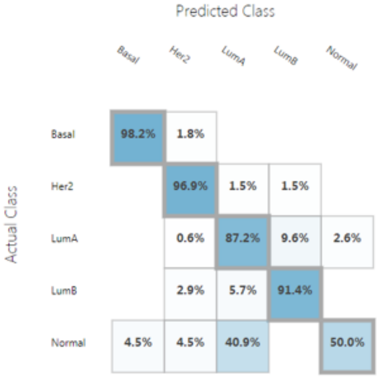

Overall accuracy	**0.893827**
Macro-averaged precision	0.876143
Macro-averaged recall	0.847553

156 LumA
105 LumB
65 Her2e
57 Basal
22 Normal-like

TEST

Figure S34. Regularized multiclass Logistic Regression: Testing on the intra-dataset test set and on the external PanCA dataset of the model trained on TCGA training set using limma50 gene signature as feature space

Regularized mLR model trained on GSE96058 training set - limma50_BWE genes as feature space (except unavailable genes)

Confusion Matrix on GSE9658 test set

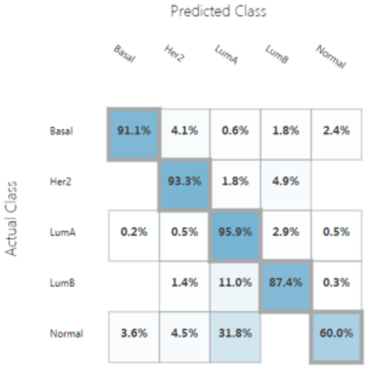

Overall accuracy	**0.908201**
Macro-averaged precision	0.906294
Macro-averaged recall	0.855264

828 LumA
364 LumB
169 Her2e
163 Basal
110 Normal-like

TEST

limma50_BWE feature selection strategy
10 runs of in-parallel backward elimination starting from limma50 and then preserving more relevant available genes

Confusion Matrix on GSE81538 test set

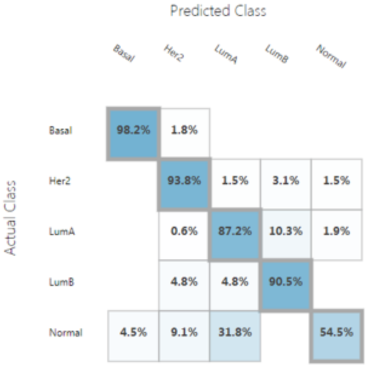

Overall accuracy	**0.888889**
Macro-averaged precision	0.871469
Macro-averaged recall	0.848586

156 LumA
105 LumB
65 Her2e
57 Basal
22 Normal-like

TEST

Figure S35. Regularized multiclass Logistic Regression: Testing on the intra-dataset test set and on the external PanCA dataset of the model trained on TCGA training set using limma50_BWE gene signature as feature space

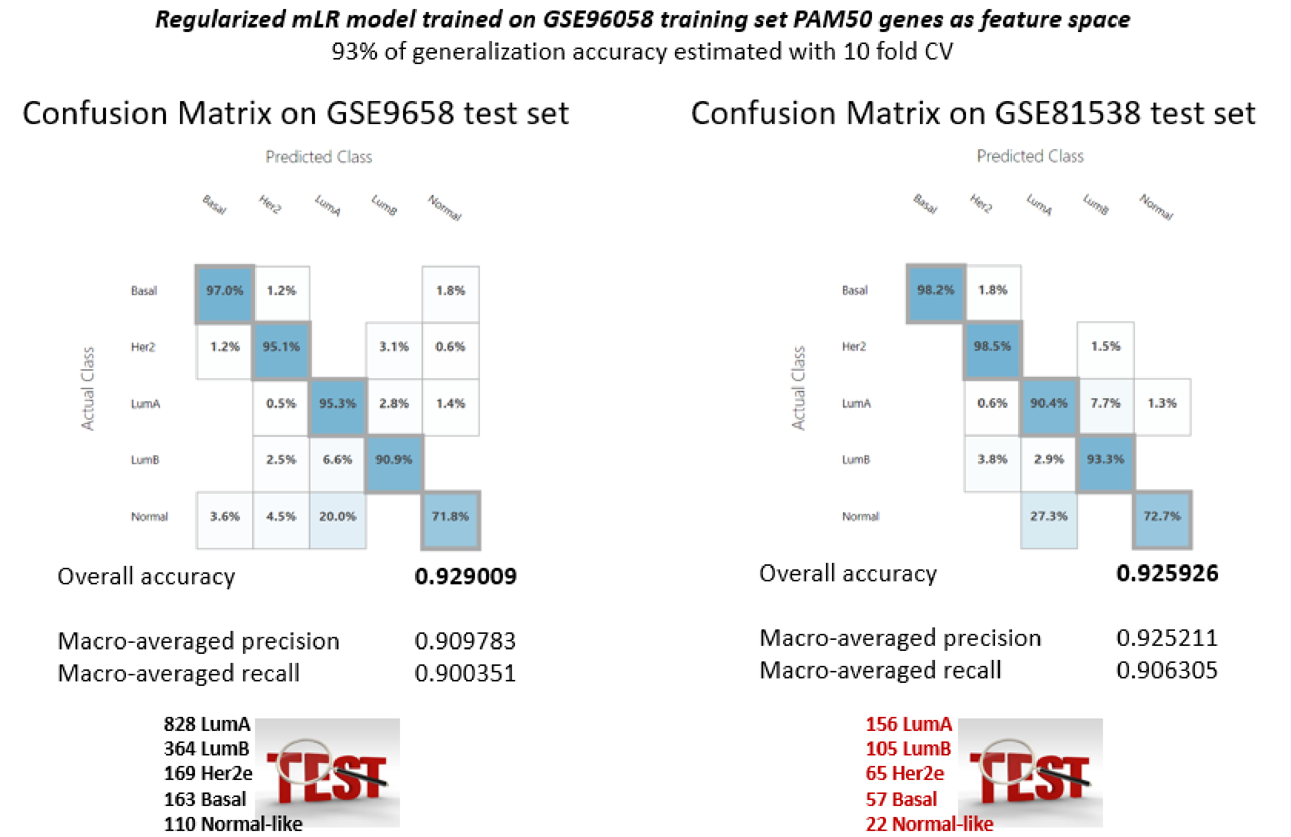

Figure S36. Regularized multiclass Logistic Regression: Testing on the intra-dataset test set and on the external PanCA dataset of the model trained on TCGA training set using PAM50 gene signature as feature space

Table S6. Feature selection study on the GSE96058 dataset: Main results.

Previous feature selection strategy	**Regularized multiclass logistic regression trained on GSE96058 training set with different feature spaces**	**Generalization accuracy estimated with Cross-validation**	**Overall accuracy on GSE96058 Test set**
-	ORIGINAL FEATURE SPACE (**30,865** GENES)	88%	89%
**LIMMA ANALYSIS** Union of TOP 50 Differentially Expressed Genes of each pairwise contrast	**limma50** - SIGNATURE OF 276 GENES excluding unavailable DRAIC gene	90%	91%
**limma50 + BACKWARD ELIMINATION**	**limma50_BWE** - SIGNATURE OF 209 GENES from limma50 + BACKWARD ELIMINATION (10 runs) excluding unavailable DRAIC gene	90%	91%
**A PRIORI**	**PAM50 genes**	93%	93%

Both limma50 and limma50_BWE gene signatures are reported in Table S7. Genes highlighted in pink belong also to limma50_BWE, while genes written in red are in common with the PAM50 signature.

Table S7. List of genes belonging to limma50 and limma50_BWE signatures

ACADSB	IFRD1	TFF1	MPHOSPH6	IL17B	SCUBE2	GSG2	TRPM6	ATP13A5
RHOB	IGF1R	TFF3	SPRY2	C5AR2	EPS15L1	HORMAD1	TMEM86A	GPR144 (ADGRD2)
ART3	KCNJ11	TTK	NPM2	UBE2T	HPSE2	C2ORF88	KIF18B	TEPP
BUB1	KCNMB1	XBP1	NDC80	RACGAP1	ALX4	MIEN1	CLDN19	SFT2D2
CA12	KIFC1	MIA	AGR2	TFCP2L1	DMRTC2	C2ORF40	SMYD1	C2CD4B
CBR3	KRT16	GDF5	SPAG5	NUSAP1	CLSTN2	PARD6B	LINC01105	TMEM220
CCNA2	MAG	FZD9	POLQ	VGLL1	TINAGL1	SLC7A3	SGOL1	GATA3-AS1
CCNB1	MYBL2	SPARCL1	STARD3	GINS2	NCAPG	RERG	ROPN1B	C9ORF170
CYP2B7P	NAT1	SDPR	KIF2C	GALNT7	SOX17	MICALL1	NEK10	C9ORF152
DMD	NEFL	BCAS1	WWP1	BCL11A	DLK2	TSLP	MBOAT1	FAM72B
DUSP7	NEK2	STC2	UBE2C	FAM64A	GGCT	FGD3	AGR3	MIR143HG (CARMN)
EDN3	NPY1R	INPP4B	CAPN11	RNF186	MLPH	TICRR	NXNL2	FAM72D
EPHA2	NTF4	PRC1	WIF1	ROPN1	ARMT1 (C6ORF211)	BOC	NAGS	FAM196B
ESR1	NTRK2	TBX19	PADI2	SIDT1	VPS37B	CENPL	CYP4Z2P	LINC00173
EZH1	PCSK6	KCNQ4	SCRG1	ELOVL2	ASB13	CAPN13	CT62	DRAIC
FANCA	PGR	CCNB2	CLCA4	CDCA8	SHCBP1	PGAP3	SUSD3	
FGFR2	PLK1	EXO1	TPX2	CEP55	MMRN2	LEMD1	SKA1	
FOXC1	FXYD1	AURKB	TBC1D9	HJURP	ZNF552	CGB7	FAM171A1	
FOXM1	PMAIP1	NRG2	SYNM	MCM10	THSD4	KLHL29	RBM24	
FUT3	PRNP	CLCA2	NCAPH	TRPV6	FAM110D	LRRC3B	PRR15	
G6PD	PTGER3	PPM1F	SLC7A8	DEPDC1B	CNTNAP3	MRGPRX3	NUDT8	
GATA3	PTPRZ1	ZNF516	CBX7	SCN3B	LINC00472	TMEM45B	RASGEF1C	
GFRA1	RLN2	GREB1	ORC6	CENPN	DSN1	DEGS2	HID1	
GPM6B	RRM2	ESPL1	TRIM29	DBNDD2	TRPM3	IFFO2	FAM19A3	
GRB7	SCN2B	MELK	SGK3	SLC22A11	CCDC170	IQGAP3	OR2L13	
GRIA4	CX3CL1	GINS1	SLC39A6	RGMA	SLC44A4	OSR1	EOGT	
CXCL3	SFRP1	SEC16A	SPDEF	PAK7 (PAK5)	FAM83D	CMBL	TPRG1	
GSN	SOX10	TROAP	PAMR1	ERGIC1	PPP1R14C	SRSF12	KY	
FOXA1	AURKA	TSPAN1	RGS22	NDRG2	CDCA3	CHODL	OVCH2	
HOXA2	TAC1	KIF20A	GPR160	WDR19	NUF2	RIMS4	STAC2	

S7 Final recap and comparisons of the main intrinsic subtyping approaches

Table S8 summarizes the performances of the most relevant intrinsic subtyping approaches we evaluated and applied in our study. Specifically, it reports both the accuracies on their corresponding internal test set and on the dataset used for external testing. Corresponding internal and external test sets always include gene expression profiles subjected to the same normalization procedure (RSEM or FPKM) of the data used for developing the approach under consideration.

Although regularized mLRs reached slightly lower accuracies compared to the here proposed *AWCA* version (which includes the Normal-like class) of the PAM50 method, this latter one is biased by the PAM50 nature of the published subtypes, assigned via PAM50 assays and thus using the same genes of interest and the same original centroids developed by Parker *et al*. (2009). Using PAM50 genes with mLR models brought valuable performances, despite the emerged ambiguity of the PAM50 labels them-selves, and set benchmark results, considering the same signature involved in the original PAM50 method. Eventually, even if slightly inferior, the performances of the limma50 and limma50_BWE based regularized mLRs are also interesting and worthy of further investigations and evaluations.

Particularly, in Table S8 note that, for both the limma50 and limma50_BWE based mLRs intended for RSEM gene expression data, the accuracies reached on the internal *TCGA* test set and on the corresponding *PanCa* dataset used for external testing are uniform; the couple of mLR classifiers developed for FPKM data showed instead higher results overall, with slightly lower accuracies in the external *GSE81538* test set than in the internal *GSE96058* test set. This only marginal degradation on the unknown and independent samples of the external test set is usual for a good classifier, which is able anyway to tackle the overfitting risk during training. Eventually, in each of the mLRs, we experienced meaningful and quite comparable performances, which confirm the quality of single-sample intrinsic subtype callers developed with regularized Logistic Regression models and their reliability when dealing with unknown samples.

Notably, at https://github.com/DEIB-GECO/BC_Intrinsic_subtyping, we provide the R code needed to use the limma50 and limma50_BWE based classifiers available to classify external samples subject to RSEM or FPKM normalization.

Table S8. Accuracies on internal and external test sets for the most promising intrinsic subtyping approaches

Intrinsic subtyping approach	Dataset used for *AWCA* reference building	Gene set of interest	Intended for	Test set	Accuracy on test set	External test set	Accuracy on external test set
AWCA-PAM50	*TCGA*	PAM50	RSEM	*TCGA*	**93%**	*PanCa*	**96%**
AWCA-PAM50	*GSE96058*	PAM50	FPKM	*GSE96058*	**95%**	*GSE81538*	**96%**
	**Training set**	**Feature space of interest**					
Regularized mLR	*TCGA* training set	PAM50	RSEM	*TCGA* test set	**89%**	*PanCa*	**90%**
Regularized mLR	*TCGA* training set	limma50	RSEM	*TCGA* test set	**88%**	*PanCa*	**88%**
Regularized mLR	*TCGA* training set	limma50_BWE	RSEM	*TCGA* test set	**87%**	*PanCa*	**87%**
Regularized mLR	*TCGA* training set	PAM50	FPKM	*GSE96058* test set	**93%**	*GSE81538*	**92%**
Regularized mLR	*GSE96058* training set	limma50	FPKM	*GSE96058* test set	**91%**	*GSE81538*	**89%**
Regularized mLR	*GSE96058* training set	limma50_BWE	FPKM	*GSE96058* test set	**91%**	*GSE81538*	**89%**

S7.1 Robustness and concordance evaluation between main intrinsic subtyping approaches

Until here, we used mainly the accuracy with respect to the published calls to evaluate our subtyping approaches; nonetheless, although these concordances with the published calls show classification stability, also other assessment must be highlighted to demonstrate more clearly the robustness of the here proposed single-sample approaches, i.e., the AWCA-based PAM50 classification and the most promising mLR classifiers. Particularly, Table S9 clearly highlights the increased robustness of the AWCA-based PAM50, compared with the standard one; conversely, for the mLR approaches, which are perfectly repeatable, Table S10 reports the mean pairwise concordance of classifications provided by mLR PAM50, limma50 and limma50_BWE among them and with the AWCA-based PAM50 one in each internal/external test set. The three mLR classifiers provided high classification robustness in each testing set, despite they consider different feature spaces. Moreover, comparing these three mLR-based classifications with the AWCA-based PAM50 subtyping, we found several cases of full agreement, but discordant with the published subtypes; hence, mLRs appear robust also in classifying ambiguous cases, despite published labels were used for their training. Furthermore, mLRs may be further enhanced in the future through a greater amount of training samples and more reliable labels, as the ones from AWCA-based subtyping.

Table S9. Concordance among multiple runs of standard PAM50 or AWCA-based PAM50, varying the sample subset size of interest

APPROACH	SIZE OF SAMPLE SUBSET INVOLVED IN REFERENCE CONSTRUCTION	MEAN PAIRWISE CONCORDANCE AMONG MULTIPLE RUNS	MEAN PAIRWISE CONCORDANCE WITH PUBLISHED CALLS
Standard PAM50	400 samples	**95.4+/-1.0%**	**85.5+/-0.8%**
Standard PAM50	200 samples	**95.2+/-1.4%**	**85.7+/-2.0%**
Standard PAM50	100 samples	**93.1+/-2.9%**	**84.8+/-3.8%**
AWCA-based PAM50	400 samples	**99.1+/-0.4%**	**91.1+/-0.4%**
AWCA-based PAM50	200 samples	**98.7+/-0.5%**	**91.0+/-0.4%**
AWCA-based PAM50	100 samples	**97.7+/-0.9%**	**91.0+/-1.3%**

Table S10. Concordance of mLR classifiers trained with different feature spaces: PAM50, limma50 and limma50_BWE genes

DATASET	MEAN PAIRWISE CONCORDANCE AMONG THE mLRs	MEAN CONCORDANCE WITH AWCA-BASED PAM50
TCGA TEST	95+/-3%	87+/-1%
PANCA	92+/-4%	90+/-0.5%
GSE96058 TEST	95+/-5%	91+/-0.6%
GSE81538	93+/-3%	90+/-3%

Eventually, we compared our single-sample classifications with an alternative independent subtyping method: the Absolute Intrinsic Molecular Subtyping (AIMS) of Paquet et al. Results of AIMS classifications are fully reported on Table S11; as shown, despite AIMS classification is a single-sample approach, its performances resulted weak. The quite low concordances with both published calls and AWCA-based PAM50 calls denote not very stable subtyping results, differently from what we experienced with AWCA-based PAM50.

Table S11. Concordance comparison of the AIMS classifier with published calls and with each of the here proposed single-sample classifiers

DATASET	AIMS VS PUBLISHED CALLS	AIMS VS AWCA-PAM50	AIMS VS mLR PAM50	AIMS VS mLR LIMMA50	AIMS VS mLR LIMMA50_BWE
TCGA TEST SET	0.768844	0.753769	0.827471	0.839196	0.827471
GSE96058 TEST SET	0.74847	0.723378	0.741126	0.746634	0.746634
PANCA SET	0.775424	0.830508	0.805085	0.838983	0.851695
GSE81538 SET	0.787654	0.765432	0.767901	0.77284	0.760494

S7.2 Prognostic assessment

Eventually, we performed further analyses to evaluate the improved or reduced ability of the proposed classifiers to identify cases with better/worse prognosis, considering the 10-year overall survival annotations available for the datasets at our disposal. Specifically, we considered that each subtype call discordance between Luminal A and another subtype (or vice versa) implies a different expected prognosis. Figure S37 shows the increased prognostic ability of our single-sample classifiers in such prognosis-related discordant cases compared with the published calls.

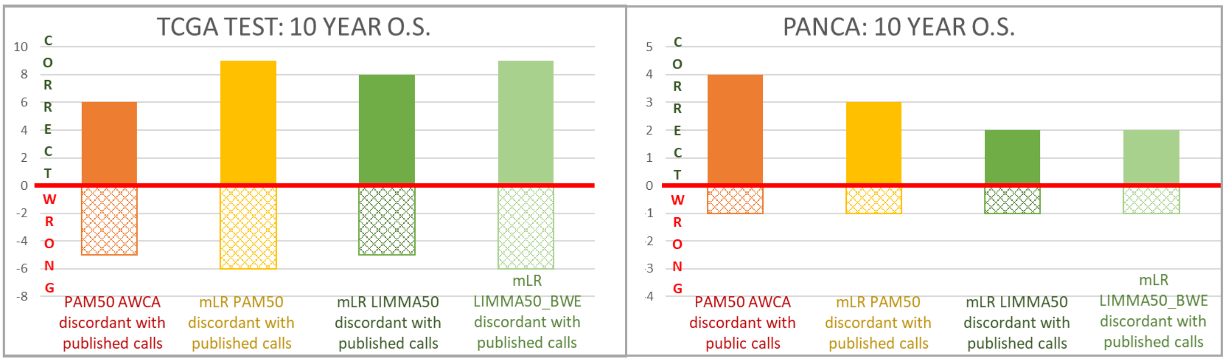

Figure S37. Prognostic ability of proposed classifiers compared with the published calls, considering 10-year overall survival (O.S.).

Conversely, Figures S38 focuses on discordances between the AWCA-based PAM50 classifications and each of the best mLR classifiers, showing a slightly improved prognostic value of mLR classifiers with respect to the AWCA-based PAM50.

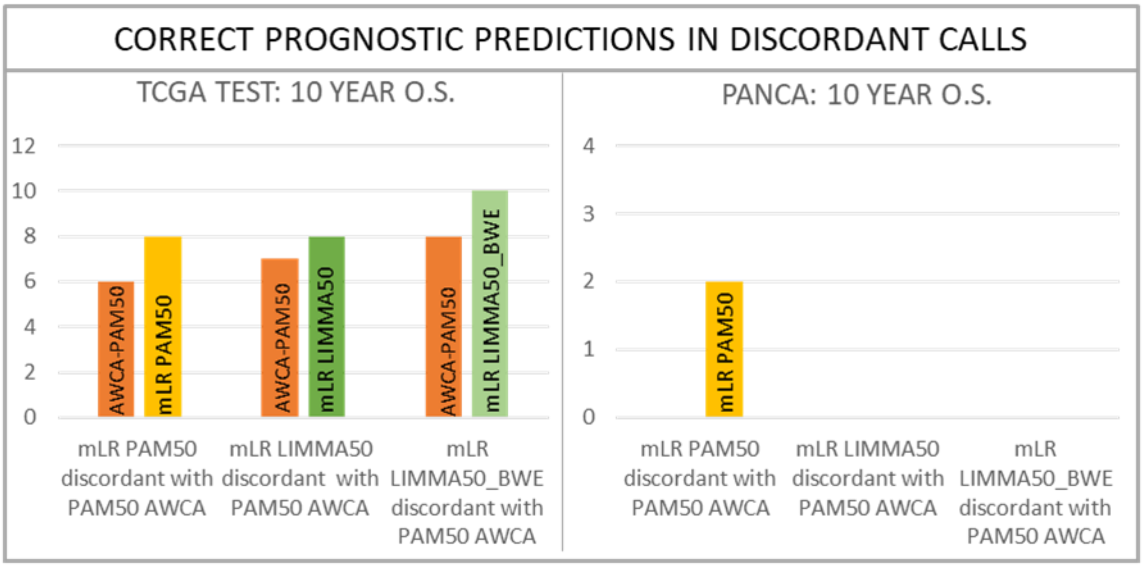

Figure S38. Prognostic ability of mLR classifiers compared with AWCA-based PAM50, considering 10-year overall survival (O.S.).

Thus, in case of discordances, probably due to the already mentioned ambiguity of subtype calls for samples of more difficult attribution, these clinical assessments provided an unbiased criterion to evaluate alternative classifications. Particularly, the emerged results confirmed the reliability of the approaches proposed in this work, in the light of their better capability of recognizing good and poor long-term clinical outcomes.

Appendix

RSEM and FPKM data cross-comparative evaluations

Table S12 summarizes results found in using AWCA references built on RSEM data as external AWCA references for PAM50 classification of FPKM data and vice versa. Comparing these results with those in Table S8 for the AWCA reference with the same normalization of the classified data, we notice the decreased stability of these so-obtained classifications. Thus, for the single-sample AWCA-based PAM50 method we strongly encourage the use of the corresponding normalized AWCA reference, as not to undermine the robustness gain provided by the AWCA approach. Such coherence becomes crucial for the already trained logistic regression models, as clearly exemplified in Table S13; we also evaluated strategies to scale and/or transform the differently normalized public data at our disposal, but performances did not improve, probably due to the introduced approximations and bias.

Table S12. Cross-comparative evaluation of RSEM and FPKM AWCA references for PAM50 subtyping of differently normalized data.

DATASET ON WHICH CROSS-AWCA-BASED PAM50 IS APPLIED	RSEM values of TCGA dataset	RSEM values of TCGA dataset	FPKM values of GSE96058 dataset	FPKM values of GSE96058 dataset
AWCA REFERENCE CONSTRCUTION	AWCA reference built with FPKM data of GSE96058 including Normal-like class	AWCA reference built with FPKM data of GSE96058 excluding Normal-like class	AWCA reference built with RSEM data of TCGA including Normal-like class	AWCA reference built with RSEM data of TCGA excluding Normal-like class
ACCURACY WITH RESPECT TO PUBLISHED CALLS	80.6%	87.4%	87.9%	79.8%

Table 13 Multiclass logistic regression models trained and tested on differently normalized data, using PAM50 genes as feature space.

Approach	Accuracy	Macro-avg recall (balanced accuracy)	Macro-avg precision
	Tested on PANCA RSEM data	Tested on PANCA RSEM data	Tested on PANCA RSEM data
**mLR PAM50 trained on GSE96058 FPKM data**	64%	76%	73%
	Tested on GSE81538 FPKM data	Tested on GSE81538 FPKM data	Tested on GSE81538 FPKM data
**mLR PAM50 trained on TCGA RSEM data**	11% (almost all predicted as Normal-like)	26%	64%

AWCA-based PAM50: an additional use-case on microarray data

We tested the AWCA-based PAM50 method on microarray expression data from Affymetrix U133A-B chips. Our test was easily performed using the R code we made available on GitHub (https://github.com/DEIB-GECO/BC_Intrinsic_subtyping); consequently, we provide also a useful conversion table to trace PAM50 genes in U133A-B and also in U133 PLUS 2.0 GeneChips.

For this additional analysis, we used two public GEO dataset (GSE4922 and GSE1456). For both datasets, raw data were normalized by using the global mean method. Probe-set signal values were natural log transformed and scaled by adjusting the mean intensity to a target signal value. We simply converted natural log values into log2-transfored values and selected for each PAM50 gene the most specific available corresponding probe. In case of ties, we selected the probe bringing the highest mean signal and variance.

The GSE4922 dataset includes 249 samples, without any subtype annotation, and is unbalanced in terms of ER status distribution (211 ER+/34 ER-). Therefore, we compared AWCA-based classifications with corresponding standard PAM50 classifications using 60% ER+/40% ER- subsets of 50 samples. Concordance evaluations show that our AWCA-based approach overcomes the standard one, with mean concordance of 96% versus 88% and lower standard deviation of only 2.0% versus 5.7%.

The GSE1456 dataset includes 139 samples, 119 of which are assigned with intrinsic subtype. Nonetheless, no ER status annotation is available to repeat standard PAM50 classifications with corresponding AWCA-based PAM50 classifications. Therefore, we exported an AWCA reference built in the GSE4922 dataset to the GSE1456 dataset to be used as external reference for subtyping of GSE1456 dataset. This brought lower concordance with respect to the original calls (80/119); nonetheless, from a careful evaluation of our subtyping results against the available 7 year disease free survival annotations, we found that our calls were much more reliable in predicting medium-long clinical outcome than the published calls. Indeed, in all cases of discordances implying changes in expected prognoses, the original classification correctly predicts only 11 cases, whereas our external AWCA-based subtyping provides 37 prognostic reliable subtype calls. Thus, for ambiguous/discordant samples AWCA-based PAM50 resulted to be more than 3 times better in recognizing good or poor prognoses within 7 years.

Overall, both internal and external AWCA-based PAM50 classifications provided higher stability and reliability of subtyping, as well as a substantial increase of prognostic ability, compared with the standard PAM50, confirming the strength of our AWCA approach also on gene expression data from microarray technology.

References

Bastien,R.R.L. *et al.* (2014) Clinical validation of the Prosigna breast cancer prognostic gene signature assay on formalin-fixed paraffin embedded breast cancer tumors with comparison to standard molecular markers. *J. Clin. Oncol.,* **32**(15_suppl), e11518-e11518.

Brueffer,C. *et al.* (2018) Clinical Value of RNA Sequencing-Based Classifiers for Prediction of the Five Conventional Breast Cancer Biomarkers: A Report From the Population-Based Multicenter Sweden Cancerome Analysis Network- Breast Initiative. *J.C.O. Precis. Oncol.,* **2**, 1-18.

Ciriello,G. *et al*. (2015) Comprehensive molecular portraits of invasive lobular breast cancer. *Cell,* **163**(2), 506-519.

Dai,X. *et al.* (2015) Breast cancer intrinsic subtype classification, clinical use and future trends. *Am. J. Cancer Res.,* **5**(10), 2929-2943.

Ein-Dor,L. *et al*. (2005) Outcome signature genes in breast cancer: is there a unique set?. *Bioinformatics*, **21**(2),171-178.

Gao, F. *et al.* (2019) DeepCC: a novel deep learning-based framework for cancer molecular subtype classification. *Oncogenesis*, **8**(9), 1-12.

Giarratana,G. *et al.* (2009) Data mining techniques for the identification of genes with expression levels related to breast cancer prediction. *Proceedings of the 2009.Ninth IEEE International Conference on Bioinformatics and BioEngineering: BIBE 2009*. IEEE Computer Society, Los Alamitos, CA, pp. 295-300.

Holm,J. *et al.* (2017) Assessment of breast cancer risk factors reveals subtype heterogeneity. *Cancer Res.,* **77**(13), 3708-3717.

Koboldt,D.C. *et al.* (2012) Comprehensive molecular portraits of human breast tumours. *Nature,* **490**(7418), 61-70.

Kourou,K. *et al.* (2014) Machine learning applications in cancer prognosis and prediction. *Comput. Struct. Biotechnol. J.,* **13**, 8-17.

Li,B. *et al.* (2011) RSEM: accurate transcript quantification from RNA-Seq data with or without a reference genome. *BMC Bioinformatics*, **12**, 323.

Nielsen,T. *et al.* (2014) Analytical validation of the PAM50-based Prosigna Breast Cancer Prognostic Gene Signature Assay and nCounter Analysis System using formalin-fixed paraffin-embedded breast tumor specimens. *BMC Cancer,* **14**, 177

Ohnstad,H. *et al.* (2017) Prognostic value of PAM50 and risk of recurrence score in patients with early-stage breast cancer with long-term follow-up. *Breast Cancer Res*. **19**(1), 120.

Paquet, E.R. *et al.* (2014) Absolute Assignment of Breast Cancer Intrinsic Molecular Subtype. *J. Natl. Cancer Inst.,* **107**(1), 357.

Parker,J.S. *et al.* (2009) Supervised risk predictor of breast cancer based on intrinsic subtypes. *J. Clin. Oncol.,* **27**(8), 1160-1167.

Perou,C.M. *et al.* (2000) Molecular portraits of human breast tumours. *Nature,* **406(**6797**)**, 747-752.

Ritchie,M.E. *et al.* (2015). limma powers differential expression analyses for RNA-sequencing and microarray studies. *Nucleic Acids Res.,* **43**(7), e47.

Sørlie,T. *et al.* (2003) Repeated observation of breast tumor subtypes in independent gene expression data sets. *Proc. Natl. Acad. Sci.,* **100**(14), 8418-8423.

Sørlie,T. *et al.* (2001) Gene expression patterns of breast carcinomas distinguish tumor subclasses with clinical implications. *Proc. Natl. Acad. Sci.,* **98**(19),10869-10874.

Sumbaly,R. *et al.* (2014) Diagnosis of breast cancer using decision tree data mining technique. *Int. J. Comput. Appl.,* **98**(10), 16-24.

Tibshirani,R. *et al.* (2002) Diagnosis of multiple cancer types by shrunken centroids of gene expression. *Proc. Natl. Acad. Sci.*, **99**(10), 6567-6572.

Trapnell,C. *et al.* (2010) Transcript assembly and abundance estimation from RNA-Seq reveals thousands of new transcripts and switching among isoforms. *Nat. Biotechnol*. **28**, 511–515.

Vallon-Christersson J. *et al.* (2019) Cross comparison and prognostic assessment of breast cancer multigene signatures in a large population-based contemporary clinical series. Scientific reports. **9**(1):1-6.

Vieira,A.F. *et al*. (2018) An Update on Breast Cancer Multigene Prognostic Tests - Emergent Clinical Biomarkers. *Front. Med. (Lausanne)*, **5**, 248.

Wallden,B. *et al.* (2015) Development and verification of the PAM50-based Prosigna breast cancer gene signature assay. *BMC Med. Genomics*, **8**, 54.

Waldemarson,S. *et al*. (2016). Proteomic analysis of breast tumors confirms the mRNA intrinsic molecular subtypes using different classifiers: a large-scale analysis of fresh frozen tissue samples. *Breast Cancer Res*., **18**(1), 69.

Yersal,O. *et al.* (2014) Biological subtypes of breast cancer: Prognostic and therapeutic implications. *World J Clin Oncol.,* **5**(3), 412–424.
